# Deterministic succession patterns in the rumen and fecal microbiome associate with host metabolic shifts in peripartum dairy cattle

**DOI:** 10.1093/gigascience/giaf042

**Published:** 2025-05-19

**Authors:** Shuo Wang, Fanlin Kong, Dongwen Dai, Chen Li, Yangyi Hao, Erdan Wang, Zhijun Cao, Yajing Wang, Wei Wang, Shengli Li

**Affiliations:** State Key Laboratory of Animal Nutrition and Feeding, Department of Animal Nutrition and Feed Science, College of Animal Science and Technology, China Agricultural University, Beijing 100193, China; State Key Laboratory of Animal Nutrition and Feeding, Department of Animal Nutrition and Feed Science, College of Animal Science and Technology, China Agricultural University, Beijing 100193, China; State Key Laboratory of Animal Nutrition and Feeding, Department of Animal Nutrition and Feed Science, College of Animal Science and Technology, China Agricultural University, Beijing 100193, China; State Key Laboratory of Animal Nutrition and Feeding, Department of Animal Nutrition and Feed Science, College of Animal Science and Technology, China Agricultural University, Beijing 100193, China; State Key Laboratory of Animal Nutrition and Feeding, Department of Animal Nutrition and Feed Science, College of Animal Science and Technology, China Agricultural University, Beijing 100193, China; State Key Laboratory of Animal Nutrition and Feeding, Department of Animal Nutrition and Feed Science, College of Animal Science and Technology, China Agricultural University, Beijing 100193, China; State Key Laboratory of Animal Nutrition and Feeding, Department of Animal Nutrition and Feed Science, College of Animal Science and Technology, China Agricultural University, Beijing 100193, China; State Key Laboratory of Animal Nutrition and Feeding, Department of Animal Nutrition and Feed Science, College of Animal Science and Technology, China Agricultural University, Beijing 100193, China; State Key Laboratory of Animal Nutrition and Feeding, Department of Animal Nutrition and Feed Science, College of Animal Science and Technology, China Agricultural University, Beijing 100193, China; State Key Laboratory of Animal Nutrition and Feeding, Department of Animal Nutrition and Feed Science, College of Animal Science and Technology, China Agricultural University, Beijing 100193, China

**Keywords:** microbiome, dynamics, longitudinal study, cow, metabolic phenotypes, transition period

## Abstract

**Background:**

Metabolic disorders in peripartum ruminants affect health and productivity, with gut microbiota playing a key role in host metabolism. Therefore, our study aimed to characterize the gut microbiota of peripartum dairy cows to better understand the relationship between metabolic phenotypes and the rumen and fecal microbiomes during the peripartum period.

**Results:**

In a longitudinal study of 91 peripartum cows, we analyzed rumen and fecal microbiomes via 16S rRNA and metagenomic sequencing across six time points. By using enterotype classification, ecological model, and random forest analysis, we identified distinct deterministic succession patterns in the rumen and fecal microbiomes (rumen: rapid transition–transition–stable; hindgut: stable–transition–stable). Key microbes, such as *Succiniclasticum* and *Bifidobacterium*, were found to drive microbial succession by balancing stochastic and deterministic processes. Notably, we observed that changes in gut microbiota succession patterns significantly influenced metabolic phenotypes (e.g., serum non-esterified fatty acid, glucose, and insulin levels). Mediation analysis suggested that specific gut microbes (e.g., *Prevotella sp900315525* in the rumen and *Alistipes sp015059845* in the hindgut) and metabolic pathways (e.g., glucose-related pathway) were associated with host metabolic phenotypes.

**Conclusions:**

Overall, utilizing a large gut microbiome dataset and enterotype- and ecological model-based microbiome analyses, we comprehensively elucidated the succession and assembly of the gut microbiota in peripartum dairy cows. We further confirmed that changes in gut microbiota succession patterns were significantly related to the metabolic phenotypes of peripartum dairy cows. These findings provide valuable insights for developing health management strategies for peripartum ruminants.

## Data Description

We conducted a 42-day dynamic follow-up study on 91 healthy peripartum dairy cows, tracking changes in the rumen microbiome (476 16S rRNA sequencing samples and 30 metagenomic sequencing samples), fecal microbiome (506 16S rRNA sequencing samples and 30 metagenomic sequencing samples), and metabolic phenotypes (505 samples). We established relationships between changes in the gut microbiome and metabolic phenotypes. The metagenomic and 16S rRNA sequencing data used in this study have been archived in the NCBI database under accession numbers PRJNA1161368 and PRJNA1126601, respectively.

## Introduction

Ruminants play a crucial role in global food supply and sustainable agriculture [1]. The peripartum period, defined as 21 days before until 21 days after calving, is one of the most vulnerable times in a ruminant life [2]. During this phase, due to calving, dietary changes, and onset of lactation, ruminants undergo substantial physiological and metabolic adjustments [3–5]. Approximately 30–50% of dairy cows experience postpartum metabolic diseases, including ketosis, hypocalcemia, and retained placenta [5, 6]. These conditions affect the health and productivity of ruminants and lead to substantial economic losses [7].

Ruminants have evolved a unique rumen structure that enables multiple host–microbiome interactions, most significantly, host–rumen microbiome and host–fecal microbiome interactions. The rumen microbiome is essential for cellulose breakdown, short-chain fatty acid production, nitrogen cycling, and vitamin synthesis [8]. The fecal microbiome contributes to energy and nutrient absorption and modulates the host immune system [9]. Additionally, extensive research in adult dairy cows showed that the rumen and fecal microbiomes affect milk quality and feed efficiency [10, 11], further highlighting the critical role of these microbiomes in ruminant production and health.

Recent advances in microbial ecology have brought heightened concerns to the temporal succession and ecological assembly of gut microbiomes. Specifically, microbial succession delineates systematic compositional shifts across developmental stages, while microbiome assembly encompasses deterministic processes that establish functionally coherent communities through multilevel interactions spanning host physiology, microbial cross-feeding, and environmental modulation [12, 13]. This process includes colonization of various microbial species and their interactions with each other and the host environment [13]. Understanding microbial succession and assembly is crucial for comprehending how microbial communities are established stably and functionally. This helps understand how these microbial communities impact health. The TEDDY study and Xiao et al. identified that the infant gut microbiome can be roughly divided into three successive stages: development, transition, and stable [14, 15]. Additionally, they used neutral ecological models to elucidate the transformation patterns and driving forces of the infant gut microbiome [15]. Furman et al. also found that under the deterministic conditions of diet and age in dairy cows, stochastic effects drive the succession and assembly of the rumen microbiome throughout the cow’s life [16]. Similarly, studies on chicks [17], piglets [18], lambs [19], and calves [20, 21] have highlighted how gut microbiome assembly affects the growth and development of young animals. These studies used large-scale longitudinal methods to explore the succession and assembly patterns of the gut microbiome and their dynamic interactions with the host, providing targeted evidence for promoting healthy development and pregnancy via gut microbiome regulation.

Recently, some studies have attempted to reveal the succession patterns of the rumen and fecal microbiome in peripartum ruminants (primarily focusing on cows) [22–26]. However, due to limitations in sample size and temporal resolution, different studies have found varying patterns of change in the gut microbiome of dairy cows. Zhu et al. observed a decrease in the richness of rumen microbiota from the prenatal to the postnatal stages [23]. In contrast, Bach et al. found an increase in the richness of rumen microbiota [24]. Moreover, although Zhu et al. noted that the prepartum fecal microbiota exhibits higher diversity, primarily composed of Firmicutes and Bacteroidetes [25], Luo et al. reported no marked differences in diversity before and after calving, with slight differences in phylum composition [26]. Although these studies indicate varying results, they consistently suggest the potential for remodeling the gut microbiome in peripartum cows. Considering the substantial metabolic changes during the peripartum period, there remains a gap in our understanding of the succession patterns of rumen and fecal microbiomes and their impact on host metabolism. Additionally, exploring key factors driving these microbiome dynamics is crucial. Understanding these factors could potentially allow us to predict and determine when and how to intervene in the microbiome assembly process to modulate its structure and function. In addition to normal dynamic changes, individual factors (e.g., parity and body condition) have previously been reported to correlate with ruminant gut microbiomes [27, 28]. A comprehensive analysis of these factors will aid in better understanding the dynamic changes of the microbiome in peripartum ruminants.

In this study, we used dairy cows with highly controlled feeding systems, diets, and housing as our subjects. By conducting a longitudinal observation of the gut microbiome and host metabolic indicators of dairy cows 21 days before until 21 days after calving, we aim to enhance our detailed understanding of the gut microbiome and host metabolic characteristics in peripartum ruminants, potentially facilitating the development of new strategies to improve postpartum health in ruminants.

## Results

### Dynamic changes in gut microbial composition in peripartum dairy cows

**Figure 1:**
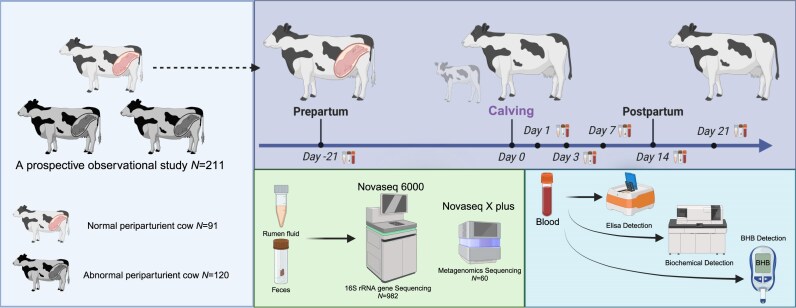
Profiling gut microbiome changes in peripartum dairy cows and their connection to host metabolism: Workflow. BHB: β-hydroxybutyrate.

Through a rigorous prospective cohort study design (Fig. 1), we observed a clear separation of gut microbiota in peripartum dairy cows among the sampling time points, underscoring the remodeling of the cow gut microbiota during peripartum periods (Supplementary Figs S1a and S2a). The α-diversity (Chao1 and Shannon indexes) of ruminal microbiota increased 21 days before until 1 day after calving, decreased from 1 to 7 days after calving, and stabilized from 7 to 21 days after calving (Supplementary Fig. S1b). Conversely, the α-diversity of fecal microbiota decreased 21 days before until 3 days after calving, increased from 3 to 7 days after calving, and stabilized from 7 to 21 days after calving (Supplementary Fig. S2b). We also observed significant changes in the α-diversity of rumen microbiota at just 1 day postpartum. Conversely, fecal microbiota showed a similar response but at 3 days postpartum. Additionally, in both rumen and fecal samples, the dominant microbial phyla were Firmicutes, Bacteroidetes, Actinobacteria, Spirochaetes, and Proteobacteria (Supplementary Figs S1c and S2c).

**Figure 2:**
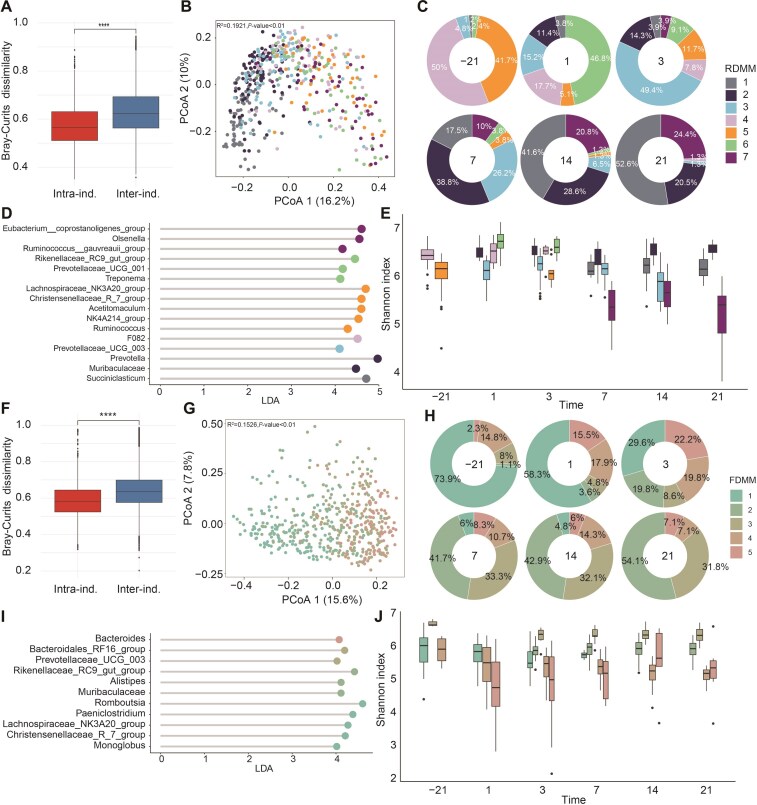
Classification of the rumen and fecal microbiome in peripartum dairy cows based on microbial community clusters. Boxplots showing intra- and interindividual Bray–Curtis dissimilarity in microbiome profiles: (A) Rumen and (F) feces. The PCoA plot-based Bray–Curtis dissimilarity of microbiome profiles across different DMMs: (B) rumen and (G) feces. DMM represents a microbial community cluster. Ring charts showing the distribution of DMMs, with the numbers in the middle of the ring charts representing the sampling time points: (C) rumen and (H) feces. LEfSe reveals the key genera of different DMMs: (D) rumen and (I) feces. Temporal changes of Shannon index in each DMM: (E) rumen and (J) feces.

Importantly, we observed significantly greater interindividual than intraindividual variability across both rumen and fecal microbiota (Fig. 2A and F), and the interindividual variation in the rumen and fecal microbiota constituted 26.14% and 24.96% of the total compositional variation, respectively (permutational multivariate analysis of variance (PERMANOVA), *P* < 0.01, Supplementary Figs S1a and S2a). To more accurately demonstrate microbial succession in the rumen fluid and feces of peripartum cows, we applied the Dirichlet multinomial mixture (DMM) method. At the genus level, based on the lowest Laplace approximation scores (Supplementary Figs S1d and S2d), the analysis yielded 7 and 5 DMM clusters for rumen and fecal samples, respectively (Fig. 2B and G). Regarding ruminal microbiota, *Prevotella, NK4A214 group, Lachnospiraceae NK3A21 group, Acetitomaculum*, and *Succiniclasticum* were the top five genera defining RDMMs (DMM clusters for rumen microbiota; Supplementary Fig. S1e). The heatmap in Supplementary Fig. S1f displays the distribution of these genera across different RDMMs. Each RDMM exhibited a unique timing of appearance and dominant genera (Fig. 2C and D). RDMM1 was dominated by *Succiniclasticum*, prominently appearing until 7 days postpartum, and its proportion increased from 7 to 21 days postpartum; RDMM2, characterized by *Muribaculum* and *Prevotella*, first appeared on day 1 postpartum, with its proportion increasing from 1 to 7 days postpartum, and then gradually decreasing from 7 to 21 days postpartum; RDMM3, dominated by *Prevotellaceae UCG-003*, was present before parturition, with its proportion increasing from prepartum to 3 days postpartum, and then gradually decreasing from 3 to 21 days postpartum; On the other hand, RDMM4 and RDMM5 were primarily active on day 21 prepartum and gradually diminished postpartum. Specifically, *FO82* was identified as the dominant genus for RDMM4, while *Lachnospiraceae NK3A21 group, Christensenellaceae R-7 group, Acetitomaculum, NK4A214 group*, and *Ruminococcus* were the dominant genera for RDMM5; Finally, RDMM6 emerged on day 1 postpartum with dominant genera including *Rikenellaceae RC9 gut group, Prevotella UCG-001*, and *Treponema*, gradually diminishing over the time. By day 3 postpartum, RDMM7, characterized by the *Eubacterium coprostanoligenes group, Olsenella*, and the *Ruminococcus gauvreauii group*, began to appear, and gradually increased over the timepoints. In addition to the occurrence windows, we noted the changing community characteristics of the rumen clusters over the time. The dominant clusters shifted from RDMM4 and RDMM5 prepartum to RDMM6 on 1 day postpartum, then to RDMM3 by 3 days, RDMM2 by 7 days, and finally to RDMM1 by 14 and 21 days (Fig. 2C). Further analysis indicated that except for appearing to decline significantly in RDMM3 at 14 days postpartum, the Shannon diversity of the other RDMM clusters remained relatively stable across the different timepoints (Fig. 2E).

In the fecal microbiota, the top five genera defining FDMMs (DMM clusters for fecal microbiota) were *UCG-005*, the *Rikenellaceae RC9 gut group, Romboutsia, Bifidobacterium*, and *UCG-010* (Supplementary Fig. S2e). The heatmap in Fig. S2f illustrates the distribution of these genera across different FDMMs. Similarly, Each FDMM also exhibited a unique timing of appearance and dominant genera (Fig. 2H and I). FDMM1, characterized by dominant genera, including *Romboutsia, Paeniclostridium*, the *Lachnospiraceae NK3A20 group*, and the *Christensenellaceae R-7 group*, was primarily observed at 21 days prepartum; the proportion of FDMM1 gradually decreased, and finally disappeared postpartum. FDMM2 and FDMM3 appeared prepartum, and their proportion increased postpartum. FDMM2 was predominantly defined by the *Rikenellaceae RC9 gut group, Muribaculaceae*, and *Alistipes*; FDMM3 was dominated by the*Bacteroidales RF16 group* and *Prevotellaceae UCG-003*. FDMM4 did not exhibit any dominant genera, indicating its instability, whereas FDMM5 was dominated by Bacteroides. The two clusters were present throughout the peripartum period, with a peak at 3 days postpartum. Importantly, the community characteristics of fecal clusters varied over the timepoints (Fig. 2H). FDMM1 was dominant at 21 days prepartum and 1 days postpartum, experiencing a shift on 3 days postpartum with no dominant cluster, and stabilized by 7–21 days postpartum, with FDMM2 and FDMM3 becoming dominant. The Shannon index of FDMM1, FDMM2, and FDMM3 initially decreased and then stabilized, whereas that of FDMM4 continued to decrease, and that of FDMM5 fluctuated (Fig. 2J).

### Dynamics of the individual gut microbiota in peripartum cows

**Figure 3:**
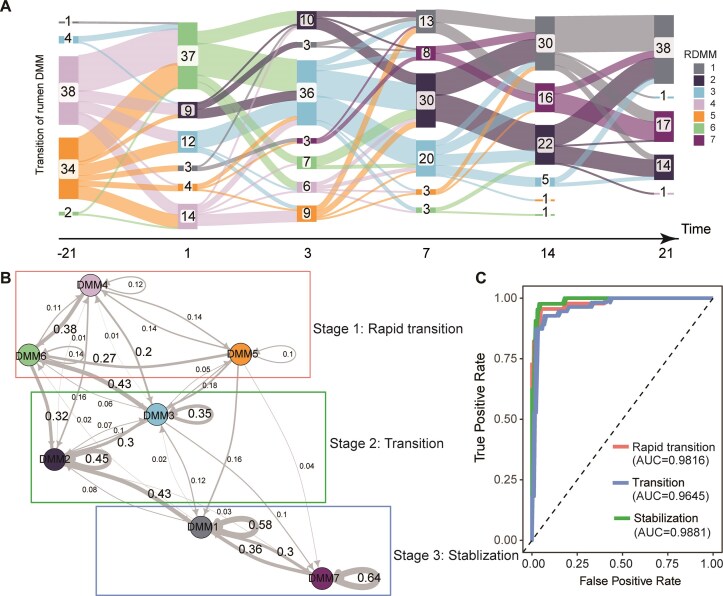
Temporal dynamics of ruminal DMMs in peripartum dairy cows. (A) Sanky diagram showing the transition of ruminal DMMs across six sampling timepoints. (B) Markov chain with subject-independent transition probabilities among ruminal DMMs, in which arrow weights are proportional to the maximum likelihood estimate of the transition probabilities among different states. Ruminal DMMs in different colored boxes are in different stages of microbial succession. The numbers represent the conversion rates across different ruminal DMMs. The numbers represent the conversion rates across different ruminal DMMs. (C) Receiver operating characteristic curve demonstrating the accuracy of the classification model for the successional stages in the rumen microbiome of peripartum dairy cows.

**Figure 4:**
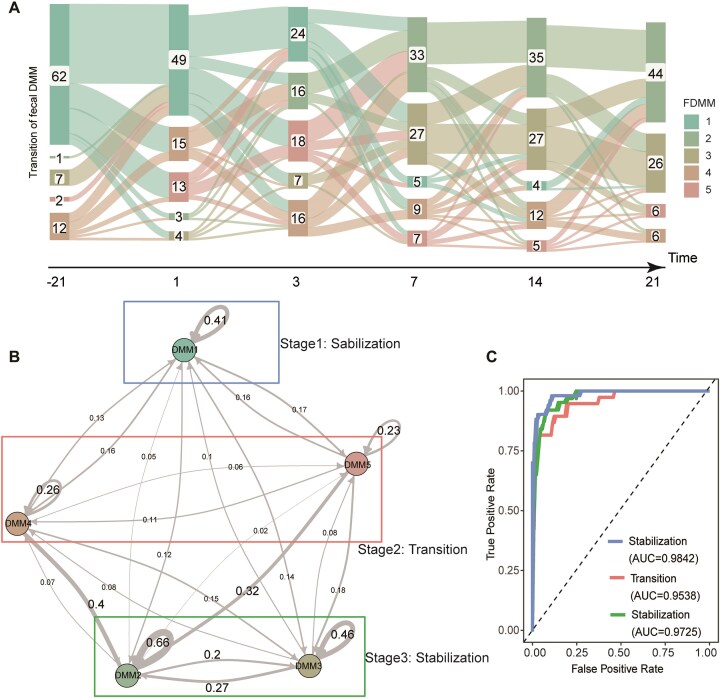
Temporal dynamics of fecal DMMs in peripartum dairy cows. (A) Sanky diagram showing the transition of fecal DMMs across six sampling timepoints. (B) Markov chain with subject-independent transition probabilities among fecal DMMs, in which arrow weights are proportional to the maximum likelihood estimate of the transition probabilities among different states. Fecal DMMs in different colored boxes are in different stages of microbial succession. The numbers represent the conversion rates across different fecal DMMs. The numbers represent the conversion rates across different fecal DMMs. (C) Receiver operating characteristic curve demonstrating the accuracy of the classification model for the successional stages in the fecal microbiome of peripartum dairy cows.

To delve deeper into the transformation process of microbial community clusters in the rumen and feces in 91 peripartum cows, we analyzed their transitions across different sampling days at the individual level. We observed a distinct trend of transitions between the community clusters throughout the study period (Figs 3A and 4A; Supplementary Tables S1 and S2). Specifically, from 21 days prepartum to 1 day postpartum, the RDMM4 to RDMM6 and RDMM5 to RDMM6 transitions represented 24.1% and 16.5% of the total transitions in the rumen, respectively, highlighting them as the dominant transformations (Supplementary Table S1). Similarly, the RDMM6 to RDMM3 transition was the dominant transformation (23.6%) from 1 day to 3 days postpartum; RDMM3 to RDMM2 (19.2%) and RDMM3 to RDMM3 (17.8%) were the dominant transformations from 3 days to 7 days postpartum; RDMM2 to RDMM1 (16.4%) and RDMM2 to RDMM2 (19.2%) were the dominant transformations from 7 days to 14 days postpartum; and RDMM1 to RDMM1 (28.2%) was the dominant transformation from 14 days to 21 days postpartum (Supplementary Table S1). Regarding fecal microbiota, the FDMM1 to FDMM1 transition (41.7%) was the dominant transformation from 21 days prepartum to 3 days postpartum. Interestingly, we did not observe any dominant transformations from 3 days to 7 days postpartum, highlighting the instability of the fecal microbial community structure during this period; however, the FDMM2 to FDMM2 and FDMM3 to FDMM3 transitions dominated from 7 days to 21 days postpartum (Table S2).

To quantitatively integrate the transitions of microbial clusters in the rumen and feces during the peripartum period, we developed a Markov chain model (Figs 3B and 4B). For rumen, we found that RDMM2–6 had high frequencies of transitioning to other clusters, with RDMM4–6 exhibiting lower self-transition rates (below 20%), likely contributing to their rapid disappearance during the succession process. Conversely, RDMM2 and RDMM3 exhibited self-transition rates of 45% and 35%, respectively. These two RDMMs were more stable and may thus play a role in bridging microbial succession. In addition, RDMM1 and RDMM7, with self-transition rates of 58% and 64%, respectively, and mutual transition rates above 30%, exhibited stability and maturity during the later stages of the peripartum period. Thus, peripartum microbial succession could be divided into three phases: rapid transition (RDMM4–6), transition (RDMM3 and RDMM2), and stabilization (RDMM1 and RDMM7). Based on the above microbial succession patterns, we used a random forest algorithm to construct a classification model. Following 5 rounds of 10-fold cross-validation, we determined that the model constructed using the top 100 amplicon sequence variants (ASVs) with the highest accuracy exhibited the highest prediction rate (AUC > 0.95) (Supplementary Fig. S3a and Fig. 3C). In addition, we found that the genera, including the*Rikenellaceae RC9 gut group, Prevotella, Acetitomaculum*, and *F082*, played key roles in constructing the classification model (Supplementary Fig. S3a).

In the study of fecal microbiota during the peripartum period, we also observed distinct self-transfer rates among FDMMs. Specifically, FDMM4 and FDMM5 exhibited lower self-transfer rates of 26% and 23%, respectively. In contrast, FDMM1, FDMM3, and FDMM2 displayed higher rates of 41%, 46%, and 66%, respectively (Fig. 4B). In addition, the conversion rate of FDMM4 and FDMM5 to FDMM1 reached 16% (Fig. 4B). The mutual transfer rates between FDMM2 and FDMM3 exceeded 20%, reflecting their relative stability and maturity during the microbial succession process. Compared with rumen microbiota, fecal microbiota transitioned into a stable phase within 7 days postpartum, illustrating a shorter transition period. Based on these findings, we categorized fecal microbiota succession into three distinct stages: stabilization (FDMM1), transition (FDMM4 and FDMM5), and stabilization (FDMM2 and FDMM3). Using the top 120 ASVs with a random forest algorithm, we developed a classification model, which after rigorous cross-validation showed high predictive accuracy (AUC > 0.95) (Supplementary Fig. S3b and Fig. 4C). We observed that key genera, including *Romboutsia, UCG-005, Paeniclostridium*, and *Bifidobacterium* played pivotal roles in this model, further underscoring their importance in predicting peripartum fecal microbiota succession patterns (Supplementary Fig. S3b).

### Assembly mechanism of rumen and fecal microbes in peripartum dairy cows

**Figure 5:**
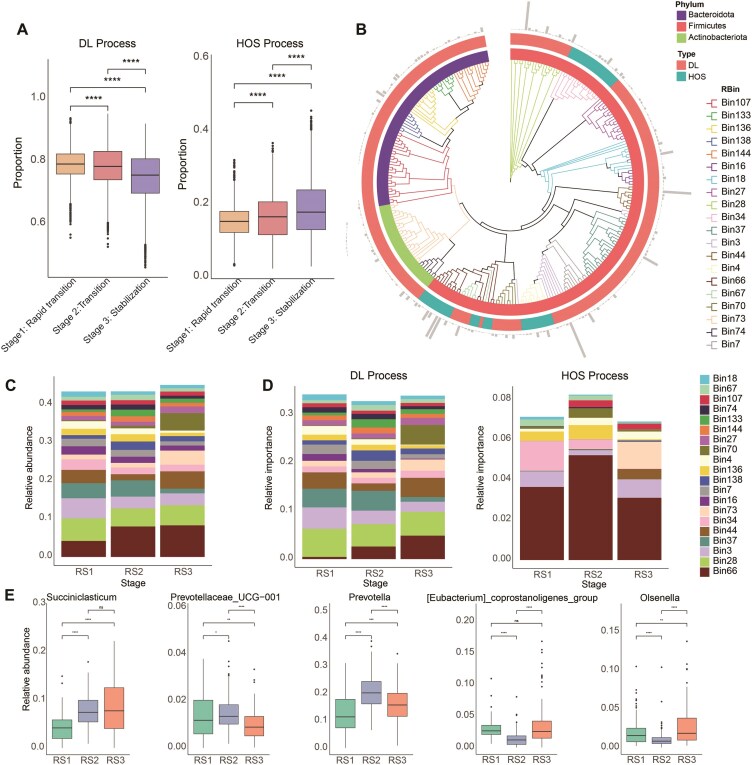
Ecological assembly mechanism of rumen microbiome in peripartum dairy cows. (A) Relative importance of HOS and DL processes in the rumen microbiome at different successional stages in peripartum cows. HOS and DL represent ​“​Homogeneous Selection​​” and ​“​Dispersal Limitation”​, respectively. (B) Differences in ecological processes among different phylogenetic groups (the relative abundance of the top 20 bins) in the rumen. The different colors of the inner and outer circles represent the phylum affiliations of the bins and the ecological processes driven by the bins, respectively. (C) Stacked plot showing the relative abundance of the top 20 bins in different rumen stages. (D) Relative contribution of the top 20 bins to DL and HOS processes in different rumen stages. (E) The relative abundance of representative genera of bins contributing to succession at different rumen stages. RS represents a rumen succession stage.

**Figure 6:**
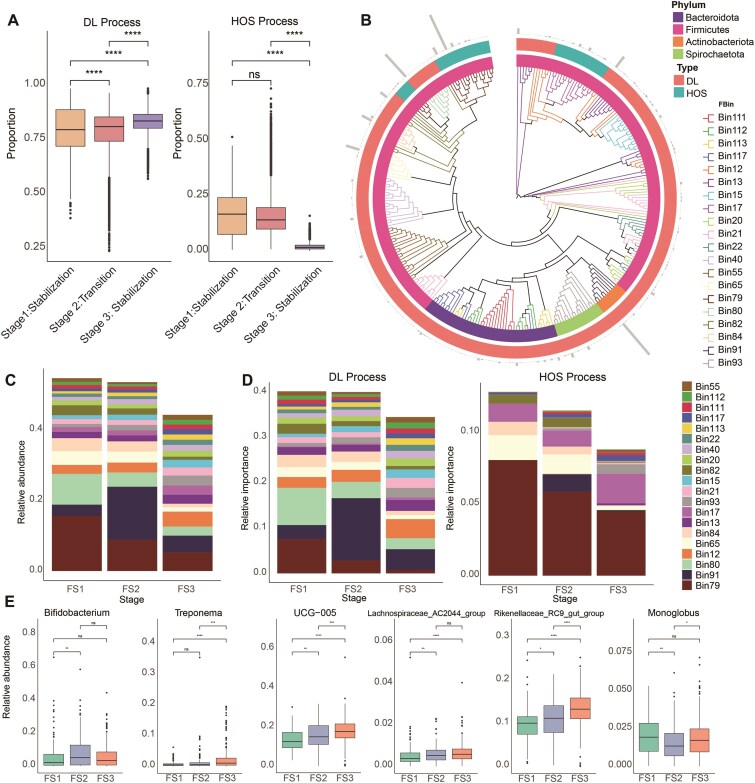
Ecological assembly mechanism of the fecal microbiome in peripartum dairy cows. (A) Relative importance of HOS and DL processes in the fecal microbiome at different successional stages in peripartum cows. HOS and DL represent “​Homogeneous Selection​​” and ​“​Dispersal Limitation”​​, respectively. (B) Differences in ecological processes among different phylogenetic groups (the relative abundance of the top 20 bins) in the feces. The different colors of the inner and outer circles represent the phylum affiliations of the bins and the ecological processes driven by the bins, respectively. (C) Stacked plot showing the relative abundance of the top 20 bins in different fecal DMMs. (D) Relative contribution of the top 20 bins to DL and HOS processes in different fecal DMMs. (E) The relative abundance of representative genera of bins contributing to succession at different fecal stages. FS represents a fecal succession stage.

We demonstrated that microbial succession in the rumen and feces of peripartum dairy cows progresses through three stages. Understanding the underlying reasons for these transformations is of great interest. Therefore, we explored the internal driving forces of rumen and fecal microbiota using phylogenetic bin-based null model analysis (ICAMP) of ecological models to elucidate potential factors influencing microbial community dynamics. Our analysis revealed that stochastic processes dominated the microbial assembly in both rumen (81%) and fecal (82%) communities of peripartum dairy cows (Supplementary Fig. S4a and b). Among these processes, dispersal limitation (DL) emerged as the primary driver in stochastic processes, while homogeneous selection (HOS) constituted the most significant deterministic process (Supplementary Fig. S4a and b). We further compared the ecological processes of different microbial clusters, focusing primarily on HOS and DL because of the minor relative contributions of other processes (heterogeneous selection, homogenizing dispersal, and drift). We found significant differences in the HOS and DL processes among the different ruminal and fecal succession patterns (Figs 5A and 6A), indicating that ecological processes drive the succession of microbial communities in the rumen and feces of peripartum cows. In the rumen, we observed a significant decline in the proportion of DL processes during the succession period, whereas the proportion of HOS processes increased. Conversely, the fecal microbiota exhibited higher DL and lower HOS during succession, highlighting the key role of deterministic and stochastic processes in the succession of ruminal and fecal microbiota during the peripartum period, respectively.

Furthermore, we divided ruminal and fecal ASVs into 144 and 137 bins, respectively (Supplementary Tables S3 and S4). In the rumen, deterministic HOS dominated 3 of the top 20 relative abundances of RBins (bins for rumen microbiota), whereas DL dominated the remaining 17 RBins (Fig. 5B). Conversely, in feces, HOS dominated 2 of the top 20 relative abundances of FBins (bins for fecal microbiota), whereas DL dominated the remaining 18 FBins (Fig. 6B). We also presented the abundance and ecological process contributions of the top 20 bins in the rumen and feces at different successional stages (Figs 5C and 6C). In the rumen, *Succiniclasticum, Prevotellaceae UCG-001, Prevotella*, the *Eubacterium coprostanoligenes group*, and *Olsenella* were identified as key drivers of microbial succession, with their changes in relative abundance across different stages aligning with their contributions to the HOS process (Fig. 5D and E). Similarly, in feces, *Bifidobacterium, Treponema, UCG-005*, the *Lachnospiraceae AC2044 group*, the *Rikenellaceae RC9 gut group*, and *Monoglobus* were identified as key drivers of microbial succession, with their relative abundance changes across stages consistent with their contributions to the DL process (Fig. 6D and E).

### Contribution of multiple individual factors to gut microbial succession

**Figure 7:**
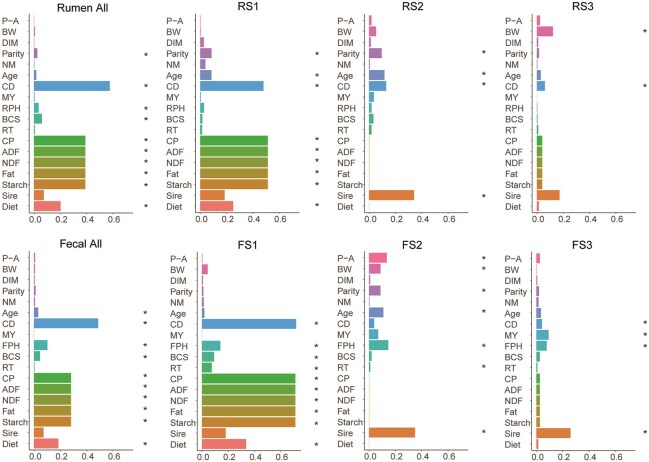
Significance and explained variance of 18 microbiome covariates modelled by EnvFit across all data types. RS represents a rumen succession stage. FS represents a fecal succession stage. Horizontal bars show the amount of variance (*r^2^*) explained by each covariate in the model as determined by EnvFit. Significant covariates (*P* < 0.05) are represented in bold. P-A: predelivery—actual; BW: birth weight; DIM: days in milk; NM: number of matings; CD: calving to days; MY: milk yield; RPH: ruminal pH; BCS: body condition score; RT: rectal temperature; FPH: fecal pH; CP: crude protein; ADF: acid detergent fiber; NDF: neutral detergent fiber.

To assess the influence of individual factors on gut microbial succession, the samples were stratified into all and three successional stages for covariate analysis (Fig. 7). We found that factors such as diet, parity, age, calving to days (CD), and pH were associated with rumen microbial succession. Specifically, CD and dietary nutrient levels explained most of the variance in rumen stage 1 (RS1), CD and birth weight explained most of the variance in RS3, while RS2 was most strongly related to sire. For fecal microbiota, CD, diet, and age were significant factors associated with microbial succession, with CD and diet explaining most of the variance in fecal stage 1 (FS1). FS2 was associated with factors such as sire, pH, and predelivery—actual data, and sire also explained the largest variance in FS3.

Furthermore, we further examined the effects of individual factors on key taxa in the community assembly using the MaAsLin2 method (Supplementary Table S5). In the rumen, the relative abundance of *Succiniclasticum, Prevotella, Prevotellaceae UCG-001, Olsenella*, and the *Eubacterium coprostanoligenes group* was most strongly associated with CD, diet, and pH. In the hindgut, the results show that the relative abundance of the *Lachnospiraceae AC2044 group* was associated with sire and milk yield in last parity, and the relative abundance of *Monoglobus* was related to diet. However, other key taxa were not associated with these individual factors.

### Gut microbial succession types influence host metabolic phenotypes in peripartum dairy cows

**Figure 8:**
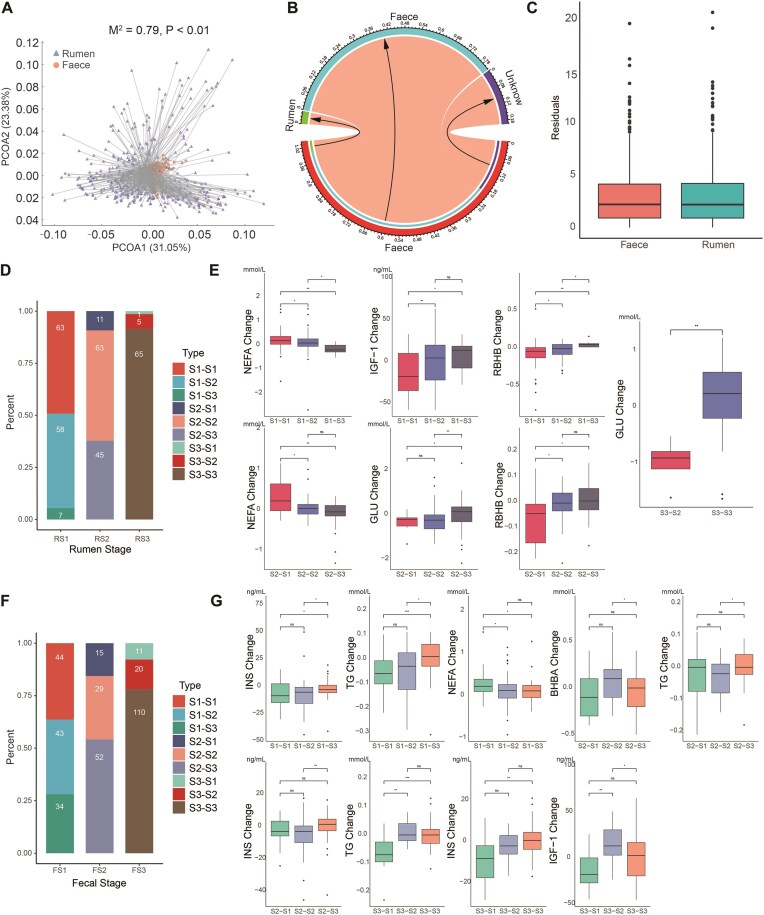
Analysis of the relationship between the rumen and fecal microbiome as well as the effect of succession types on host metabolic phenotype in peripartum cows. (A) Procrustes analysis of the correlation between ruminal and fecal microbiome based on the Bray–Curtis dissimilarity of ASVs (*M*^2^ = 0.79, *P* < 0.01, 999 permutations). (B) Source tracking of the fecal microbiome in peripartum cows. Fecal samples from the former sampling timepoint and rumen samples from the same sampling timepoint were considered potential sources of feces at this timepoint in the same cow. (C) Residuals showing the difference in the microbe–host association from rumen and feces with relative abundance. Microbial succession types and statistics: (D) rumen and (F) feces. Boxplot showing the significance test of effect of succession types on host metabolic phenotype (only significant combinations are shown): (E) rumen and (G) feces. S1–S1: the microbial stage of the individual transitions from S1 to S1; RBHB: revised quantitative insulin sensitivity check index-β-hydroxybutyrate; BHB: β-hydroxybutyrate; HP: haptoglobin; T-AOC: total antioxidant capacity; AST: aspartate aminotransferase; ALT: alanine aminotransferase; IGF-1: insulin-like growth factor 1; NEFA: non-esterified fatty acids; TG: triglycerides; INS: insulin.

First, we assessed the dissimilarity at the ASVs level between the rumen and fecal microbiota, yielding an *M*^2^ value of 0.79 (Fig. 8A). Further source-tracking analysis showed that approximately 80% of the fecal microbiota originated from fecal microbiota at the previous sampling timepoint, whereas only 4% could be traced back to rumen microbiota at the same sampling timepoint (Fig. 8B). These results suggest a weak link between the rumen and fecal microbiota, supporting their consideration as distinct units when studying their effects on the metabolism of peripartum dairy cows. Additionally, the rumen and fecal microbiota contributed to changes in blood metabolic indicators by 20.64% and 19%, respectively (Supplementary Table S6). Concomitantly, we did not observe any significant difference in the residuals between these microbiota and blood indicators, further supporting their similar contributions to the host metabolism (Fig. 8C).

Based on these findings, we separately counted the transformation types of gut microbiota and analyzed effects of the different types on host metabolic phenotypes at the individual level (Fig. 8D and F). Significant differences in blood metabolic indicators were observed across different transformation types (Fig. 8E and G). Transformation types in the rumen microbiota significantly influenced host change levels of serum non-esterified fatty acids (NEFA), insulin-like growth factor-1 (IGF-1), revised quantitative insulin sensitivity check index-β-hydroxybutyrate (RBHB), and glucose (*P* < 0.05), while transformation types in the fecal microbiota notably affected serum insulin (INS), triglycerides (TG), NEFA, β-hydroxybutyrate (BHB), and IGF-1 change levels (*P* < 0.05).

### Transition in the metabolic capacity of the gut microbiome and microbe–host interactions in peripartum dairy cows

**Figure 9:**
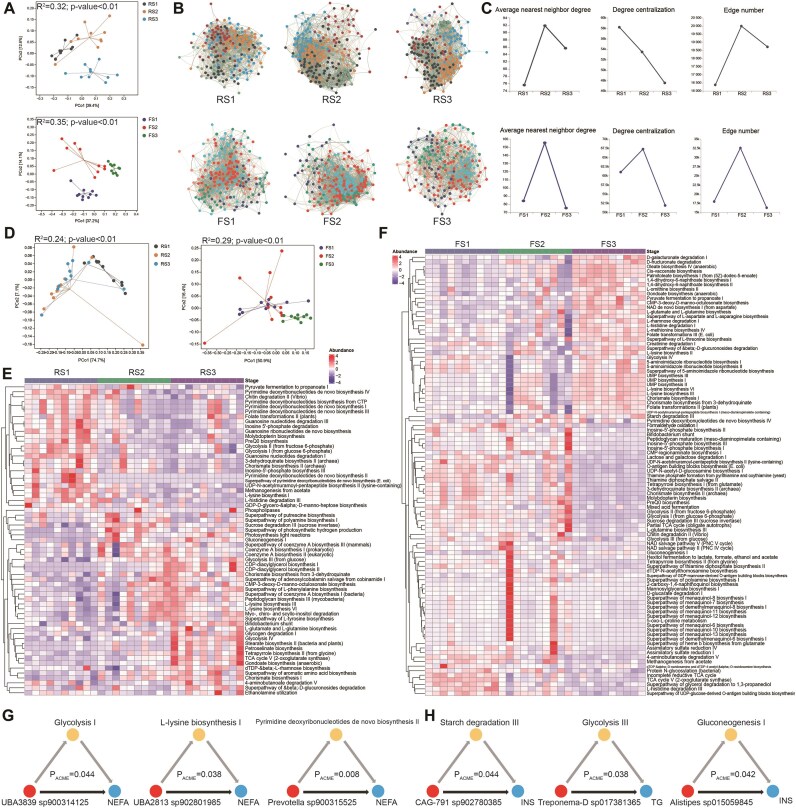
Analysis of microbiome–host interactions in peripartum cows. (A) The PCoA based on species-level Bray–Curtis dissimilarity shows differences in rumen and fecal microbiome profiles across succession stages. (B) The co-occurrence network diagram displays the SparCC interaction relationships of the top 500 abundant genera in different rumen of fecal succession stages (*R* > 0.6; *P* < 0.05). (C) Topological analysis of the co-occurrence network. (D) The PCoA analysis based on Bray–Curtis dissimilarity of pathways reveals distinct microbial functional profiles in the rumen and feces across different successional stages. Heatmap showing the significantly different metabolic pathways (normalized) of the microbiome at different succession stages in peripartum cows: (E) rumen and (F) feces. Part of mediation linkages among the species, pathways, and metabolic phenotype: (E) rumen and (F) feces.

To validate our bacterial findings and evaluate functional succession transitions in rumen and fecal microbiome, we analyzed microbial and functional changes across succession stages using metagenomic data. Rumen and fecal microbiome clustered distinctly at the species level (Fig. 9A), and network analysis also revealed significant stage-specific differences in microbial interactions (Fig. 9B and C). For example, in the stable stages of feces (FS1 and FS3), species showed simpler interactions and lower centrality (Fig. 9C). Meanwhile, the node analysis results also revealed that the key taxa driving microbial succession, such as ruminal *Prevotella* and *Succiniclasticum*, as well as fecal *Bifidobacterium* and *Treponema*, played pivotal roles in the network composition (Supplementary Tables S7–12).

Similarly, rumen and fecal microbiota clustered separately at the pathway level (Fig. 9D). We found that the RS2 and RS3 stages were more similar, while FS2 and FS1 stages were closer. Despite having different dominant species at various successional stages, these results further suggest that the successional patterns of rumen and fecal microbiota during the peripartum period are fundamentally different. In addition, metabolic pathways also displayed stage-specific patterns (Fig. 9E and F). In the rumen, the RS1 stage was characterized by upregulated pathways involved in pyruvate fermentation, glycolysis, nucleotide biosynthesis, and amino acid metabolism. In the RS3 stage, there was an upregulation of pathways related to the Bifidobacterium shunt, glycogen degradation, and aromatic amino acid biosynthesis. Notably, polyamine biosynthesis, gluconeogenesis, and coenzyme A synthesis were consistently upregulated in both RS2 and RS3 stages. In the hindgut, metabolic pathways such as sulfate reduction and sulfur metabolism, methanogenesis V, and the incomplete reductive TCA cycle were upregulated in the FS1 stage. The FS3 stage exhibited upregulation of pathways related to D-galacturonate degradation I, D-fructuronate degradation, glycolysis IV, L-lysine biosynthesis, and UMP biosynthesis. Common pathways, such as inosine-5′-phosphate biosynthesis, glycolysis I and II, mixed acid fermentation, and thiamine metabolism, were upregulated in both FS1 and FS2 stages.

Further, we used mediation models to explore the relationships between significantly different species, metabolic pathways, and metabolic phenotypes. In the rumen, we identified 280 relationships with mediating effects, 19 of which showed mediation, direct, and total effects (Supplementary Table S13). For example, rumen *Prevotella sp900315525* and pyrimidine deoxyribonucleotide de novo biosynthesis II promotes the elevation of serum NEFA levels. In contrast, the elevated abundance of *UBA3839 sp900314125* and glycolysis I as well as *UBA2813 sp902801985* and L-lysine biosynthesis I reduce serum NEFA levels (Fig. 9G). In the hindgut, we identified 1,360 relationships with mediating effects, 41 of which showed mediation, direct, and total effects (Supplementary Table S14). For example, the abundance of *CAG-791 sp902780385* and starch degradation III was associated with increased serum INS levels. Conversely, the increased abundance of *Treponema-D sp017381365* and glycolysis III as well as *Alistipes sp015059845* and gluconeogenesis I reduce serum TG and INS levels (Fig. 9H).

## Discussion

To our knowledge, this study is the first to investigate the gut microbiomes of peripartum dairy cows using a large-scale, high-frequency sampling approach. We also uniquely employed a combination of microbial clustering, ecological modeling, and random forest analysis to reveal that the rumen and fecal microbiome succession in peripartum dairy cows can be divided into three distinct stages. The results showed significant differences in the composition and function of rumen and fecal microbiota across these successional stages. Moreover, the transitions between these stages were driven not only by different taxa but also significantly influenced the metabolic phenotype changes in peripartum dairy cows.

As expected, we observed significant changes in the microbial structure of the rumen and feces in peripartum dairy cows. However, compared to previous studies, the trends in the Chao1 and Shannon indices of the rumen and fecal microbiomes in our study showed unique characteristics [23–26]. Notably, these previous studies were conducted on the same breed of cows with similar peripartum diets—high-fiber diets before calving and high-starch diets postpartum. This underscores the importance of large sample sizes and high temporal resolution in revealing changes in gut microbiome. Furthermore, we found that individual differences had an even greater impact on gut microbiome structure throughout the peripartum period than changes observed within individuals over time, indicating that peripartum dairy cow trials need larger sample sizes to mitigate the impact of interindividual variability on experimental outcomes, thereby effectively controlling the occurrence of false positive errors. Importantly, we identified three deterministic successional stages in both the rumen (rapid transition, transition, stabilization) and feces (stabilization, transition, stabilization), revealing the compartmentalized adaptive strategies of gut microbial communities to peripartum stress. Rumen microbiota were more sensitive to stress, while fecal microbiota exhibited a delayed response, a characteristic also observed in a study by Bach et al. [24]. Typically, the most severe negative energy balance in dairy cows occurs within the first 2 weeks after calving and gradually alleviates as the days in milk increasing. This aligns with the stabilization of the ruminal microbiota beginning at 14 days postpartum, indicating that the microbial community restructuring is an active participant in metabolic recovery, not just a bystander. During the ruminal stabilization stage, the dominant *Succiniclasticum* is associated with the conversion of succinate to propionate, a critical pathway for gluconeogenesis during negative energy balance. In contrast, the early stabilization of the feces (at 10 days postpartum) coincides with the peak incidence of metabolic diseases [5, 29], suggesting that the fecal microbiota may prioritize immune homeostasis over energy acquisition [30].

Additionally, we innovatively used ecological modeling to estimate the assembly processes of rumen and fecal microbiota in dairy cows. The divergent assembly mechanisms of the rumen microbiota (dominated by HOS) and the fecal microbiota (dominated by DL) during succession highlight the compartment-specific selection pressures. HOS suggests that under environmental pressure, microbial community compositions tend to become homogeneous, emphasizing the dominant role of environmental factors in shaping microbial community structures [31, 32]. In contrast, DL refers to changes in the relative abundance of species in the microbial community due to random events (including random deaths or births), implying that ecological niches in the community are not completely occupied or utilized [33, 34]. *Succiniclasticum* is not only the dominant genus during the rumen stabilization stage but also actively responds to the HOS process within the rumen microbial community. Previous studies have already confirmed the impact of diet on community succession in dairy cows [16]. Combined with the linear increase in feed intake of postpartum cows [35], this may reflect the selective pressure imposed by the host through pH shifts and the influx of dietary starch, which also favors the growth of acid-tolerant taxa such as *Prevotella*. In contrast, the DL-driven pattern in the feces indicates that neutral processes dominate, likely due to physical niche partitioning (e.g., mucus layer gradients) which limits microbial dispersal [36]. Notably, *Bifidobacterium*, as a key DL-associated taxon, may occupy unutilized niches by producing substances such as acetate, which helps maintain gut homeostasis and inhibit pathogens [37–40]. These findings align with metacommunity theory, according to which rumen microbiota resemble a “species-sorting” model, while fecal communities follow “neutral assembly.” These findings further support the results of Shen et al. [41], which suggested that the fecal microbiome in ruminants more accurately reflects the host health status compared to the rumen microbiome.

Our covariate analysis revealed that gut microbial succession is shaped by a complex interplay of temporal, nutritional, and host-specific factors, with distinct drivers dominating different successional stages. Notably, sire effect dominates in RS2, which may imply that the assembly process of the peripartum rumen microbiota is influenced by genetic factors. Parallel patterns were observed in fecal microbiota, where CD and diet drove early succession (FS1), while sire effect predominated in later stages (FS3), reinforcing the temporal hierarchy of environmental versus host-intrinsic influences across gut niches. The MaAsLin2 analysis further highlighted functional linkages between key taxa (e.g., *Succiniclasticum, Prevotella*) and critical parameters such as CD and pH, suggesting these microbes may serve as biological integrators of postpartum physiological changes and dietary adaptation. Importantly, the decoupled nature of rumen and fecal microbial ecosystems underscores their compartmentalized contributions to host metabolism. Despite minimal direct microbial exchange, both ecosystems exerted comparable influence on blood metabolic profiles (∼20% variance) through distinct pathways. Rumen microbial transformation types preferentially modulated energy mobilization markers (NEFA, GLU) and growth factors (IGF-1), aligning with its role as the primary nutrient-processing organ. Conversely, fecal microbiota dynamics showed stronger associations with lipid metabolism regulators (TG, BHBA) and INS signaling, potentially reflecting downstream metabolic byproduct processing or gut barrier function interactions. These compartment-specific metabolic fingerprints emphasize the need for ecosystem-targeted interventions during the transition period. The stage-specific dominance of sire-related effect across both gut sites raises intriguing questions about heritable microbial transmission mechanisms and their potential for selective breeding strategies. Previous research on humans, pigs, sheep, and beef cattle has demonstrated significant correlations between host SNPs and their gut microbiome [42–45], underscoring the potential of genetic breeding for altering the gut microbiota composition of peripartum dairy cows to reduce the incidence of metabolic diseases. However, the limited explanatory power of measured factors for certain key taxa suggests unaccounted variables, possibly including microbial cross-talk or host epigenetic regulation, warranting further investigation.

We further revealed fundamental differences in the functional trajectories and metabolic specialization of the peripartum rumen and fecal microbiomes through metagenomic and network analyses, emphasizing their compartmentalized yet coordinated roles in shaping host metabolic adaptation. The rumen microbial communities prioritize nutrient extraction and energy flux (e.g., pyruvate fermentation and glycolysis in RS1; the Bifidobacterium shunt in RS3), while the fecal microbiota specialize in substrate salvage and metabolic byproduct processing (e.g., sulfate reduction in FS1; D-galacturonate degradation in FS3). These observations are consistent with the anatomical and physiological roles of each gut compartment: the rumen rapidly adapts to postpartum dietary shifts and lactation-driven energy demands, while the feces fine-tunes downstream metabolic pathways to manage residual substrates and systemic metabolic stress. The stage-specific complexity of microbial networks further supports the dichotomy: simpler interactions in the stable phases of the fecal microbiota suggest a homeostatic “maintenance” state, while the dynamic succession stages of the rumen exhibit enriched pathways for polyamine and coenzyme A synthesis, likely reflecting their roles in sustaining microbial turnover and redox balance during metabolic upheaval. Notably, key taxa such as *Prevotella* and *Succiniclasticum* in the rumen, and *Bifidobacterium* and *Treponema* in the feces, emerge as central network nodes, acting as linchpins that connect microbial community structure with metabolic output. Their prominence in energy-yielding processes (glycolysis, mixed-acid fermentation) and biosynthesis (nucleotides, amino acids) positions these taxa as critical regulators of host–microbe metabolic crosstalk. The mediation models further demonstrate the potential mechanisms by which key microbes regulate host metabolic phenotypes. For instance, the glucose-related pathway in both the rumen and feces mediates the impact of key microbes on host metabolism, highlighting the crucial role of glucose supplementation in postpartum diets for maintaining dairy cow health. Previous studies have linked *Alistipes* to short-chain fatty acid (SCFA) production, including acetate and propionate, and its reduced abundance is associated with disease progression, including non-alcoholic fatty liver disease and non-alcoholic steatohepatitis, due to decreased SCFA levels [46, 47]. As is well known, postpartum dairy cows are susceptible to fatty liver. Thus, *Alistipes sp015059845* in the feces may play a vital role in maintaining liver and intestinal health in postpartum dairy cows. Additionally, the elevated abundance of *UBA3839 sp900314125* associated with glycolysis I and *UBA2813 sp902801985* associated with L-lysine biosynthesis I in the rumen mediated the reduction of serum NEFA levels, indicating complex interplay between microbial metabolism and host energy regulation in peripartum dairy cows. *UBA3839 sp900314125*, through its association with glycolysis I, may promote the production of propionate, a key precursor for gluconeogenesis [48]. Increased availability of propionate enhances hepatic glucose synthesis, thereby raising blood glucose levels and subsequently boosting INS signaling. This cascade inhibits lipolysis in adipose tissue by suppressing hormone-sensitive lipase, thereby reducing the release of NEFA into the bloodstream [49]. Meanwhile, *UBA2813 sp902801985* associated with L-lysine biosynthesis I may optimize nitrogen utilization and energy efficiency within the gut microbiota. As an essential amino acid, lysine not only supports protein synthesis but may also modulate host metabolic pathways such as AMPK or mTOR signaling to enhance glucose uptake and utilization, further reducing reliance on fat mobilization [50]. Overall, we propose that modulating the fecal microbiota may be more beneficial in preventing postpartum metabolic diseases in dairy cows because its transition period precedes the typical onset of such conditions. These results enhance our understanding of the spatiotemporal coordination of gut microbial ecosystems during metabolic stress in peripartum dairy cows and provide a framework for developing targeted management strategies to optimize cow health and productivity during critical transition periods.

This study is the first to comprehensively provide a dynamic perspective on the gut microbiome of peripartum dairy cows, enhancing our understanding of the dynamic interactions between the gut microbiome and the host during the peripartum period. Because this study only examined healthy cows, our next research objective is to compare the microbiomes of healthy and diseased cows to explore mechanisms related to postpartum metabolic disorders. Additionally, this study lacks information on feed intake, which is a critical factor influencing gut microbiome composition. In future research, we plan to investigate the impact of feed intake on the dynamic changes in the gut microbiome of peripartum dairy cows. Moreover, based on the patterns observed in this study, further research is needed to verify whether modifying the succession process of the gut microbiome by targeting the identified key microbes and pathways can effectively regulate host metabolism in peripartum cows, thereby preventing postpartum metabolic disorders.

## Materials and Methods

### Animals, experimental design, and sample collection

This study was conducted at a commercial dairy farm in Shanxi Province, China. The dairy cows involved in the experiment were managed using a traditional feeding model. Two months before the expected calving date, the cows were moved to a dry cow barn and started on a high-fiber total mixed ration (TMR) (dry period) diet. After calving, the cows were immediately separated from their calves to prevent further exposure of the dams to microbes from the calves. The cows were then moved to a transition barn where professional technicians immediately milked the colostrum and fed the animals a fresh cow diet (high-starch TMR). Three days after calving, the cows were transferred to a fresh barn until the end of the experiment. In the fresh barn, the cows were allowed access to the same high-starch TMR and water ad libitum during the experiment. Details on TMR are provided in Supplementary Table S15. Postpartum care was given in the transition barn within 1–2 days, which included feeding an oral bolus (Bovikalc bolus, Boehringer Ingelheim, St Joseph, MO, USA) containing CaCl_2_ and CaSO_4_ (43 g of calcium), measuring rectal temperature (M900 Thermometer, GLA Agricultural Electronics, Inc., San Luis Obispo, CA, USA), and drenching 300 ml liquid propylene glycol orally using a drench gun.

The study included 211 healthy, multiparous, pregnant dairy cows. During the experiment, an experienced veterinarian conducted health assessments, including evaluations of body condition, rectal temperature, blood BHB concentration, and mental state. Supplementary Table S16 presents the health assessment criteria. After excluding cows with abnormal conditions and those treated with medication during the experimental period to avoid interference with the generalizability of our results, 91 normal peripartum cows were included (Fig. 1). The strict control of normal cows allowed us to minimize interference from other macro factors, providing a clearer view of the natural dynamic changes in the gut microbiome and key factors driving these changes.

Rumen fluid, fecal, and blood samples were collected from these cows 21 days before calving (expected calving date) and on days 1, 3, 7, 14, and 21 after calving (Fig. 1). The means and standard deviations (SDs) of the actual sampling day on day −21 were day −19.29 ± 4.70 (min–max, −31 to −6). Regardless of the exact hour of the calving day, samples of day 1 were collected on the day following parturition. All samples were collected before the morning feeding on the designated sampling day. Rumen fluid samples were collected using a special rumen tube (Anscitech Animal Husbandry Technology Co., Ltd., Wuhan, China) designed based on the physiological structure of adult cows to ensure that the tube reached the ventral aspect of the rumen. The exterior metal of the rumen tube was polished to minimize damage to the esophagus and rumen. Fecal samples were obtained from the rectum of cows by research personnel wearing sterile long-arm gloves. Blood samples were collected from the caudal veins of cows in 10 ml vacuum blood collection tubes (Hua Xia Heng Yuan Technolongy Co., Ltd, Beijing, China). Rumen fluid and fecal samples were immediately transferred to 2 ml cryogenic tubes and stored in liquid nitrogen until subsequent bacterial diversity analysis. Blood samples were centrifuged at 4,000*g* and 4°C for 15 min to obtain the serum, which was then transferred to 2 ml cryogenic tubes and stored in liquid nitrogen for subsequent analysis of energy metabolism, liver function, and antioxidant indicators.

### Serum parameter measurement

GLU, TG, aspartate aminotransferase (AST), and alanine aminotransferase (ALT) concentrations in dairy serum were determined using an automatic biochemistry analyzer (GF-D200, Analytical Instrument Co. Ltd., Gaomi, China). INS levels were quantified via radioimmunoassays using a multitube counter (BFM-96; Zhongcheng Technology, Hefei, China). Direct field assessment of BHB levels was performed in freshly collected blood samples using specific portable test strips (Nova Vet; Nova Biomedical Corporation, Waltham, MA, USA). Serum concentrations of NEFA (catalog no. A042-2-1), IGF-1 (catalog no. H041-1-2), total antioxidant capacity (T-AOC; catalog no. A015-1-2), and haptoglobin (HP; catalog no. H136) were quantified using commercial enzyme-linked immunosorbent assay (ELISA) kits (Nanjing Jiancheng Bioengineering Institute, Nanjing, China). Briefly, following the manufacturer’s protocol, serum samples were thoroughly mixed with the reagents provided in the kit, incubated at 37°C, and then analyzed using a microplate reader to measure absorbance values, with target concentrations calculated based on the standard curve. The RBHB index, calculated as 1/[log glucose (mg/dl) + log INS (μU/ml) + log NEFA (mmol/l) + log BHB (mmol/l)], was used to evaluate INS resistance in dairy cows based on a previously described method [51].

### Microbial DNA extraction

Total genomic DNA was extracted from all samples using a DNeasy PowerSoil Pro Kit 47014 (Qiagen, Hilden, Germany), according to the manufacturer’s instructions, and stored at −20°C for subsequent analysis. DNA quantity and quality were assessed using a NanoDrop NC2000 spectrophotometer (RRID:SCR_018042, Thermo Fisher Scientific, Waltham, MA, USA) and agarose gel electrophoresis, respectively.

### 16S rRNA gene sequencing

The 16S rRNA gene was amplified using universal primers (338F: 5′-ACTCCTACGGGAGGCAGCA-3′; 806R: 5′-GGACTACHVGGGTWTCTAAT-3′) targeting the V3–V4 region, with 7 bp barcodes added for multiplex sequencing. Each PCR mixture contained 5 μl buffer (5×), 0.25 μl Fast pfu DNA Polymerase (5 U/μl), 2 μl (2.5 mM) dNTPs, 1 μl (10 μM) of each forward and reverse primer, 1 μl DNA template, and 14.75 μl double-distilled H_2_O. Thermal cycling steps involved an initial denaturation step at 98°C for 5 min, followed by 25 cycles of denaturation at 98°C for 30 s, annealing at 53°C for 30 s, and extension at 72°C for 45 s, with a final extension at 72°C for 5 min. The PCR amplicons were purified using Vazyme VAHTSTM DNA Clean Beads (Vazyme, Nanjing, China) and quantified using the Quant-iT PicoGreen dsDNA Assay Kit (Invitrogen, Carlsbad, CA, USA). After each quantification step, the amplicons were pooled in equal amounts, and paired-end 2 × 250 bp sequencing was performed using the Illumina NovaSeq platform with the NovaSeq 6000 SP Reagent Kit (500 cycles) at Shanghai Personal Biotechnology Co., Ltd (Shanghai, China).

Data quality control and analyses were performed using the QIIME2 pipeline (RRID:SCR_021258) with slight modifications according to official tutorials [52]. Briefly, raw sequence data were demultiplexed using the demux plugin, followed by primer cutting using the cutadapt plugin [53]. The sequences were then quality filtered, denoised, merged, and chimeras were removed using the DADA2 plugin (RRID:SCR_023519) [54]. Non-singleton ASVs were aligned using mafft, and a phylogeny tree was constructed using fasttree2 [55, 56]. ASVs were taxonomically classified using the classify-sklearn naive Bayes taxonomy classifier in the feature-classifier plugin against the SILVA Release 138.1 database [57].

### Metagenomic sequencing

We conducted metagenomic analyses on samples randomly selected from the ruminal and fecal collections at three successional stages (10 samples per stage, total *N* = 60). The extracted total DNA was processed using the Illumina TruSeq Nano DNA LT Library Preparation Kit (Illumina, San Diego, CA, USA) to construct metagenomic shotgun sequencing libraries with an insert length of approximately 400 bp. Each library was sequenced on the Illumina NovaSeq X Plus platform (RRID:SCR_024568, Illumina, San Diego, CA, USA) and Personal Biotechnology Co., Ltd (Shanghai, China) using the PE150 strategy. For metagenomic data processing, Cutadapt (v.1.2.1; RRID:SCR_011841)was used to remove sequencing adapters from the raw reads [58]. Low-quality reads were trimmed using a sliding window algorithm in fastp (v.0.23.2) [59]. Reads were aligned to the bovine genome using Minimap2 (v.2.24-f1122, RRID:SCR_018550) to remove host contamination [60]. Subsequently, Kaiju (v.1.9.0) was used to classify metagenomic reads against the GTDB-derived database (v.207) for each sample [61]. Reads assigned to Metazoa or Viridiplantae were excluded from downstream analysis. Megahit (v.1.1.2, RRID:SCR_018551) was used with the “-k-list 33,55,77,99,127 -min-contig-len 300” to assemble reads in each sample [62]. Contigs generated were clustered using the “easy-linclust” mode of MMseqs2 (v.15, RRID:SCR_022962) with a sequence identity threshold of 0.95 and a 90% coverage of the shorter contigs [63]. Genes were predicted using Prodigal (v.2.6.3, RRID:SCR_011936) [64]. The CDS sequences from all samples were clustered using the “easy-cluster” mode of Mmseqs2, with a protein sequence identity threshold of 0.95 and 90% coverage of shorter sequences. Reads were then mapped to the predicted gene sequences using Minimap2, and featureCounts was used to calculate the number of reads aligned to each gene [65]. Abundance was expressed in TPM (transcripts per million). The functional annotation of non-redundant genes was performed using the “search” mode of Mmseqs2 against the Metacyc database (RRID:SCR_007778) [66].

### Bioinformatics and statistical analysis

After obtaining the ASV datasets of the rumen and fecal microbiome, they were processed using the “phyloseq” package in R (v.4.2.2), with a rarefaction depth set to the minimum sample sequence quantity. The ASV datasets were independently subsetted for individual analysis of the rumen and fecal microbiome. Conversely, for integrated analysis of the rumen and fecal microbiome, combined ASV datasets were aggregated. The downstream analysis of 16S sequencing data mainly includes: individual variation, DMM, α-diversity, β-diversity, LEfSe, Markov chain, random forest, ICAMP, EnvFit, MaAsLin2, Procrustes, and source tracking analysis. For examining the α-diversity, ASV-level indices, including Chao1 richness and the Shannon diversity index, were calculated using the “vegan” package in R [67, 68]. β-Diversity was explored using Bray–Curtis dissimilarity metrics to understand the structural variations in microbial communities across samples using the “vegan” package in R [69]. A DMM model was applied at the genus level to cluster samples based on the microbial community structure [14, 70], with clusters determined according to the lowest Laplace approximation score [70]. This analysis was performed independently for rumen (RDMM) and fecal samples (FDMM). Linear discriminant analysis effect size (LEfSe, LDA > 4, *P* < 0.05) was used to identify the dominant taxa across the groups [71]. Transition dynamics between these states were analyzed via Markov chain models using the Markov chain packages in R [72], following the methodology by Xiao et al. [15]. Models predicting microbial stages of the rumen and fecal microbiome were constructed using random forest algorithms with ASVs that showed >0.1% relative abundance, using the “randomForest” package in R [73]. To reduce model overfitting, five 10-fold cross-validations were conducted using 70% of samples for model building and 30% as the test set. The area under the receiver operating characteristic curve (AUC) was calculated using R. ICAMP was performed using a galaxy-based pipeline to assess the relative importance of deterministic and stochastic processes in bacterial community assembly [74]. The observed taxa were first grouped into bins (“boxes”) based on their phylogenetic relationships, with the minimum number of taxa per bin as the default setting (bin.size.limit=24). Although the main function within icamp.big was used to calculate the within-bin β-nearest taxon index (βNTI), the modified Raup–Crick metric (RC) was used to evaluate the relative importance of different ecological processes within each bin. For each bin, pairwise comparisons with βNRI < −1.96 are considered to be controlled by homogeneous selection, while those with βNRI > +1.96 are controlled by heterogeneous selection. Next, other processes are categorized using the phylogenetic diversity metric RC with |βNRI| ≤ 1.96. When RC < −0.95, it is considered as a process of homogeneous dispersal, while RC > +0.95 indicates a process of dispersal limitation. Cases with |βNRI| ≤ 1.96 and |RC| ≤ 0.95 represent the influence of processes such as drift. EnvFit analysis was conducted to examine the relationship between individual factors (eg., diet, sire, birth weight, predelivery—actual days) and microbial succession stages, using the “vegan” package in R. Correlational analyses of individual factors and genera, with a cow as a random effect and false discovery rate (FDR) adjustment using the Benjamini–Hochberg method, were performed using microbiome multivariate association with linear models (MaAsLin2) [75]. Due to data loss in the commercial dairy farm's herd management system, the records of two cows were irretrievably lost. Consequently, we performed correlation analyses using metadata from the remaining 89 cows. Procrustes analysis between the rumen and fecal ASVs was conducted using the “protest” function in the R package vegan [76]. To further determine the relationship between rumen and fecal microbiomes, fecal microbiome source tracking was conducted using the sourcetracker2 plugin [77], which can assess the proportion of fecal microbiome originating from rumen microbiome at the same timepoint and fecal microbiome at previous timepoints. Residuals between rumen or fecal samples and blood indicators were generated using the “Procrustes” function in the R package vegan [17].

Species analysis of metagenomic sequencing data is based on read count data, while functional analysis is based on TPM datasets. The downstream analyses mainly include: β-diversity of species and microbial functions, co-occurrence network analysis of species, network analysis, differential analysis, and mediation analysis. β-Diversity at the species level, including bacteria, eukaryotes, and archaea, was assessed using Bray–Curtis dissimilarity. The co-occurrence networks were constructed based on SparCC correlation coefficients (|*R*| > 0.6, *P* < 0.05) using the top 500 most abundant genera [78]. Co-occurrence networks and node topology were evaluated to examine interspecies interactions and network centrality [79]. Functional β-diversity was assessed at the pathway level of the Metacyc database using Bray–Curtis dissimilarity. For differential analysis of species, taxa with a prevalence >50% and relative abundance >0.1% were selected. For differential analysis of pathways, taxa with a prevalence >50% and relative abundance >0.01% were selected. Mediation analysis was used to determine whether the effects of species on host metabolic indicators are mediated by microbial functions, using the “mediation” package in R.

β-Diversity was evaluated using PERMANOVA with 999 permutations and visualized using principal coordinate analysis (PCoA) [80]. Genus diversity under different RDMM or FDMM conditions is represented via heatmaps using the ComplexHeatmap package in R [81]. Sample distributions at each peripartum timepoint and succession patterns for RDMM or FDMM are depicted using pie charts and Sankey diagrams [82], respectively. The Markov chain model was visualized using the igraph packages in R [72]. Intraindividual and interindividual compositional variabilities were calculated according to the method described by Olsson et al. [83]. Intraindividual compositional variability was defined as the median Bray–Curtis dissimilarity calculated between samples from a cow (i.e., 20 dissimilarity values were calculated for the 6 samples obtained from each cow). Interindividual compositional variability was defined as the median Bray–Curtis dissimilarity calculated for 6 samples from a cow against all other samples. Additional visualizations were created using ggplot2 in R. All differential analyses were performed using Kruskal–Wallis followed by Dunn’s post-hoc tests using the “Kruskal.test” and “dunn.test” functions in R (dunn.test package). Statistical significance was set at *P* < 0.05.

## Availability of Source Code and Requirements

Not applicable.

## Supplementary Material

giaf042_Supplemental_Files

giaf042_Authors_Response_To_Reviewer_Comments_original_submission

giaf042_GIGA-D-24-00404_original_submission

giaf042_GIGA-D-24-00404_Revision_1

giaf042_Reviewer_1_Report_original_submission Mst.Sogra Banu Juli -- 11/14/2025

giaf042_Reviewer_2_Report_original_submission Shengru Wu -- 2/23/2025

giaf042_Reviewer_2_Report_Revision_1 Shengru Wu -- 3/5/2025

## Data Availability

The metagenomic and 16S rRNA sequencing supporting this work have been archived in the NCBI database under accession numbers PRJNA1161368 and PRJNA1126601, respectively. Other data and files further supporting this work are openly available in the *GigaScience* repository, GigaDB [84].

## Additional Files

**Fig. S1**. Dynamics and diversity of rumen microbiota during the peripartum period. (a) PCoA plot-based Bray–Curtis dissimilarity showing rumen microbiota profiles across different sampling timepoints. (b) Dynamic changes in the α-diversity of rumen microbiota at different periods during the perinatal period. (c) Plots showing a comparison of the 5 bacterial phyla with the highest relative abundance in the rumens of perinatal cows. (d) Laplace approximation scores for ruminal DMM. (e) The top 20 taxa that contributed the most to the accuracy of the rumen DMM are shown in order of importance. (f) Heatmap showing the relative abundance of the top 20 taxa (normalized in each taxon) in each rumen sample.

**Fig. S2**. Dynamics and diversity of fecal microbiota during the peripartum period. (a) PCoA plot-based Bray–Curtis dissimilarity showing fecal microbiota profiles across different sampling timepoints. (b) Dynamic changes in the α-diversity of fecal microbiota at different timepoints during the perinatal period. (c) Plots showing a comparison of the 5 bacterial phyla with the highest relative abundance in the fecal microbiota of perinatal cows. (d) Laplace approximation scores for fecal DMM. (e) The top 20 taxa that contributed the most to the accuracy of the fecal DMM are shown in order of importance. (f) Heatmap showing the relative abundance of the top 20 taxa (normalized in all taxa) in each fecal sample.

**Fig. S3**. Cross-validation of ASVs contributing to the random forest model. (a) Rumen. (b) Feces. ASV: Amplicon sequence variant.

**Fig. S4**. Relative importance of different ecological processes in the microbiota composition of peripartum dairy cows. (a) Rumen. (b) Feces.

**Table S1**. Details of transformation of ruminal DMMs at different timepoints.

**Table S2**. Details of transformation of fecal DMMs at different timepoints.

**Table S3**. Details of ruminal bins in ICAMP assembly.

**Table S4**. Details of fecal bins in ICAMP assembly.

**Table S5**. The relationship between individual factors and key genera driving succession in dairy cows investigated using MaAsLin2.

**Table S6**. Analysis of the contribution of rumen and fecal microbiota to blood metabolism.

**Table S7**. Node analysis of co-occurrence network in RS1 stage.

**Table S8**. Node analysis of co-occurrence network in RS2 stage.

**Table S9**. Node analysis of co-occurrence network in RS3 stage.

**Table S10**. Node analysis of co-occurrence network in FS1 stage.

**Table S11**. Node analysis of co-occurrence network in FS2 stage.

**Table S12**. Node analysis of co-occurrence network in FS3 stage.

**Table S13**. Mediation analysis of ruminal different species, ruminal different pathways, and metabolic phenotype.

**Table S14**. Mediation analysis of fecal different species, fecal different pathways, and metabolic phenotype.

**Table S15**. Feed ingredients and nutrient composition of diets.

**Table S16**. Summary of reasons for elimination.

**Table S17**. The rumen samples corresponding to each cow.

**Table S18**. The fecal samples corresponding to each cow.

## Abbreviations

ALT: alanine aminotransferase; AST: aspartate aminotransferase; ASV: amplicon sequence variant; BHB: β-hydroxybutyrate; DL: dispersal limitation; DMM: Dirichlet multinomial mixture; ELISA: enzyme-linked immunosorbent assay; FDMM: DMM clusters for fecal microbiota; FDR: false discovery rate; GLU: glucose; HOS: homogeneous selection; HP: haptoglobin; ICAMP: phylogenetic bin-based null model analysis; IGF-1: insulin-like growth factor 1; INS: insulin; LEfSe: linear discriminant analysis effect size; modified RC: Raup–Crick metric; NEFA: non-esterified fatty acids; PERMANOVA: permutational multivariate analysis of variance; RBHB: revised quantitative insulin sensitivity check index-β-hydroxybutyrate; RDMM: DMM clusters for rumen microbiota; T-AOC: total antioxidant capacity; TG: triglycerides; TMR: total mixed ration; βNTI: β-nearest taxon index.
