# Supplementary material for: Deterministic succession patterns in the rumen and fecal microbiome associate with host metabolic shifts in peripartum dairy cattle
Source: Gigascience. 2025 May 19;14:giaf042. doi: 10.1093/gigascience/giaf042 (PMC12087452; doi:10.1093/gigascience/giaf042)

**Deterministic Succession Patterns in the Rumen and Fecal Microbiome Associate with host Metabolic Shifts in Periparturient Dairy Cattle**

Shuo Wang^†^, Fanlin Kong^†^, Dongwen Dai, Chen Li, Yangyi Hao, Erdan Wang, Zhijun Cao, Yajing Wang, Wei Wang*, and Shengli Li*

State Key Laboratory of Animal Nutrition and Feeding, Department of Animal Nutrition and Feed Science, College of Animal Science and Technology, China Agricultural University, Beijing 100193, China

***Correspondence:**

Shengli Li: [lishengli@cau.edu.cn](mailto:lishengli@cau.edu.cn); Tel and fax: +86-010-62731254;

Wei Wang: [wei.wang@cau.edu.cn](mailto:wei.wang@cau.edu.cn); Tel and fax: +86-010-62733789

^†^These authors contributed equally: Shuo Wang and Fanlin Kong

**Figs. S1 Dynamics and diversity of rumen microbiota during the periparturient period.** (a) PCoA plot-based Bray–Curtis dissimilarity showing rumen microbiota profiles across different sampling time points. (b) Dynamic changes in the α-diversity of rumen microbiota at different periods during the perinatal period. (c) Plots showing a comparison of the five bacterial phyla with the highest relative abundance in the rumens of perinatal cows. (d) Laplace approximation scores for ruminal DMM. (e) The top 20 taxa that contributed the most to the accuracy of the rumen DMM are shown in the order of importance. (f) Heatmap showing the relative abundance of the top 20 taxa (normalised in each taxon) in each rumen sample.


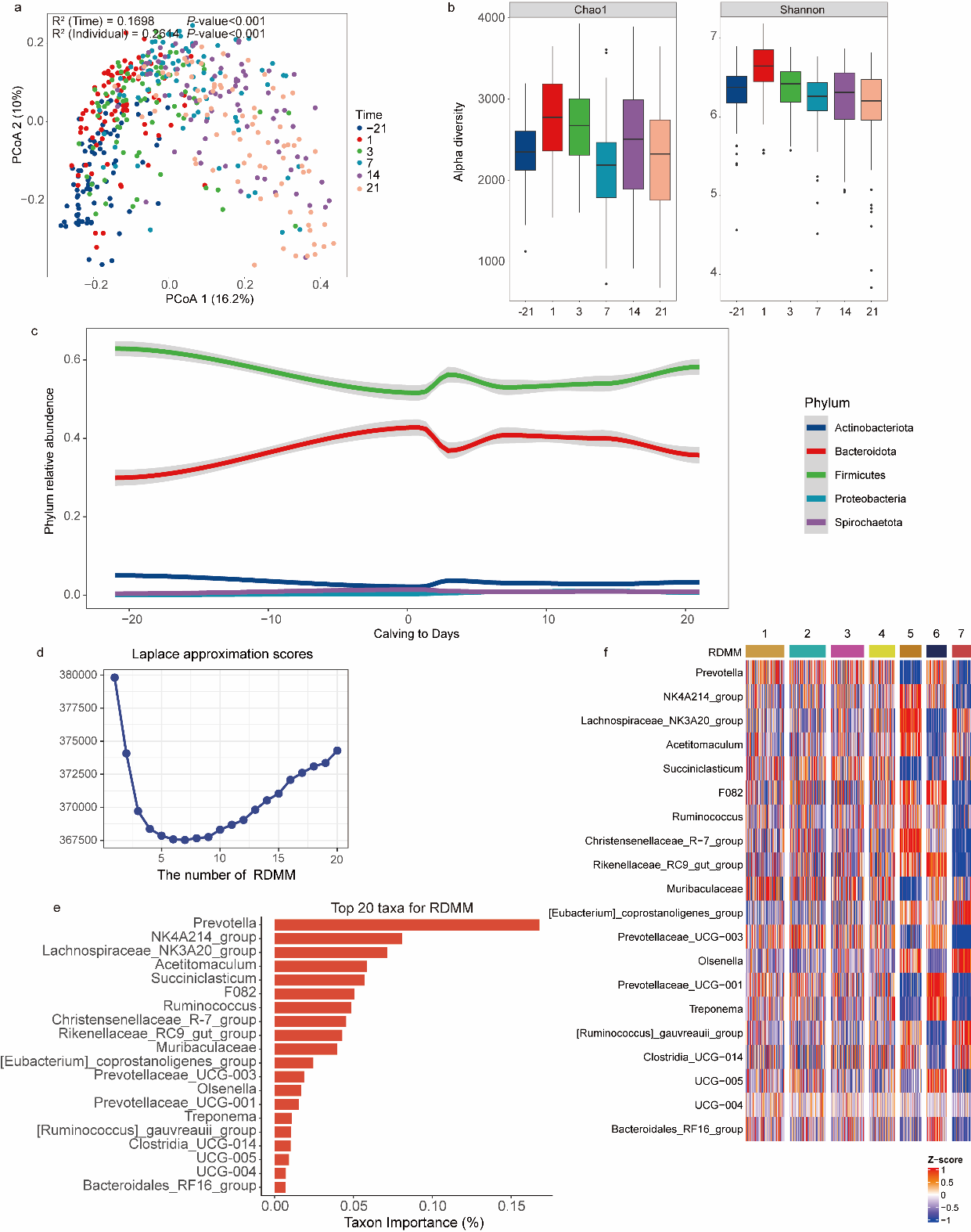


**Fig. S2.** **Dynamics and diversity of fecal microbiota during the periparturient period.** (a) PCoA plot-based Bray–Curtis dissimilarity showing fecal microbiota profiles across different sampling time points. (b) Dynamic changes in the α-diversity of fecal microbiota at different time points during the perinatal period. (c) Plots showing a comparison of the five bacterial phyla with the highest relative abundance in the fecal microbiota of perinatal cows. (d) Laplace approximation scores for fecal DMM. (e) The top 20 taxa that contributed the most to the accuracy of the fecal DMM are shown in the order of importance. (f) Heatmap showing the relative abundance of the top 20 taxa (normalised in all taxa) in each fecal sample.


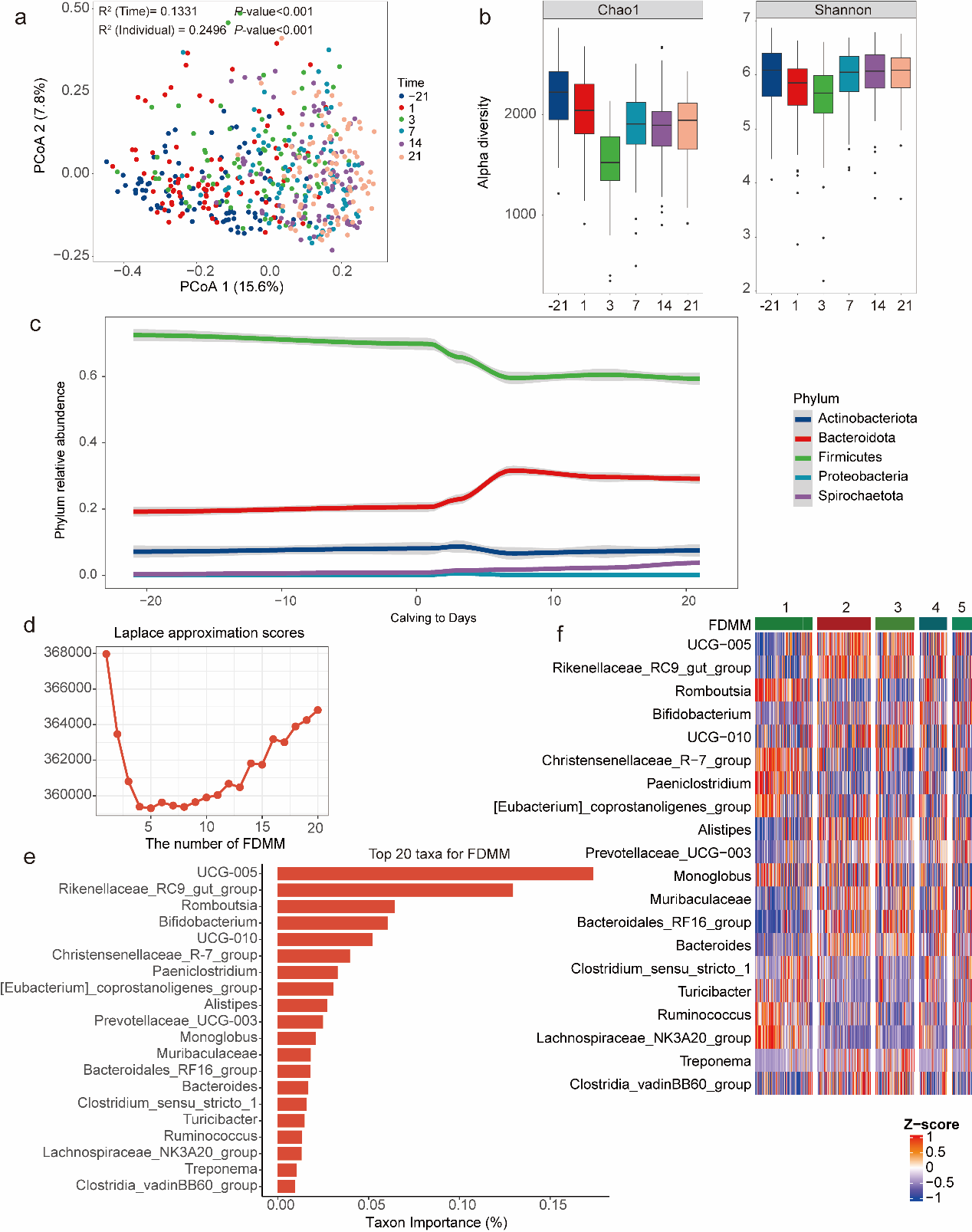


**Fig. S3.** **Cross-validation of ASVs contributing to the random forest model.** (a) Rumen. (b) Feces. ASV, Amplicon sequence variant

**
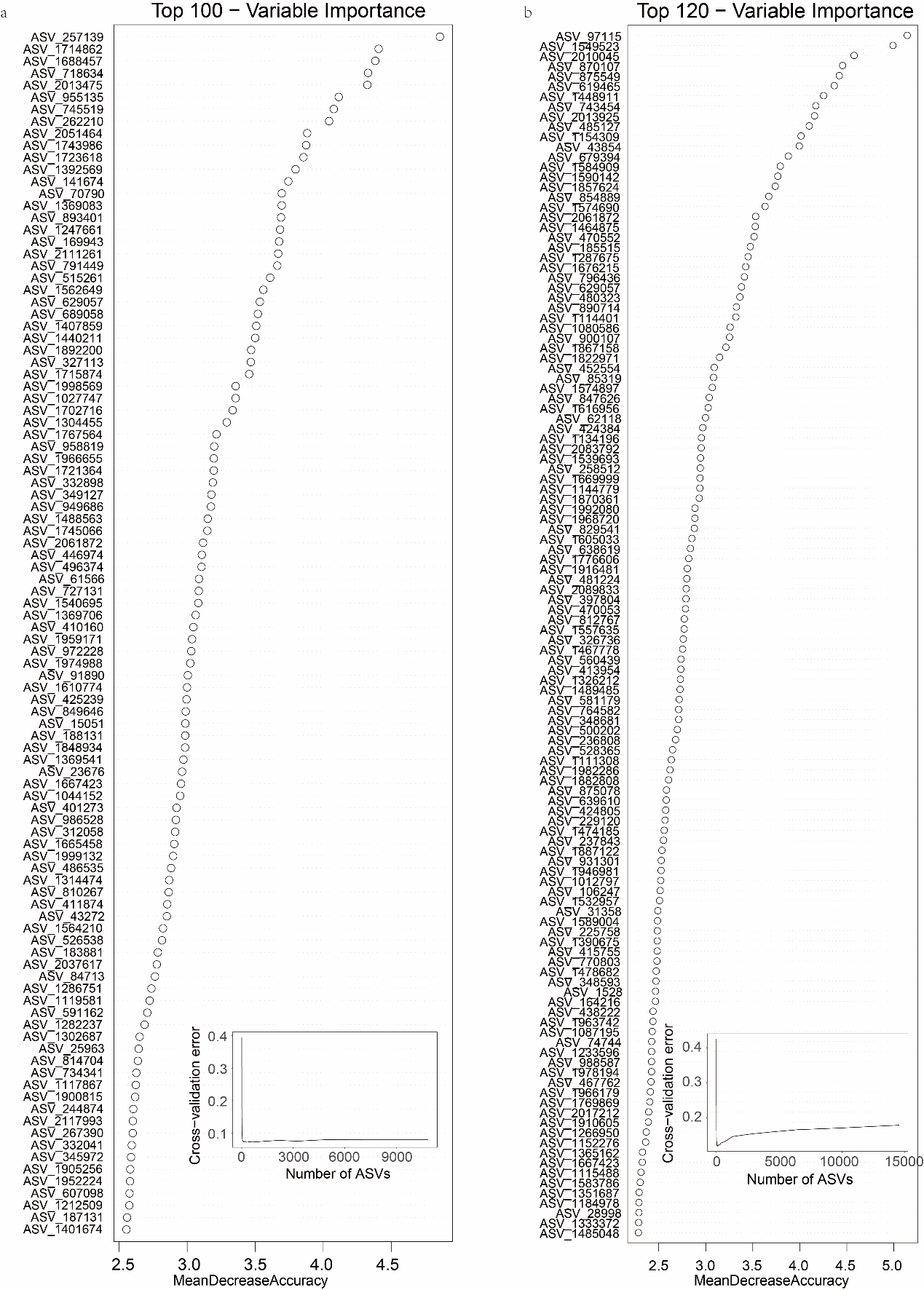
**

**Fig. S4. Relative importance of different ecological processes in the microbiota composition of periparturient dairy cows.** (a) Rumen. (b) Feces. HOS, Homogeneous selection; DL, Dispersal limitation.


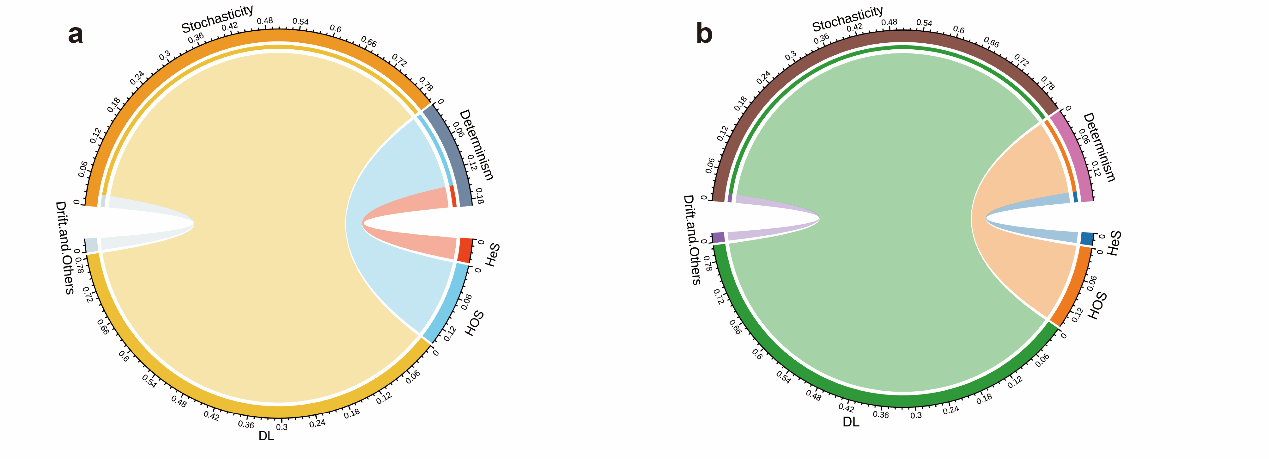

Supplement: giaf042_Supplemental_Files [file giaf042_supplemental_files.zip › Supplementary Figures.docx]
