# Supplementary material for: Deterministic succession patterns in the rumen and fecal microbiome associate with host metabolic shifts in peripartum dairy cattle
Source: Gigascience. 2025 May 19;14:giaf042. doi: 10.1093/gigascience/giaf042 (PMC12087452; doi:10.1093/gigascience/giaf042)
Supplement: giaf042_GIGA-D-24-00404_original_submission [file giaf042_giga-d-24-00404_original_submission.pdf]

GigaScience

Deterministic Succession Patterns of Rumen and Hindgut Microbiome Driving Host Metabolism in Periparturient Ruminants: Insights from a Large-Scale Longitudinal Study of Cows

--Manuscript Draft--

**Manuscript Number:**	GIGA-D-24-00404
**Full Title:**	Deterministic Succession Patterns of Rumen and Hindgut Microbiome Driving Host Metabolism in Periparturient Ruminants: Insights from a Large-Scale Longitudinal Study of Cows
**Article Type:**	Research
**Funding Information:**	National Natural Science Foundation of China (32130100) — Prof.Dr. Shengli Li
**Abstract:**	Background<br><br>Metabolic disorders in periparturient ruminants affect health and productivity, with gut microbiota playing a key role in host metabolism. Therefore, our study aimed to characterize the gut microbiota of periparturient dairy cows to better understand the relationship between metabolic phenotypes and the rumen and hindgut microbiomes during the periparturient period.<br><br>Results<br><br>In a longitudinal study of 91 periparturient cows, we analyzed rumen and hindgut microbiomes via 16S rRNA and metagenomic sequencing across six time points. By employing enterotype classification, ecological modeling, and random forest analysis, we identified distinct deterministic succession patterns in the rumen and hindgut (rumen: rapid transition-transition-stable; hindgut: stable-transition-stable). Key microbes, such as Succiniclasticum and Bifidobacterium, were found to drive microbial succession by balancing stochastic and deterministic processes. Notably, we observed that changes in gut microbiota succession patterns significantly influenced metabolic phenotypes (e.g., serum non-esterified fatty acid, glucose, and insulin level). Causal mediation analysis suggested that specific gut microbes (e.g., Prevotella sp900315525 in the rumen and Alistipes sp015059845 in the hindgut) and metabolic pathways (e.g., glucose-related pathway) were critical factors driving changes in host metabolic phenotypes.<br><br>Conclusions<br><br>Overall, utilizing a large gut microbiome dataset and enterotype- and ecological model-based microbiome analyses, we comprehensively elucidated the succession and assembly of the gut microbiota in periparturient dairy cows. We further confirmed that changes in gut microbiota succession patterns significantly impact the metabolic phenotypes of periparturient dairy cows. These findings provide valuable insights for developing health management strategies for periparturient ruminants.
**Corresponding Author:**	Shengli Li<br>China Agricultural University<br>Beijing, --- Select One --- CHINA
**Corresponding Author Secondary Information:**	
**Corresponding Author's Institution:**	China Agricultural University
**Corresponding Author's Secondary Institution:**	
**First Author:**	Shuo Wang
**First Author Secondary Information:**	

Powered by Editorial Manager® and ProduXion Manager® from Aries Systems Corporation

Order of Authors:	Shuo Wang
	Fanlin Kong
	Dongwen Dai
	Chen Li
	Yangyi Hao
	Erdan Wang
	Zhijun Cao
	Yajing Wang
	Wei Wang
	Shengli Li
Order of Authors Secondary Information:	
Additional Information:	
Question	Response
Are you submitting this manuscript to a special series or article collection?	No
**Experimental design and statistics**<br><br>Full details of the experimental design and statistical methods used should be given in the Methods section, as detailed in our Minimum Standards Reporting Checklist. Information essential to interpreting the data presented should be made available in the figure legends.<br><br>Have you included all the information requested in your manuscript?	Yes
**Resources**<br><br>A description of all resources used, including antibodies, cell lines, animals and software tools, with enough information to allow them to be uniquely identified, should be included in the Methods section. Authors are strongly encouraged to cite Research Resource Identifiers (RRIDs) for antibodies, model organisms and tools, where possible.<br><br>Have you included the information requested as detailed in our Minimum Standards Reporting Checklist?	Yes

Powered by Editorial Manager® and ProduXion Manager® from Aries Systems Corporation

**Availability of data and materials**<br><br>All datasets and code on which the conclusions of the paper rely must be either included in your submission or deposited in publicly available repositories (where available and ethically appropriate), referencing such data using a unique identifier in the references and in the "Availability of Data and Materials" section of your manuscript.<br><br>Have you have met the above requirement as detailed in our Minimum Standards Reporting Checklist?	Yes

Powered by Editorial Manager® and ProduXion Manager® from Aries Systems Corporation

Click here to access/download;Manuscript;Manuscript.docx

Deterministic Succession Patterns of Rumen and Hindgut Microbiome Driving Host Metabolism in Periparturient Ruminants: Insights from a Large-Scale Longitudinal Study of Cows

Shuo Wang†, Fanlin Kong†, Dongwen Dai, Chen Li, Yangyi Hao, Erdan Wang, Zhijun Cao, Yajing Wang, Wei Wang*, and Shengli Li*

State Key Laboratory of Animal Nutrition and Feeding, Department of Animal Nutrition and Feed Science, College of Animal Science and Technology, China Agricultural University, Beijing 100193, China

***Correspondence:**

Shengli Li: lishengli@cau.edu.cn; Tel and fax: +86-010-62731254;

Wei Wang: wei.wang@cau.edu.cn; Tel and fax: +86-010-62733789

†These authors contributed equally: Shuo Wang and Fanlin Kong

Abstract

Background: Metabolic disorders in periparturient ruminants affect health and productivity, with gut microbiota playing a key role in host metabolism. Therefore, our study aimed to characterize the gut microbiota of periparturient dairy cows to better understand the relationship between metabolic phenotypes and the rumen and hindgut microbiomes during the periparturient period.

Results: In a longitudinal study of 91 periparturient cows, we analyzed rumen and hindgut microbiomes via 16S rRNA and metagenomic sequencing across six time points. By employing enterotype classification, ecological modeling, and random forest analysis, we identified distinct deterministic succession patterns in the rumen and hindgut (rumen: rapid transition-transition-stable; hindgut: stable-transition-stable). Key microbes, such as *Succiniclasticum* and *Bifidobacterium*, were found to drive microbial succession by balancing stochastic and deterministic processes. Notably, we observed that changes in gut microbiota succession patterns significantly influenced metabolic phenotypes (e.g., serum non-esterified fatty acid, glucose, and insulin level). Causal mediation analysis suggested that specific gut microbes (e.g., *Prevotella sp900315525* in the rumen and *Alistipes sp015059845* in the hindgut) and metabolic pathways (e.g., glucose-related pathway) were critical factors driving changes in host metabolic phenotypes.

Conclusions: Overall, utilizing a large gut microbiome dataset and enterotype- and ecological model-based microbiome analyses, we comprehensively elucidated the succession and assembly of the gut microbiota in periparturient dairy cows. We further confirmed that changes in gut microbiota succession patterns significantly impact the metabolic phenotypes of periparturient dairy cows. These findings provide valuable insights for developing health management strategies for periparturient ruminants.

Keywords: microbiome, dynamics, longitudinal study, cow, metabolic phenotypes, periparturient period

Data Description

We conducted a 42-day dynamic follow-up study on 91 healthy periparturient dairy cows, tracking changes in the rumen microbiome (476 16S rRNA sequencing samples and 30 metagenomic sequencing samples), hindgut microbiome (506 16S rRNA sequencing samples and 30 metagenomic sequencing samples), and metabolic phenotypes (505 samples). We established relationships between changes in the gut microbiome and metabolic phenotypes. The metagenomic and 16S rRNA sequencing data used in this study have been archived in the NCBI database under accession numbers PRJNA1161368 and PRJNA1126601, respectively.

Introduction

Ruminants play a crucial role in global food supply and sustainable agriculture [1]. The periparturient period (three weeks before and after calving) is one of the most vulnerable times in a ruminant's life. During this phase, due to calving, dietary changes, and onset of lactation, ruminants undergo substantial physiological and metabolic adjustments [2-4]. Approximately 30–50% of dairy cows experience postpartum metabolic diseases, including ketosis, hypocalcemia, and retained placenta [4, 5]. These conditions affect the health and productivity of ruminants and lead to substantial economic losses [6]. Ruminants have evolved a unique rumen structure that enables multiple host-microbiome interactions: host-rumen microbiome and host-hindgut microbiome interactions. The rumen microbiome is essential for cellulose breakdown, short-chain fatty acid production, nitrogen cycling, and vitamin synthesis [7]. The hindgut microbiome contributes to energy and nutrient absorption and modulates the host immune system [8]. Additionally, extensive research in adult dairy cows showed correlations between the rumen and hindgut microbiomes with milk quality and feed efficiency [9, 10], further highlighting their critical role in ruminant production and health.

In recent years, succession and assembly of the gut microbiome has received increased attention. Microbial succession refers to changes in gut microbiome composition over time, while microbiome assembly involves the formation and development of specific microbial communities within the gut [11, 12]. This process includes colonization of various microbial species and their interactions with each other and the host environment [12]. Understanding microbial succession and assembly is crucial for

comprehending how microbial communities are established stably and functionally. This helps understand how these microbial communities impact health. Teddy and Xiao *et al.* identified that the infant gut microbiome can be roughly divided into three succession stages: development, transition, and stable stages [13, 14]. Additionally, they used neutral ecological models to elucidate transformation patterns and driving forces of the infant gut microbiome [14]. Fu *et al.* found that the *Aspergillus* enterotype in pregnant women remains stable over time and is associated with host health [15]. In animals, studies on chicks [16], piglets [17], lambs [18], and calves [19, 20] have highlighted how gut microbiome assembly affects the growth and development of young animals. These studies used large-scale longitudinal methods to explore the succession and assembly patterns of the gut microbiome and their dynamic interactions with the host, providing targeted evidence for promoting healthy development and pregnancy via gut microbiome regulation.

Recently, some studies have attempted to reveal the succession patterns of the rumen and hindgut microbiome in periparturient ruminants (primarily focusing on cows) [21-25]. However, due to limitations in sample size and temporal resolution, different studies have found varying patterns of change in the gut microbiome of dairy cows. Zhu *et al.* observed a decrease in the richness of rumen microbiota from the prenatal to the postnatal stages [22]. In contrast, Bach *et al.* found an increase in the richness of rumen microbiota [23]. Moreover, although Zhu *et al.* noted that prepartum fecal microbiota exhibits higher diversity, primarily composed of Firmicutes and Bacteroidetes [24], Luo *et al.* reported no marked differences in diversity before and after calving, with slight differences in phylum composition [25]. Although these studies indicate varying results, they consistently suggest the potential for remodeling the gut microbiome in periparturient cows. Considering the substantial metabolic changes during the periparturient period, there remains a gap in our understanding of the succession patterns of rumen and hindgut microbiomes and their impact on host metabolism. Additionally, exploring key factors driving these microbiome dynamics is crucial. Understanding these factors could potentially allow us to predict and determine when and how to intervene in the microbiome assembly process to modulate its structure and function. Besides normal dynamic changes, individual factors (e.g., parity and body condition) have previously been reported to correlate with ruminant gut microbiomes [26, 27]. A comprehensive analysis of these factors will aid in better understanding the

dynamic changes of the microbiome in periparturient ruminants.

In this study, we used dairy cows with highly controlled feeding systems, diets, and housing as our subjects. We selected periparturient cows with normal health through longitudinal observations of the entire cohort. The strict control of normal cows allowed us to minimize interference from other macro factors, providing a clearer view of the natural dynamic changes in the gut microbiome and key factors driving these changes. Over a 42-day periparturient period, we collected rumen, fecal, and blood samples from 91 healthy cows at six time points. By analyzing the periparturient dairy cow gut microbiome profiles (using the Dirichlet multinomial mixtures method) and applying ICAMP (a phylogenetic bin-based null model) and Markov chain methods, our study offers comprehensive insights into dynamic changes and transitions of the gut microbiome. It also enhances our detailed understanding of the gut microbiome and host metabolic characteristics in periparturient ruminants, potentially facilitating the development of new strategies to improve postpartum health in ruminants.

Results

Dynamic changes in gut microbial composition in periparturient dairy cows

Through a rigorous prospective cohort study design (Fig. 1), we observed a clear separation of gut microbiota in periparturient dairy cows among the sampling time points, underscoring the remodeling of the cow gut microbiota during periparturient periods (Figs. S1a and S2a). The α-diversity (Chao1 and Shannon indexes) of ruminal microbiota initially exhibited an increasing trend from prepartum to postpartum, subsequently decreasing and finally stabilizing (Fig. S1b). Conversely, the α-diversity of fecal microbiota showed an initially decreasing trend, followed by an increase and ultimately stabilizing (Fig. S2b). We also observed drastic changes in the α-diversity of rumen microbiota at just 1 day postpartum. Conversely, fecal microbiota showed a similar response but at 3 days postpartum. Additionally, in both rumen and fecal samples, the dominant microbial phyla were Firmicutes, Bacteroidetes, Actinobacteria, Spirochaetes, and Proteobacteria (Figs. S1c and S2c).

Importantly, we observed significantly greater inter-individual than intra-individual variability across both rumen and fecal microbiota (Figs. 2a and f), and the inter-individual variation in the rumen and fecal microbiota constituted 26.14% and 24.96% of the total compositional variation, respectively

(PERMANOVA, $p<0.01$). These results highlight their individualized states in periparturient dairy cows. To more accurately demonstrate microbial succession in the rumen fluid and feces of periparturient cows, we applied the DMM method. At the genus level, based on the lowest Laplace approximation scores (Figs. S1d and S2d), the analysis yielded seven and five DMM clusters for rumen and fecal samples, respectively (Figs. 2b and g). Regarding ruminal microbiota, *Prevotella*, *NK4A214_group*, *Lachnospiraceae_NK3A21_group*, *Acetitomaculum*, and *Succiniclasticum* were the top five genera defining RDMMs (DMM clusters for rumen microbiota; Fig. S1e). The heatmap in Figure S1f displays the distribution of these genera across different RDMMs. Each RDMM exhibited a unique timing of appearance and dominant genera (Fig. 2c and d). RDMM1 was dominated by *Succiniclasticum*, prominently appearing until 7 d postpartum; RDMM2, featuring *Muribaculum* and *Prevotella*, appeared from 1 d postpartum; RDMM3, dominated by *Prevotellaceae_UCG_003*, was prominent on days 3 and 7 postpartum; on the other hand, RDMM4 and 5, which were active primarily on 21 d prepartum, gradually diminished postpartum. In particular, *FO82* was identified for RDMM4, while *Lachnospiraceae_NK3A21_group*, *Christensenellaceae_R_7_group*, *Acetitomaculum*, *NK4A214_group*, and *Ruminococcus* were the dominant genera for RDMM5; finally, RDMM6 emerged on the 1 d postpartum with dominant genera including *Rikenellaceae_RC9_gut_group*, *Prevotella_UCG-001*, and *Treponema*, gradually diminishing over time; By the 3 d postpartum, RDMM7, characterized by the *[Eubacterium]_coprostanoligenes_group*, *Olsenella*, and *[Ruminococcus]_gauvreauii_group*, started to appear. In addition to the occurrence windows, we noted the changing community characteristics of the rumen clusters over time. The timeline of dominance shifted from RDMM4 and RDMM5 prepartum to RDMM6 on 1 d postpartum, then to RDMM3 by 3 d, RDMM2 by 7 d, and finally to RDMM1 by 14 and 21 d (Fig. 2c). Further analysis indicated that except for the notable instability of RDMM3, the Shannon diversity of the other RDMM clusters remained relatively stable across different periparturient age points (Fig. 2e).

In the fecal microbiota, the top five genera defining FDMM clusters were *UCG-005*, *Rikenellaceae_RC9_gut_group*, *Romboutsia*, *Bifidobacterium*, and *UCG-010* (Fig. S2e). The heatmap in Figure S2f illustrates the distribution of these genera across different FDMMs. Similarly, FDMMs exhibited distinct occurrence windows and dominant genera (Figs. 2h and i). FDMM1, characterized by

dominant genera, including *Romboutsia*, *Paeniclostridium*, *Lachnospiraceae_NK3A20_group*, and *Christensenellaceae R-7 group*, was primarily observed at 21 d prepartum; the abundance of FDMM1 gradually decreased, and finally, it disappeared postpartum. FDMM2 and FDMM3 appeared prepartum, and their abundance increased postpartum, with FDMM2 being characterized by *Rikenellaceae_RC9_gut_group*, *Muribaculaceae*, and *Alistipes*, and FDMM3 by *Bacteroidales_RF16_group* and *Prevotellaceae_UCG_003*. FDMM4 did not exhibit any dominant genera, indicating its instability, whereas FDMM5 was dominated by Bacteroides. The two clusters were present throughout the periparturient period, peaking at 3 d postpartum. Importantly, the community characteristics of fecal clusters varied over time (Fig. 2h). FDMM1 was dominant on 21 d prepartum and 1 d postpartum, experiencing a shift on 3 d postpartum with no dominant cluster, and stabilized by 7–21 d postpartum, with FDMM2 and 3 becoming dominant. Unlike in the rumen, the Shannon diversity of FDMM1, 2, and 3 initially decreased and then stabilized, whereas that of FDMM4 continued to decrease, and that of FDMM5 fluctuated (Fig. 2j).

Dynamics of the individual gut microbiota in periparturient cows

To delve deeper into the transformation process of microbial community clusters in the rumen and feces in periparturient cows, we analyzed their transitions across different sampling days at the individual level. We observed a distinct trend of transitions between the community clusters throughout the study period (Figs. 3a and 4a; Tables S1 and S2). Specifically, from 21 d prepartum to 1 d postpartum, the RDMM4 to 6 and RDMM5 to 6 transitions represented 24.1% and 16.5% of the total transitions in the rumen, respectively, highlighting them as the dominant transformations (Table S1). Similarly, the RDMM6 to 3 transition was the dominant transformation from 1 d to 3 d postpartum; RDMM3 to 2 and RDMM3 to 3 were the dominant transformations from 3 d to 7 d postpartum; RDMM2 to 1 and RDMM2 to 2 were the dominant transformations from 7 d to 14 d postpartum; and RDMM1 to 1 was the dominant transformation from 14 d to 21 d postpartum (Table S1). Regarding fecal microbiota, the FDMM1 to 1 transition was the dominant transformation from 21 d prepartum to 3 d postpartum. Interestingly, we did not observe any dominant transformations from 3 d to 7 d postpartum, highlighting the instability of the fecal microbial community structure during this period; however, the FDMM2 to 2

and FDMM3 to 3 transitions dominated from 7 d to 21 d postpartum, suggesting a return to stability (Table S2).

To quantitatively integrate the transitions of microbial community types in the rumen and feces during the peripartum period, we developed a Markov chain model (Figs. 3b and 4b). For rumen, we found that RDMM2, 3, 4, 5, and 6 had high frequencies of transitioning to other clusters, with RDMM4, 5, and 6 exhibiting lower self-transition rates (below 20%), likely contributing to their rapid disappearance during the succession process. Conversely, RDMM2 and 3 exhibited self-transition rates of 45% and 35%, respectively. These two RDMMs were more stable and may thus play a role in bridging microbial succession. In addition, RDMM1 and 7, with self-transition rates of 58% and 64%, respectively, and mutual transition rates above 30%, exhibited stability and maturity during the later stages of the peripartum period. Thus, peripartum microbial succession could be divided into three phases: rapid transition (RDMM4, 5, and 6), transition (RDMM3 and 2), and stabilization (RDMM1 and 7). Based on the above microbial succession patterns, we used a random forest algorithm to construct a classification model. Following five rounds of ten-fold cross-validation, we determined that the model constructed using the top 100 ASVs with the highest accuracy exhibited the highest prediction rate (AUC>0.95) (Figs. S3a and Fig. 3c). In addition, we found that some of the previously mentioned genera, including *Rikenellaceae_RC9_gut_group*, *Prevotella*, *Acetitomaculum*, and *F082*, played key roles in constructing the classification model (Fig. S3a).

In the study of fecal microbiota during the peripartum period, we also observed distinct self-transfer rates among FDMMs. Specifically, FDMM 4 and 5 exhibited lower self-transfer rates of 26% and 23%, respectively. In contrast, FDMM 1, 3, and 2 displayed higher rates of 41%, 46%, and 66%, respectively (Fig. 4b). The conversion rate of FDMM 4 and 5 to FDMM 1 reached 16%, suggesting that during the stage of a rapid transition in the rumen microbiota structure, the fecal microbiota composition remained relatively stable, potentially due to a delayed response to calving stress. The mutual transfer rates between FDMM 2 and 3 exceeded 20%, reflecting their relative stability and maturity during the microbial succession process. Compared with rumen microbiota, fecal microbiota transitioned into a stable phase within 7 d postpartum, illustrating a shorter transition period. Based on these findings, we categorized fecal microbiota succession into three distinct stages: stabilization (FDMM1), transition

(FDMM4 and 5), and stabilization (FDMM2 and 3). Using the top 120 ASVs with a random forest algorithm, we developed a classification model, which after rigorous cross-validation showed high predictive accuracy (AUC > 0.95) (Fig. S3b and Fig. 4c). We observed that key genera, including *Romboutsia*, *UCG-005*, *Paeniclostridium*, and *Bifidobacterium* played pivotal roles in this model, further underscoring their importance in predicting periparturient fecal microbiota succession patterns (Fig. S3b).

Assembly mechanism of rumen and fecal microbes in periparturient dairy cows

We demonstrated that microbial succession in the rumen and feces of periparturient dairy cows progresses through three stages. Understanding the underlying reasons for these transformations is of great interest. Therefore, we explored the internal driving forces of rumen and fecal microbiota using ICAMP of ecological models to elucidate potential factors influencing microbial community dynamics. We found that the microbial structure in both the rumen and feces of periparturient dairy cows was primarily dominant by stochastic processes, accounting for 81% and 82%, respectively, with dispersal limitation (DL) contributing the most to stochastic processes, whereas homogeneous selection (HOS) appeared to play the largest role in deterministic processes (Fig. S4a and b). We further compared the ecological processes of different microbial clusters, focusing primarily on HOS and DL because of the minor relative contributions of other processes (heterogeneous selection, homogenizing dispersal, and drift). We found significant differences in the HOS and DL processes among the different ruminal and fecal succession patterns (Fig. 5a and Fig. 6a), indicating that ecological processes drive the succession of microbial communities in the rumen and feces of periparturient cows. In the rumen, we observed a significant decline in the proportion of DL processes during the succession period, whereas the proportion of HOS processes increased. Conversely, the fecal microbiota exhibited higher DL and lower HOS during succession, highlighting the key role of deterministic and stochastic processes in the succession of ruminal and fecal microbiota during the periparturient period, respectively.
Furthermore, we divided ruminal and fecal ASVs into 144 and 137 Bins, respectively (Tables S3 and S4). In the rumen, deterministic HOS dominated three of the top 20 relative abundances of RBins (Bins for rumen microbiota), whereas DL dominated the remaining 17 RBins (Fig. 5b). Conversely, in feces,

HOS dominated two of the top 20 relative abundances of FBins (Bins for fecal microbiota), whereas DL dominated the remaining 18 FBins (Fig. 6b). We also presented the abundance and ecological process contributions of the top 20 Bins in the rumen and feces at different successional stages (Figs. 5c and Fig. 6c). In the rumen, *Succiniclasticum*, *Prevotellaceae_UCG-001*, *Prevotella*, *[Eubacterium]_coprostanoligenes_group*, and *Olsenella* were identified as key drivers of microbial succession, with their changes in relative abundance across different stages aligning with their contributions to the HOS process (Figs. 5d and e). Similarly, in feces, *Bifidobacterium*, *Treponema*, *UCG-005*, *Lachnospiraceae_AC2044_group*, *Rikenellaceae_RC9_gut_group*, and *Monoglobus* were identified as key drivers of microbial succession, with their relative abundance changes across stages consistent with their contributions to the DL process (Figs. 6d and e).

Contribution of multiple individual factors to gut microbial succession

To assess the influence of individual factors on gut microbial succession, the samples were stratified into all and three successional stages for covariate analysis (Fig. 7). We found that factors such as diet, parity, age, calving to days (CD), and pH were associated with rumen microbial succession. Specifically, CD and dietary nutrient levels explained most of the variance in rumen stage 1(RS1), CD and birth weight explained most of the variance in rumen stage 3 (RS3), while rumen stage 2 (RS2) was most strongly related to sire. For fecal microbiota, CD, diet, and age were significant factors associated with microbial succession, with CD and diet explaining most of the variance in fecal stage 1 (FS1). Fecal stage 2 (FS2) was associated with factors such as sire, pH, and predelivery – actual data, and sire also explained the largest variance in fecal stage 3 (FS3).

Furthermore, we further examined the effects of individual factors on key taxa in community assembly using the Massline2 method (Table S5). In the rumen, the relative abundance of *Succiniclasticum*, *Prevotella*, *Prevotellaceae_UCG-001, Olsenella*, and *[Eubacterium]_coprostanoligenes_group* was most strongly associated with CD, diet, and pH. In the hindgut, the results show that the relative abundance of *Lachnospiraceae_AC2044_group* was associated with sire and milk yield in last parity, and the relative abundance of *Monoglobus* was related to diet. However, other key taxa were not associated with these individual factors.

Gut microbial succession types influence host metabolic phenotypes in periparturient dairy cows

First, we assessed the dissimilarity at the ASV level between the rumen and fecal microbiota, yielding an M^2 value of 0.79 (Fig. 8a). Further source tracking analysis showed that approximately 80% of the fecal microbiota originated from fecal microbiota at last time point, whereas only 4% could be traced back to rumen microbiota at the same time point (Fig. 8b). These results suggest a weak link between the rumen and fecal microbiomes, supporting their consideration as distinct units when studying their effects on the metabolism of peripartum dairy cows. Additionally, the rumen and fecal microbiomes contributed to changes in blood metabolic indicators by 20.64% and 19%, respectively (Table S6). Concomitantly, we did not observe any significant difference in the residuals between these microbiome and blood indicators, further supporting their similar contributions to the host metabolism (Fig. 8c). Based on these findings, we separately counted the transformation types of gut microbiota and analyzed effects of the different types on host metabolic phenotypes at the individual level (Figs. 8d and f). Significant differences in blood metabolic indicators were observed across different transformation types (Figs. 8e and g). Transformation types in the rumen microbiota significantly influenced host change levels of serum NEFA, IGF-1, RBHB, and GLU ($p < 0.05$), while transformation types in the fecal microbiota notably affected serum INS, TG, NEFA, BHBA, and IGF-1 change levels ($p < 0.05$).

Transition in the metabolic capacity of the gut microbiome and microbe–host interactions in periparturient dairy cows

To validate our bacterial findings and evaluate functional succession transitions in rumen and hindgut microbiome, we analyzed microbial and functional changes across succession stages using metagenomic data. Rumen and hindgut microbiome clustered distinctly at the species level (Fig. 9a), and network analysis also revealed significant stage-specific differences in microbial interactions (Figs. 9b and c). For example, in stable stages of hindgut (FS1 and FS3), species showed simpler interactions and lower centrality (Fig. 9c). Meanwhile, the node analysis results also revealed that key taxa driving microbial succession, such as ruminal *Prevotella* and *Succiniclasticum*, as well as fecal *Bifidobacterium* and *Treponema*, played pivotal roles in the network composition (Tables S7-12).

Similarly, rumen and hindgut microbiota clustered separately at the pathway level (Fig. 9d). We found that the RS2 and RS3 stages were more similar, while FS2 and FS1 stages were closer. Despite having different dominant species at various successional stages, these results further suggest that the successional patterns of rumen and hindgut microbiota during the periparturient period are fundamentally different. In addition, metabolic pathways also displayed stage-specific patterns (Figs.9e and f). In the rumen, the RS1 stage was characterized by upregulated pathways involved in pyruvate fermentation, glycolysis, nucleotide biosynthesis, and amino acid metabolism. In the RS3 stage, there was an upregulation of pathways related to the Bifidobacterium shunt, glycogen degradation, and aromatic amino acid biosynthesis. Notably, polyamine biosynthesis, gluconeogenesis, and coenzyme A synthesis were consistently upregulated in both RS2 and RS3 stages. In the hindgut, metabolic pathways such as sulfate reduction and sulfur metabolism, methanogenesis V, and the incomplete reductive TCA cycle were upregulated in the FS1 stage. The FS3 stage exhibited upregulation of pathways related to D-galacturonate degradation I, D-fructuronate degradation, glycolysis IV, L-lysine biosynthesis, and UMP biosynthesis. Common pathways such as inosine-5'-phosphate biosynthesis, glycolysis I and II, mixed acid fermentation, and thiamine metabolism were upregulated in both FS1 and FS2 stages.

Further, we used mediation models to explore the relationships between significantly different species, metabolic pathways, and metabolic phenotypes. In the rumen, we identified 280 relationships with mediating effects, 19 of which showed mediation, direct, and total effects (Table S13). For example, rumen *Prevotella sp900315525* and Pyrimidine deoxyribonucleotides de novo biosynthesis II promotes the elevation of serum NEFA levels. In contrast, the elevated abundance of *UBA3839 sp900314125* and glycolysis I as well as *UBA2813 sp902801985* and L-lysine biosynthesis I reduce serum NEFA levels (Fig. 9g). In the hindgut, we identified 1,360 relationships with mediating effects, 41 of which showed mediation, direct, and total effects (Table S14). For example, the abundance of *CAG-791 sp902780385* and starch degradation III was associated with increased serum INS levels. Conversely, the increased abundance of *Treponema-D sp017381365* and glycolysis III as well as *Alistipes sp015059845* and gluconeogenesis I reduce serum TG and INS levels (Fig. 9h).

Discussion

To our knowledge, this study is the first to systematically investigate the gut microbiomes of periparturient dairy cows using a large-scale, high-frequency sampling approach. We also uniquely employed a combination of microbial clustering, ecological model, and random forest analysis to reveal that the rumen and hindgut microbiome succession in periparturient dairy cows can be divided into three distinct stages. The results showed significant differences in the composition and function of rumen and hindgut microbiota across different successional stages. Moreover, the transitions between these stages were driven not only by different taxa but also significantly influenced metabolic phenotype changes in periparturient dairy cows.

As expected, we observed significant changes in the microbial structure of the rumen and hindgut in periparturient dairy cows. However, compared to previous studies, the trends in Chao1 and Shannon indices of the rumen and hindgut microbiomes in our study showed unique characteristics [22-25]. Notably, these previous studies were conducted on the same breed of cows with similar periparturient diets—high-fiber diets before calving and high-starch diets postpartum. This underscores the importance of large sample sizes and high temporal resolution in revealing changes in gut microbiome. Furthermore, we found that individual differences had an even greater impact on gut microbiome structure throughout the periparturient period than changes observed within individuals over time. To minimize individual variability and enhance the interpretability of microbial structure, we applied microbial clustering, Markov chains, and random forest methods to analyze the dynamic changes in rumen and hindgut microbiota during the periparturient period. We identified three deterministic successional stages in both the rumen (rapid transition, transition, stabilization) and feces (stabilization, transition, stabilization), reflecting distinct adaptive strategies of gut microbiota under periparturient stress. Rumen microbiota were more sensitive to stress, while hindgut microbiota exhibited a delayed response, a characteristic also observed in the study by Bach *et al.* [23]. Thus, our research provides new insights into the transitional dynamics of gut microbiota in dairy cows during the periparturient period.

Additionally, we innovatively used ecological models to estimate the assembly processes of rumen and hindgut microbiota in dairy cows. As succession progressed, we found that the HOS (Homogeneous Selection) process significantly increased in the rumen microbiota, whereas the DL (Dispersal

Limitation) process increased notably in the hindgut microbiota. This indicates that the microbial succession of rumen during the periparturient period is driven by deterministic processes, while the microbial succession of hindgut is primarily driven by stochastic processes. HOS suggests that under environmental pressure, microbial community compositions tend to become homogeneous, emphasizing the dominant role of environmental factors in shaping microbial community structures [28, 29]. In contrast, DL refers to changes in the relative abundance of species in the microbial community due to random events (including random deaths or births), implying that ecological niches in the community are not completely occupied or utilized [30, 31]. Our findings attribute the HOS process in rumen microbiota primarily to the active response of taxa such as *Succiniclasticum*, which are involved in fiber degradation, volatile fatty acid production, and the synthesis of succinate and glucogenic amino acids [32-35]. Meanwhile, the DL process in hindgut microbiota was mainly due to the active response of taxa such as *Bifidobacterium*, which play critical roles not only in fiber decomposition and volatile fatty acid production but also in maintaining gut homeostasis, preventing overgrowth of harmful bacteria, and reducing the risk of intestinal inflammation and related diseases [36-39]. These findings further support the results of Shen *et al*. [40], which suggested that the hindgut microbiome in ruminants more accurately reflects the host health status compared to rumen microbiome. Interestingly, our study revealed that individual characteristics, including sire, parity, calving to days, diet, and pH, significantly influenced the succession and assembly of both rumen and hindgut microbiota. Previous studies have already confirmed the impact of diet on community succession in dairy cows [41]. Combined with the increase in days after calving, this indirectly suggests that feed intake significantly affects microbial assembly, as postpartum cows exhibit a linear increase in feed intake [42]. The finding related to the sire factor is particularly intriguing; previous research on humans, pigs, sheep, and beef cattle has demonstrated significant correlations between host SNPs and their gut microbiome [43-46], underscoring the potential of genetic breeding for altering the gut microbiota composition of periparturient dairy cows to reduce the incidence of metabolic diseases.

Importantly, this study observed a low similarity between rumen and hindgut microbiome, yet their substantial contributions to the metabolic regulation of dairy cow hosts were evident. Therefore, future research should consider rumen and hindgut microbiomes as two separate systems for studying their

interactions with the host. We also found that the types of microbial transition significantly impacted blood indicators associated with metabolic disorders in cows. Specifically, the rumen microbiome primarily influenced glucose metabolism, as reflected by serum NEFA and GLU levels, whereas the hindgut microbiome mainly affected lipid metabolism, including serum INS and TG levels. These results suggest that metabolic disorders in postpartum cows may stem from improper microbial succession patterns. For instance, a direct transition from stage 1 to 3 in the rumen microbiome significantly decreased serum NEFA levels, whereas stage 1 to 3 in the in the hindgut microbiome notably increased serum INS levels. It is reported that serum elevated NEFA and decreased INS levels are hallmark phenotypes of postpartum metabolic disorders in dairy cows [4, 47]. Thus, we propose that promoting rapid transitions in gut microbiome could be an effective strategy to prevent metabolic disorders in postpartum cows. Furthermore, we observed that the hindgut microbiome underwent a shorter transitional phase than the rumen microbiome, stabilizing by 7 d postpartum (the rumen microbiome stabilized at 14 d postpartum). Numerous studies have documented that postpartum cows are particularly susceptible to metabolic and inflammatory diseases within the first 10 days after calving [4, 48]. Research in model animals also indicates that gut dysbiosis can lead to disruptions in insulin secretion and lipid metabolism [49]. Therefore, we suggest that regulating the hindgut microbiota could be more beneficial in preventing metabolic diseases, given its transition period precedes the typical onset of such conditions. Taken together, we believe that the rumen microbiome primarily functions to supply substantial energy to dairy cows, while the hindgut microbiome focuses on maintaining homeostasis to ensure the health of periparturient cows. Notably, key microbes can influence these metabolic phenotypes through pathways involving glucose metabolism, amino acid synthesis, and starch degradation. The glucose-related pathway in both the rumen and feces mediates the impact of key microbes on host metabolism, highlighting the crucial role of glucose supplementation in postpartum diets for maintaining dairy cow health. Previous studies have linked *Alistipes* to short-chain fatty acid (SCFA) production, including acetate and propionate, and its reduced abundance is associated with disease progression, including non-alcoholic fatty liver disease and non-alcoholic steatohepatitis, owing to decreased SCFA levels [50, 51]. Moreover, *Alistipes* may inhibit tumor growth and recruitment of proinflammatory cells to the liver by enhancing the functions of anti-inflammatory cell subsets and

SCFA levels [52, 53]. Postpartum dairy cows are susceptible to fatty liver. Thus, *Alistipes sp015059845* in the hindgut may play a vital role in maintaining liver and intestinal health in postpartum dairy cows. This study is the first to comprehensively provide a dynamic perspective on the gut microbiome of periparturient dairy cows, enhancing our understanding of the dynamic interactions between the gut microbiome and the host during the periparturient period. Since this study only examined healthy cows, our next research objective is to compare the microbiomes of healthy and diseased cows to explore mechanisms related to postpartum metabolic disorders. Additionally, this study lacks information on feed intake, which is a critical factor influencing gut microbiome composition. In future research, we plan to investigate the impact of feed intake on the dynamic changes in the gut microbiome of periparturient dairy cows. Moreover, based on the patterns observed in this study, further research is needed to verify whether modifying the succession process of the gut microbiome by targeting the identified key microbes and pathways can effectively regulate host metabolism in periparturient cows, thereby preventing postpartum metabolic disorders.

Materials and Methods

Animals, experimental design, and sample collection

This study was conducted at a Commercial Dairy Farm in Shanxi Province, China. The dairy cows involved in the experiment were managed using a traditional feeding model. Two months before the expected calving date, the cows were moved to a dry cow barn and started on a high-fiber total mixed ration (TMR) (dry period) diet. After calving, the cows were immediately separated from their calves to prevent further exposure of the dams to microbes from the calves. The cows were then moved to a transition barn where professional technicians immediately milked the colostrum and fed them a fresh cow diet (high-starch TMR). Three days after calving, the cows were transferred to a fresh barn until the end of the experiment. In the fresh bran, the cows were allowed access to the same high-starch TMR and water *ad libitum* during the experiment. Details on TMR are provided in Table S15.

The study included 211 healthy, multiparous, pregnant dairy cows. During the experiment, an experienced veterinarian conducted health assessments, including evaluations of body condition, rectal temperature, blood BHB concentration, and mental state. Table S16 presents the health assessment

criteria.

After excluding cows with abnormal conditions and those treated with medication during the experimental period to avoid interference with the generalizability of our results, 91 normal peripartum cows were included (Fig. 1). Rumen fluid, fecal, and blood samples were collected from these cows 21 days before calving (expected calving date) and on days 1, 3, 7, 14, and 21 after calving (Fig. 1). All samples were collected before the morning feeding on the designated sampling day. Rumen fluid samples were collected using a special rumen tube (Metal Systems, Kiryat Gat, Israel) designed based on the physiological structure of adult cows to ensure that the tube reached the ventral aspect of the rumen. The exterior metal of the rumen tube was polished to minimize damage to the esophagus and rumen. Fecal samples were obtained from the rectum of cows by research personnel wearing sterile long-arm gloves. Blood samples were collected from the caudal veins of cows in vacuum blood collection tubes containing EDTA. Rumen fluid and fecal samples were immediately transferred to 2 ml cryogenic tubes and stored in liquid nitrogen until subsequent bacterial diversity analysis. Blood samples were centrifuged at 3000 × *g* and 4°C for 15 min to obtain the plasma, which was then transferred to 2-ml cryogenic tubes and stored in liquid nitrogen for subsequent analysis of energy metabolism, liver function, and antioxidant indicators.

Microbial DNA extraction

Total genomic DNA was extracted using the DNeasy PowerSoil Pro Kit 47014 (Qiagen, Hilden, Germany), according to the manufacturer's instructions, and stored at -20°C for subsequent analysis. DNA quantity and quality were assessed using a NanoDrop NC2000 spectrophotometer (Thermo Fisher Scientific, Waltham, MA, USA) and agarose gel electrophoresis, respectively.

16S rRNA gene sequencing and upstream analysis of sequencing data

The 16S rRNA gene was amplified using universal primers (341F: 5′-CCTACGGGNGGCWGCAG-3′; 805R: 5′-GACTACHVGGGTATCTAATCC-3′) targeting the V3-V4 region, with 7-bp barcodes added for multiplex sequencing. Each PCR mixture contained 5 μl buffer (5×), 0.25 μl Fast pfu DNA Polymerase (5 U/μl), 2 μl (2.5 mM) dNTPs, 1 μl (10 μM) of each forward and reverse primer, 1 μl DNA

template, and 14.75 µl ddH2O. Thermal cycling steps involved an initial denaturation step at 98°C for 5 min, followed by 25 cycles of denaturation at 98°C for 30 s, annealing at 53°C for 30 s, and extension at 72°C for 45 s, with a final extension at 72°C for 5 min. The PCR amplicons were purified using Vazyme VAHTSTM DNA Clean Beads (Vazyme, Nanjing, China) and quantified using the Quant-iT PicoGreen dsDNA Assay Kit (Invitrogen, Carlsbad, CA, USA). After each quantification step, the amplicons were pooled in equal amounts, and paired-end 2×250-bp sequencing was performed using the Illumina NovaSeq platform with the NovaSeq 6000 SP Reagent Kit (500 cycles) at Shanghai Personal Biotechnology Co., Ltd (Shanghai, China).

Data quality control and analyses were performed using the QIIME2 pipeline with slight modifications according to official tutorials [54]. Briefly, raw sequence data were demultiplexed using the demux plugin, followed by primer cutting using the cutadapt plugin [55]. The sequences were then quality filtered, denoized, merged, and chimeras were removed using the DADA2 plugin [56]. Non-singleton amplicon sequence variants (ASVs) were aligned using mafft, and a phylogeny tree was constructed using fasttree2 [57, 58]. ASVs were taxonomically classified using the classify-sklearn naive Bayes taxonomy classifier in the feature-classifier plugin against the SILVA Release 132 database [59].

Metagenomic sequencing and upstream analysis of sequencing data

We conducted metagenomic analyses on samples randomly selected from 10 cows in the rumen and feces at three successional stages (N=60). The extracted total DNA was processed using the Illumina TruSeq Nano DNA LT Library Preparation Kit (Illumina, USA) to construct metagenomic shotgun sequencing libraries with an insert length of approximately 400 bp. Each library was sequenced on the Illumina NovaSeq™X Plus platform (Illumina, USA) and Personal Biotechnology Co., Ltd (Shanghai, China) using the PE150 strategy. For metagenomic data processing, Cutadapt (v1.2.1) was used to remove sequencing adapters from the raw reads [60]. Low-quality reads were trimmed using a sliding window algorithm in fastp (v0.23.2) [61]. Reads were aligned to the bovine genome using Minimap2 (v2.24-f1122) to remove host contamination [62]. Subsequently, Kaiju (v1.9.0) was used to classify metagenomic reads against the GTDB-derived database (v207) for each sample [63]. Reads assigned to Metazoa or Viridiplantae were excluded from downstream analysis. Megahit (v1.1.2) was used with the

meta-large preset parameters to assemble reads in each sample [64]. Contigs generated were clustered using the "easy-linclust" mode of Mmseqs2 (v15) with a sequence identity threshold of 0.95 and a 90% coverage of the shorter contigs [65]. Taxonomic classification of non-redundant contigs was conducted using the "taxonomy" mode of Mmseqs2 against the GTDB database, and contigs classified as belonging to Viridiplantae or Metazoa were removed from further analysis. Genes were predicted using Prodigal (v2.6.3) [66]. The CDS sequences from all samples were clustered using the "easy-cluster" mode of Mmseqs2, with a protein sequence identity threshold of 0.95 and 90% coverage of shorter sequences. Reads were then mapped to the predicted gene sequences using Minimap2, and featureCounts was used to calculate the number of reads aligned to each gene [67]. Abundance was expressed in TPM (Transcripts Per Million). The functional annotation of non-redundant genes was performed using the "search" mode of Mmseqs2 against the Metacyc database [68].

Downstream analysis of 16S rRNA gene sequencing data

After obtaining the ASV datasets of the rumen and fecal microbiome, they were processed using the "phyloseq" package in the R software (4.2.2), with a rarefaction depth set to the minimum sample sequence quantity. The ASV datasets were independently subsetted for individual analysis of the rumen and fecal microbiome. Conversely, for integrated analysis of the rumen and fecal microbiome, combined ASV datasets were aggregated. Data processing and downstream analyses were primarily conducted using R software. For examining the alpha diversity, ASV-level indices, including Chao1 richness and Shannon diversity index, were calculated using the "vegan" package in R [69, 70]. Beta diversity was explored using Bray–Curtis dissimilarity metrics to understand the structural variations in microbial communities across samples using the "vegan" package in R [71]. A Dirichlet multinomial mixture (DMM) model was applied at the genus level to cluster samples based on the microbial community structure [13, 72], with clusters determined according to the lowest Laplace approximation score [72]. This analysis was performed independently for rumen (RDMM) and fecal samples (FDMM). Models predicting microbial stages of the rumen and fecal microbiome were constructed using random forest algorithms with ASVs that showed more than 0.1% relative abundance, employing the "randomForest" package in R [73]. To reduce model overfitting, five 10-fold cross-validations were

conducted using 70% of samples for model building and 30% as the test set. The area under the receiver operating characteristic curve (AUC) was calculated using R. Phylogenetic bin-based null model analysis (ICAMP) was performed using a galaxy-based pipeline to assess the relative importance of deterministic and stochastic processes in bacterial community assembly [74]. The observed taxa were first grouped into bins ("boxes") based on their phylogenetic relationships, with the minimum number of taxa per bin as the default setting (bin.size.limit=24). Although the main function within icamp.big was used to calculate the within-bin β-nearest taxon index (βNTI), the modified Raup-Crick metric (RC) was used to evaluate the relative importance of different ecological processes within each bin. Procrustes analysis between the rumen and fecal ASVs was conducted using the "protest" function in the R package vegan [75]. To further determine the relationship between rumen and fecal microbiomes, fecal microbiome source tracking was conducted using the sourcetracker2 plugin (https://github.com/caporaso-lab/sourcetracker2), which can assess the proportion of fecal microbiome originating from rumen microbiome at the same time point and fecal microbiome at previous time points. Residuals between rumen or fecal samples and blood indicators were generated using the "Procrustes" function in the R package vegan.

Downstream analysis of metagenomic sequencing data

Species analyses were performed using read count data, while functional analyses were based on TPM datasets. Beta diversity at the species level, including bacteria, eukaryotes, and archaea, was assessed using Bray–Curtis dissimilarity. Network analysis focused on the top 500 most abundant genera, applying the SparCC method with an absolute R threshold of 0.6 [76]. Co-occurrence networks and node topology were evaluated to examine interspecies interactions and network centrality [77]. Functional beta diversity was assessed at the pathway level of the Metacyc database using Bray–Curtis dissimilarity. Differential species and pathways were identified with a prevalence over 50% and relative abundances of 0.1% and 0.01%, respectively.

Plasma parameters measurement

Glucose (GLU), triglycerides (TGs), aspartate aminotransferase (AST), and alanine aminotransferase

(ALT) concentrations in dairy plasma were determined using an automatic biochemistry analyzer (CLS880; Ze Cheng Biotechnology Co., Ltd., Jiangsu, China). Insulin (INS) levels were quantified via radioimmunoassays using a multitube counter (BFM-96; Zhongcheng Technology, Hefei, China). Direct field assessment of β-hydroxybutyrate (BHB) levels was performed in freshly collected blood samples using specific portable test strips (Nova Vet; Nova Biomedical Corporation, Waltham, MA, USA). Additionally, non-esterified fatty acid (NEFA), insulin-like growth factor 1 (IGF-1), total antioxidant capacity (T-AOC), and haptoglobin (HP) concentrations were measured via enzyme-linked immunosorbent assay using commercially available kits (Nanjing Jiancheng Bioengineering Institute, Nanjing, China). The RQUICKI-BHB (RBHB) index, calculated as 1/[log glucose (mg/dl)+log insulin (μU/ml)+log NEFA (mmol/l)+log BHB (mmol/l)], was used to evaluate insulin resistance in dairy cows based on a previously described method [78].

Statistical analysis and visualization

Differences in the microbial structure among groups were evaluated using permutational multivariate analysis of variance (PERMANOVA) with 999 permutations and visualized using principal coordinate analysis (PCoA) [79]. Genus diversity under different RDMM or FDMM conditions is represented via heatmaps using the ComplexHeatmap package in R [80]. Sample distributions at each peripartum time point and succession patterns for RDMM or FDMM are depicted using pie charts and Sankey diagrams (https://sankeymatic.com/build/), respectively. Transition dynamics between these states were analyzed via Markov chain models using the igraph and Markov chain packages in R [81], following the methodology by Xiao *et al*. [14]. Intraindividual and interindividual compositional variabilities were calculated according to the method by Olsson *et al*. [82]. Intraindividual compositional variability was defined as the median Bray–Curtis dissimilarity calculated between samples from a cow (i.e., 20 dissimilarity values were calculated for the six samples obtained from each cow). Interindividual compositional variability was defined as the median Bray–Curtis dissimilarity calculated for six samples from a cow against all other samples. Linear discriminant analysis effect size (LEfSe, LDA>4, p<0.05) was used to identify the dominant taxa across the groups [83]. Correlational analyses of individual factors and genera, with a cow as a random effect and false discovery rate adjustment, were performed

using MaAsLin2 [84]. Correlational analyses of genera, microbial functions, and blood metabolic indicators, with false discovery rate adjustment, were performed using Spearman in R. Based on the correlation coefficients, cooccurrence networks were generated and visualized using the “ggraph” package in R. Additional visualizations were created using ggplot2 in R. Comparative analyses of plasma parameter differences across groups were conducted by performing Kruskal–Wallis followed by Dunn’s post hoc tests using the “Kruskal.test” and “dunn.test” functions in R (dunn.test package). Statistical significance was set at $p < 0.05$.

Abbreviations

ASV, amplicon sequence variant; DL, dispersal limitation; DMM, Dirichlet multinomial mixture cluster; FDMM, DMM clusters for fecal microbiota; FDR, false discovery rate; HOS, homogeneous selection; LEfSe, Linear discriminant analysis effect size; RDMM, DMM clusters for rumen microbiota; PERMANOVA, permutational multivariate analysis of variance; GLU, glucose; TG, triglycerides, AST, aspartate aminotransferase; ALT, alanine aminotransferase; BHB, β-hydroxybutyrate; RBHB, RQUICKI-BHB; INS, Insulin; NEFA, non-esterified fatty acids; IGF-1; insulin-like growth factor 1; T-AOC; total antioxidant capacity; and HP, haptoglobin; ELISA, enzyme-linked immunosorbent assay; βNTI, β-nearest taxon index; modified RC, Raup-Crick metric; ICAMP, Phylogenetic bin-based null model analysis; TMR, total mixed ration

Competing Interests

The authors declare that they have no conflict of interest.

Acknowledgments

This study was supported by the National Natural Science Foundation of China (grant number 32130100).

Ethics Approval

The study design was approved by the Institutional Experimental Animal Care and Use Committee of

the Ministry of Agriculture and Rural Affairs of China and the Animal Care and Use Committee at China Agricultural University (approval number: AW01103202-1-31).

Author Contributions

Conceptualization: S.W., F.K., S.L., W.W.

Methodology: S.W., F.K., S.L.

Investigation: S.W., F.K., D.D., C.L.

Visualization: S.W., F.K.

Supervision: S.L., W.W.

Writing—original draft: S.W., F.K.

Writing—review & editing: S.W., F.K., Y.H., E.W., Z.C., Y.W., S.L., W.W

[1]Joachim von Braun KA, Louise O. Fresco, and Mohamed Hassan. Food systems: seven priorities to end hunger and protect the planet. Nature 2021; 597:28-30.
[2]Goff JP, Horst RL. Physiological changes at parturition and their relationship to metabolic disorders. J Dairy Sci 1997; 80:1260-1268.
[3]Bizelis J, Charismiadou M, Rodkais E. Metabolic changes during the perinatal period in dairy sheep in relation to level of nutrition and breed. II. Early lactation. J Anim Physiol An N 2000; 84:73-84.
[4]Bruckmaier R, Gross JJ. Lactational challenges in transition dairy cows. Anim Prod Sci 2017; 57:1471-1481.
[5]Esposito G, Irons PC, Webb EC, Chapwanya A. Interactions between negative energy balance, metabolic diseases, uterine health and immune response in transition dairy cows. Anim Reprod Sci 2014; 144:60-71.
[6]Cainzos JM, Andreu-Vazquez C, Guadagnini M, Rijpert-Duvivier A, Duffield T. A systematic review of the cost of ketosis in dairy cattle. J Dairy Sci 2022; 105:6175-6195.
[7]Cammack KM, Austin KJ, Lamberson WR, Conant GC, Cunningham HC. RUMINANT NUTRITION SYMPOSIUM: Tiny but mighty: the role of the rumen microbes in livestock production. J Anim Sci 2018; 96:752-770.
[8]O'Hara E, Neves AL, Song Y, Guan LL. The role of the gut microbiome in cattle production and health: driver or passenger? Annu rRev Anim Biosci. 2020; 8:199–220.
[9]Xue M, Sun H, Wu X, Guan LL, Liu J. Assessment of Rumen Microbiota from a Large Dairy Cattle Cohort Reveals the Pan and Core Bacteriomes Contributing to Varied Phenotypes. Appl Environ Microbiol 2018; 84.
[10]Shabat SK, Sasson G, Doron-Faigenboim A, Durman T, Yaacoby S, Berg Miller ME et al. Specific microbiome-dependent mechanisms underlie the energy harvest efficiency of ruminants. Isme j 2016; 10:2958-2972.
[11]Ley RE, Peterson DA, Gordon JI. Ecological and evolutionary forces shaping microbial diversity in the human intestine. Cell 2006; 124:837-848.

[12]Oliphant K, Parreira VR, Cochrane K, Allen-Vercoe E. Drivers of human gut microbial community assembly: coadaptation, determinism and stochasticity. ISME J 2019; 13:3080-3092.
[13]Stewart CJ, Ajami NJ, O'Brien JL, Hutchinson DS, Smith DP, Wong MC et al. Temporal development of the gut microbiome in early childhood from the TEDDY study. Nature 2018; 562:583-588.
[14]Xiao L, Wang J, Zheng J, Li X, Zhao F. Deterministic transition of enterotypes shapes the infant gut microbiome at an early age. Genome Biol 2021; 22:243.
[15]Fu Y, Gou W, Wu P, Lai Y, Liang X, Zhang K et al. Landscape of the gut mycobiome dynamics during pregnancy and its relationship with host metabolism and pregnancy health. Gut 2024; 73, 1302-12.
[16]Feng Y, Zhang M, Liu Y, Yang X, Wei F, Jin X et al. Quantitative microbiome profiling reveals the developmental trajectory of the chicken gut microbiota and its connection to host metabolism. iMeta 2023; 2:e105.
[17]Wang X, Tsai T, Deng F, Wei X, Chai J, Knapp J et al. Longitudinal investigation of the swine gut microbiome from birth to market reveals stage and growth performance associated bacteria. Microbiome 2019; 7:109.
[18]Chai J, Zhuang Y, Cui K, Bi Y, Zhang N. Metagenomics reveals the temporal dynamics of the rumen resistome and microbiome in goat kids. Microbiome 2024; 12:14.
[19]Yan X, Si H, Zhu Y, Li S, Han Y, Liu H et al. Integrated multi-omics of the gastrointestinal microbiome and ruminant host reveals metabolic adaptation underlying early life development. Microbiome 2022; 10:222.
[20]Malmuthuge N, Liang G, Guan LL. Regulation of rumen development in neonatal ruminants through microbial metagenomes and host transcriptomes. Genome Biol 2019; 20:172.
[21]Tröscher-Mußotter J, Saenz JS, Grindler S, Meyer J, Kononov SU, Mezger B et al. Microbiome clusters disclose physiologic variances in dairy cows challenged by calving and lipopolysaccharides. mSystems 2021; 6:10–128.
[22]Zhu Z, Noel SJ, Difford GF, Al-Soud WA, Brejnrod A, Sorensen SJ et al. Community structure of the metabolically active rumen bacterial and archaeal communities of dairy cows over the transition period. PLoS One 2017; 12:e0187858.

[23]Bach A, López-García A, González-Recio O, Elcoso G, Fàbregas F, Chaucheyras-Durand F et al. Changes in the rumen and colon microbiota and effects of live yeast dietary supplementation during the transition from the dry period to lactation of dairy cows. J Dairy Sci 2019; 102:6180-6198.

[24]Zhu SL, Gu FF, Tang YF, Liu XH, Jia MH, Valencak TG et al. Dynamic fecal microenvironment properties enable predictions and understanding of peripartum blood oxidative status and nonesterified fatty acids in dairy cows. J Dairy Sci 2024; 107:573-592.

[25]Luo Z, Du Z, Huang Y, Zhou T, Wu D, Yao X et al. Alterations in the gut microbiota and its metabolites contribute to metabolic maladaptation in dairy cows during the development of hyperketonemia. mSystems 2024; 9:e0002324.

[26]Arshad MA, Hassan F-u, Rehman MS, Huws SA, Cheng Y, Din AU. Gut microbiome colonization and development in neonatal ruminants: Strategies, prospects, and opportunities. Anim Nutr 2021; 7:883-895.

[27]Xu Q, Qiao Q, Gao Y, Hou J, Hu M, Du Y et al. Gut microbiota and their role in health and metabolic disease of dairy cow. Front Nutr 2021; 8:701511.

[28]Hernandez DJ, David AS, Menges ES, Searcy CA, Afkhami ME. Environmental stress destabilizes microbial networks. ISME J 2021; 15:1722-1734.

[29]Sharma A, Richardson M, Cralle L, Stamper CE, Maestre JP, Stearns-Yoder KA et al. Longitudinal homogenization of the microbiome between both occupants and the built environment in a cohort of United States Air Force Cadets. Microbiome 2019; 7:1-17.

[30]Wright RJ, Gibson MI, Christie-Oleza JA. Understanding microbial community dynamics to improve optimal microbiome selection. Microbiome 2019; 7:1-14.

[31]George AB, Korolev KS. Ecological landscapes guide the assembly of optimal microbial communities. Plos Comput Biol 2023; 19:e1010570.

[32]Li WJ, Ma T, Zhang NF, Deng KD, Diao QY. Dietary fat supplement affected energy and nitrogen metabolism efficiency and shifted rumen fermentation toward glucogenic propionate production via enrichment of Succiniclasticum in male twin lambs1. J Integr Agr 2023.

[33]Wang D, Chen L, Tang G, Yu J, Chen J, Li Z et al. Multi-omics revealed the long-term effect of

ruminal keystone bacteria and the microbial metabolome on lactation performance in adult dairy goats. Microbiome 2023; 11:215.

[34]Li L, Batt SM, Wannemuehler M, Dispirito A, Beitz DC. Effect of feeding of a cholesterol-reducing bacterium, Eubacterium coprostanoligenes, to germ-free mice. Lab Anim Sci 1998; 48:253-255.

[35]Li X, Jensen RL, Højberg O, Canibe N, Jensen BB. Olsenella scatoligenes sp. nov., a 3-methylindole- (skatole) and 4-methylphenol- (p-cresol) producing bacterium isolated from pig faeces. Int J Syst Evol Microbiol 2015; 65:1227-1233.

[36]Fan Q, Wanapat M, Yan T, Hou F. Altitude influences microbial diversity and herbage fermentation in the rumen of yaks. BMC Microbiol 2020; 20:370.

[37]Xiong Y, Wang X, Li X, Guo L, Yang F, Ni K. Exploring the rumen microbiota of Hu lambs in response to diet with paper mulberry. Appl Microbiol Biotechnol 2023; 107:4961-4971.

[38]Sasajima N, Ogasawara T, Takemura N, Fujiwara R, Watanabe J, Sonoyama K. Role of intestinal Bifidobacterium pseudolongum in dietary fructo-oligosaccharide inhibition of 2, 4-dinitrofluorobenzene-induced contact hypersensitivity in mice. Brit J Nutr 2010; 103:539-548.

[39]Sun D, Bian G, Zhang K, Liu N, Yin Y, Hou Y et al. Early-life ruminal microbiome-derived indole-3-carboxaldehyde and prostaglandin D2 are effective promoters of rumen development. Genome Biology 2024; 25:64.

[40]Shen H, Lu Z, Xu Z, Chen Z, Shen Z. Associations among dietary non-fiber carbohydrate, ruminal microbiota and epithelium G-protein-coupled receptor, and histone deacetylase regulations in goats. Microbiome 2017; 5:1-12.

[41]Furman O, Shenhav L, Sasson G, Kokou F, Honig H, Jacoby S et al. Stochasticity constrained by deterministic effects of diet and age drive rumen microbiome assembly dynamics. Nat Commun. 2020; 11:1904.

[42]Roche JR, Friggens NC, Kay JK, Fisher MW, Stafford KJ, Berry DP. Invited review: Body condition score and its association with dairy cow productivity, health, and welfare. J Dairy Sci 2009; 92:5769-5801.

[43]Li F, Li C, Chen Y, Liu J, Zhang C, Irving B et al. Host genetics influence the rumen microbiota and heritable rumen microbial features associate with feed efficiency in cattle. Microbiome 2019;

7:1-17.
[44]Wang W, Zhang Y, Zhang X, Li C, Yuan L, Zhang D et al. Heritability and recursive influence of host genetics on the rumen microbiota drive body weight variance in male Hu sheep lambs. Microbiome 2023; 11:197.
[45]Chen L, Wang D, Garmaeva S, Kurilshikov A, Vich Vila A, Gacesa R et al. The long-term genetic stability and individual specificity of the human gut microbiome. Cell 2021; 184:2302-2315.e2312.
[46]Yang H, Wu J, Huang X, Zhou Y, Zhang Y, Liu M et al. ABO genotype alters the gut microbiota by regulating GalNAc levels in pigs. Nature 2022; 606:358-367.
[47]Nicola I, Chupin H, Roy J-P, Buczinski S, Fauteux V, Picard-Hagen N et al. Association between prepartum nonesterified fatty acid serum concentrations and postpartum diseases in dairy cows. J Dairy Sci 2022; 105:9098-9106.
[48]LeBlanc S. Monitoring metabolic health of dairy cattle in the transition period. J Reprod Develop 2010; 56:S29-S35.
[49]Cani PD, Delzenne NM. The role of the gut microbiota in energy metabolism and metabolic disease. Curr Pharm Design 2009; 15:1546-1558.
[50]Zhu L, Baker SS, Gill C, Liu W, Alkhouri R, Baker RD et al. Characterization of gut microbiomes in nonalcoholic steatohepatitis (NASH) patients: a connection between endogenous alcohol and NASH. Hepatology 2013; 57:601-609.
[51]Wu D, Liu L, Jiao N, Zhang Y, Yang L, Tian C et al. Targeting keystone species helps restore the dysbiosis of butyrate-producing bacteria in nonalcoholic fatty liver disease. iMeta 2022; 1:e61.
[52]Parker BJ, Wearsch PA, Veloo ACM, Rodriguez-Palacios A. The Genus Alistipes: Gut Bacteria With Emerging Implications to Inflammation, Cancer, and Mental Health. Front Immunol 2020; 11:906.
[53] Rangan P, Mondino A. Microbial short-chain fatty acids: a strategy to tune adoptive T cell therapy. J Immunotherap Cancer. 2022; 10.
[54]Bolyen E, Rideout JR, Dillon MR, Bokulich NA, Abnet CC, Al-Ghalith GA et al. Reproducible, interactive, scalable and extensible microbiome data science using QIIME 2. Nat Biotechnol

2019; 37:852-857.

[55]Kechin A, Boyarskikh U, Kel A, Filipenko M. cutPrimers: A New Tool for Accurate Cutting of Primers from Reads of Targeted Next Generation Sequencing. J Comput Biol 2017; 24:1138-1143.

[56]Callahan BJ, McMurdie PJ, Rosen MJ, Han AW, Johnson AJ, Holmes SP. DADA2: High-resolution sample inference from Illumina amplicon data. Nat Methods 2016; 13:581-583.

[57]Katoh K, Misawa K, Kuma K, Miyata T. MAFFT: a novel method for rapid multiple sequence alignment based on fast Fourier transform. Nucleic Acids Res 2002; 30:3059-3066.

[58]Price MN, Dehal PS, Arkin AP. FastTree 2--approximately maximum-likelihood trees for large alignments. PLoS One 2010; 5:e9490.

[59]Bokulich NA, Kaehler BD, Rideout JR, Dillon M, Bolyen E, Knight R et al. Optimizing taxonomic classification of marker-gene amplicon sequences with QIIME 2's q2-feature-classifier plugin. Microbiome 2018; 6:90.

[60]Martin M. Cutadapt removes adapter sequences from high-throughput sequencing reads. EMBnet J 2011; 17:10-12.

[61]Chen S, Zhou Y, Chen Y, Gu J. fastp: an ultra-fast all-in-one FASTQ preprocessor. Bioinformatics 2018; 34:i884-i890.

[62]Li H. Minimap2: pairwise alignment for nucleotide sequences. Bioinformatics 2018; 34:3094-3100.

[63]Menzel P, Ng KL, Krogh A. Fast and sensitive taxonomic classification for metagenomics with Kaiju. Nat Commun 2016; 7:11257.

[64]Li D, Liu C-M, Luo R, Sadakane K, Lam T-W. MEGAHIT: an ultra-fast single-node solution for large and complex metagenomics assembly via succinct de Bruijn graph. Bioinformatics 2015; 31:1674-1676.

[65]Steinegger M, Söding J. MMseqs2 enables sensitive protein sequence searching for the analysis of massive data sets. Nat Biotechnol 2017; 35:1026-1028.

[66]Hyatt D, Chen GL, Locascio PF, Land ML, Larimer FW, Hauser LJ. Prodigal: prokaryotic gene recognition and translation initiation site identification. BMC Bioinformatics 2010; 11:119.

[67]Liao Y, Smyth GK, Shi W. featureCounts: an efficient general purpose program for assigning

sequence reads to genomic features. Bioinformatics 2014; 30:923-930.
[68]Caspi R, Billington R, Keseler IM, Kothari A, Krummenacker M, Midford PE et al. The MetaCyc database of metabolic pathways and enzymes - a 2019 update. Nucleic Acids Res 2020; 48:D445-d453.
[69]Chao A. Nonparametric Estimation of the Number of Classes in a Population. Scand J Stat 1984; 11:265-270.
[70]Shannon CE. A mathematical theory of communication. Bell Syst Tech J 1948; 27:379-423.
[71]Bray JR, Curtis JT. An ordination of the upland forest communities of southern Wisconsin. Ecological monographs 1957; 27:326-349.
[72]Holmes I, Harris K, Quince C. Dirichlet multinomial mixtures: generative models for microbial metagenomics. PloS one 2012; 7:e30126.
[73]Rigatti SJ. Random forest. Journal of Insurance Medicine 2017; 47:31-39.
[74]Ning D, Yuan M, Wu L, Zhang Y, Guo X, Zhou X et al. A quantitative framework reveals ecological drivers of grassland microbial community assembly in response to warming. Nat Commun 2020; 11:4717.
[75]Andreella A, Finos L. Procrustes Analysis for High-Dimensional Data. Psychometrika 2022; 87:1422-1438.
[76]Friedman J, Alm EJ. Inferring correlation networks from genomic survey data. PLoS Comput Biol 2012; 8:e1002687.
[77]Csardi G, Nepusz T. The igraph software. Complex syst 2006; 1695:1-9.
[78]Cai J, Zhao FQ, Liu JX, Wang DM. Local Mammary Glucose Supply Regulates Availability and Intracellular Metabolic Pathways of Glucose in the Mammary Gland of Lactating Dairy Goats Under Malnutrition of Energy. Front Physiol 2018; 9:1467.
[79]McArdle BH, Anderson MJ. Fitting multivariate models to community data: a comment on distance-based redundancy analysis. Ecology 2001; 82:290-297.
[80]Gu Z. Complex heatmap visualization. Imeta 2022; 1:e43.
[81]Han W-S, Lee J, Pham M-D, Yu JX. iGraph: a framework for comparisons of disk-based graph indexing techniques. Proc VLDB Endow 2010; 3:449-459.

[82]Olsson LM, Boulund F, Nilsson S, Khan MT, Gummesson A, Fagerberg L et al. Dynamics of the normal gut microbiota: a longitudinal one-year population study in Sweden. Cell Host Microbe 2022; 30:726-739. e723.
[83]Segata N, Izard J, Waldron L, Gevers D, Miropolsky L, Garrett WS et al. Metagenomic biomarker discovery and explanation. Genome biology 2011; 12:1-18.
[84]Mallick H, Rahnavard A, McIver LJ, Ma S, Zhang Y, Nguyen LH et al. Multivariable association discovery in population-scale meta-omics studies. PLoS Comput Biol 2021; 17:e1009442.

Figures

Fig. 1. Profiling gut microbiome changes in periparturient dairy cows and their connection to host metabolism: Workflow. BHB: β-hydroxybutyrate.

Fig. 2. Classification of the rumen and fecal microbiome in periparturient dairy cows based on microbial community clusters. Boxplots showing intra- and interindividual Bray–Curtis dissimilarity in microbiome profiles: (a) Rumen and (f) feces. The PCoA plot-based Bray–Curtis dissimilarity of microbiome profiles across different DMMs: (b) rumen and (g) feces. DMM represents a microbial community cluster. Ring charts showing the distribution of DMMs, with the numbers in the middle of the ring charts representing the sampling time points: (c) rumen and (h) feces. LEfSe reveals the key genera of different DMMs: (d) rumen and (i) feces. Temporal changes of Shannon index in each DMM: (e) rumen and (j) feces. DMM, Microbial cluster.

Fig. 3. Temporal dynamics of ruminal DMMs in periparturient dairy cows. (a) The Sanky diagram shows the transition of ruminal DMMs across six sampling time points. (b) Markov chain with subject-independent transition probabilities among ruminal DMMs, in which arrow weights are proportional to the maximum likelihood estimate of the transition probabilities among different states. Ruminal DMMs in different colored boxes are in different stages of microbial succession. The numbers represent the conversion rates across different ruminal DMMs. The numbers represent the conversion rates across

different ruminal DMMs. (c) Receiver operating characteristic curve demonstrating the accuracy of the classification model for the successional stages in the rumen microbiome of periparturient dairy cows. DMM, Microbial cluster.

Fig. 4. Temporal dynamics of fecal DMMs in periparturient dairy cows. (a) The Sanky diagram shows the transition of fecal DMMs across six sampling time points. (b) Markov chain with subject-independent transition probabilities among fecal DMMs, in which arrow weights are proportional to the maximum likelihood estimate of the transition probabilities among different states. Fecal DMMs in different colored boxes are in different stages of microbial succession. The numbers represent the conversion rates across different fecal DMMs. The numbers represent the conversion rates across different fecal DMMs. (c) Receiver operating characteristic curve demonstrating the accuracy of the classification model for the successional stages in the fecal microbiome of periparturient dairy cows. DMM, Microbial cluster.

Fig. 5. Ecological assembly mechanism of rumen microbiome in periparturient dairy cows. (a) Relative importance of HOS and DL processes in the rumen microbiome at different successional stages in periparturient cows. (b) Differences in ecological processes among different phylogenetic groups (the relative abundance of the top20 Bins) in the rumen. The different colors of the inner and outer circles represent the phylum affiliations of the Bins and the ecological processes driven by the Bins, respectively. (c) Stacked plot showing the relative abundance of the top20 Bins in different rumen stages. (d) Relative contribution of the top20 Bins to DL and HOS processes in different rumen stages. (e) The relative abundance of representative genera of Bins contributing to succession at different rumen stages. HOS, Homogeneous selection; DL, Dispersal limitation; DMM, Microbial cluster.

Fig. 6. Ecological assembly mechanism of the fecal microbiome in periparturient dairy cows. (a) Relative importance of HOS and DL processes in the fecal microbiome at different successional stages in periparturient cows. (b) Differences in ecological processes among different phylogenetic groups (the relative abundance of the top20 Bins) in the feces. The different colors of the inner and outer circles

represent the phylum affiliations of the Bins and the ecological processes driven by the Bins, respectively. (c) Stacked plot showing the relative abundance of the top20 Bins in different fecal DMMs. (d) Relative contribution of the top20 Bins to DL and HOS processes in different fecal DMMs. (e) The relative abundance of representative genera of Bins contributing to succession at different fecal stages. HOS, Homogeneous selection; DL, Dispersal limitation; DMM, Microbial cluster; RS1, 2, 3, rumen succession stages 1, 2, 3; FS1, 2, 3, fecal succession stages 1, 2, 3.

Fig. 7. Significance and explained variance of 18 microbiome covariates modelled by EnvFit across all data types. Horizontal bars show the amount of variance (r^2) explained by each covariate in the model as determined by EnvFit. Significant covariates ($p< 0.05$) are represented in bold font. P-A: Predelivery - Actual; BW: Birth weight; DIM: Days in milk; NM: Number of matings; CD: Calving to days; MY: Milk yeild; RPH: Ruminal pH; BCS: Body Condition Score; RT: Rectal temperature; FPH: Fecal pH; CP: Crude protein; ADF: Acid detergent fiber; NDF: Neutral detergent fiber

Fig. 8. Analysis of the relationship between the rumen and fecal microbiome as well as effect of succession types on host metabolic phenotype in periparturient cows. (a) Procrustes analysis of the correlation between ruminal and fecal microbiome based on the Bray–Curtis dissimilarity of ASVs (M^2=0.79, p<0.01, 999 permutations). (b) Source tracking of the fecal microbiome in periparturient cows. Fecal samples from the former sampling time point and rumen samples from the same sampling time point were considered potential sources of feces at this time point in the same cow. (c) Residuals showing the difference in the microbe–host association from rumen and feces with relative abundance. Microbial succession types and statistics: (d) rumen and (f) feces. Boxplot showing the significance test of effect of succession types on host metabolic phenotype (only significant combinations are shown): (e) rumen and (g) feces. RS1, 2, 3, rumen succession stages 1, 2, 3; FS1, 2, 3, fecal succession stages 1, 2, 3; S1-S1, the microbial stage of the individual transitions from S1 to S1; RBHB, RQUICKI-BHB index; BHB, β-hydroxybutyrate; HP, haptoglobin; T-AOC, total antioxidant capacity; AST, aspartate aminotransferase; ALT, alanine aminotransferase; IGF-1, insulin-like growth factor 1; NEFA, non-esterified fatty acids; TG, triglycerides; INS, Insulin

Fig. 9. Analysis of microbiome–host interactions in periparturient cows. (a) The PCoA based on species-level Bray–Curtis dissimilarity shows differences in rumen and fecal microbiome profiles across succession stages. (b) The co-occurrence network diagram displays the SparCC interaction relationships of the top 500 abundant genera at different rumen of fecal succession stages ($R > 0.6$; $p < 0.05$). (c) Topological analysis of the co-occurrence network. (d) The PCoA analysis based on Bray–Curtis dissimilarity of pathways reveals distinct microbial functional profiles in the rumen and feces across different successional stages. Heatmap showing the significantly different metabolic pathways (normalized) of the microbiome at different succession stages in periparturient cows: (e) rumen and (f) feces. Part of mediation linkages among the species, pathways, and metabolic phenotype: (e) rumen and (f) feces. RS1, 2, 3, rumen succession stages 1, 2, 3; FS1, 2, 3, fecal succession stages 1, 2, 3; NEFA, non-esterified fatty acids; TG, triglycerides; INS, Insulin

Supplementary Figures

Figs. S1 to S4:

Fig. S1. Dynamics and diversity of rumen microbiota during the periparturient period. (a) PCoA plot-based Bray–Curtis dissimilarity showing rumen microbiota profiles across different sampling time points. (b) Dynamic changes in the α-diversity of rumen microbiota at different periods during the perinatal period. (c) Plots showing a comparison of the five bacterial phyla with the highest relative abundance in the rumens of perinatal cows. (d) Laplace approximation scores for ruminal DMM. (e) The top 20 taxa that contributed the most to the accuracy of the rumen DMM are shown in the order of importance. (f) Heatmap showing the relative abundance of the top 20 taxa (normalised in each taxon) in each rumen sample.

Fig. S2. Dynamics and diversity of fecal microbiota during the periparturient period. (a) PCoA plot-based Bray–Curtis dissimilarity showing fecal microbiota profiles across different sampling time points. (b) Dynamic changes in the α-diversity of fecal microbiota at different time points during the perinatal period. (c) Plots showing a comparison of the five bacterial phyla with the highest relative abundance in the fecal microbiota of perinatal cows. (d) Laplace approximation scores for fecal DMM. (e) The top 20

taxa that contributed the most to the accuracy of the fecal DMM are shown in the order of importance. (f) Heatmap showing the relative abundance of the top 20 taxa (normalised in all taxa) in each fecal sample.

Fig. S3. Cross-validation of ASVs contributing to the random forest model. (a) Rumen. (b) Feces. ASV, Amplicon sequence variant

Fig. S4. Relative importance of different ecological processes in the microbiota composition of periparturient dairy cows: (a) Rumen. (b) Feces.

Supplementary Tables

Tables S1 to S20:

Table S1. Details of transformation of ruminal DMMs at different time points.

Table S2. Details of transformation of fecal DMMs at different time points.

Table S3. Details of ruminal Bins in ICAMP assembly.

Table S4. Details of fecal Bins in ICAMP assembly.

Table S5. MaAsLin2 method was used to investigate the relationship between individual factors and key genera driving succession in dairy cows.

Table S6. Analysis of the contribution of rumen and fecal microbiota to blood metabolism.

Table S7. Node analysis of co-occurrence network in RS1 stage.

Table S8. Node analysis of co-occurrence network in RS2 stage.

Table S9. Node analysis of co-occurrence network in RS3 stage.

Table S10. Node analysis of co-occurrence network in FS1 stage.

Table S11. Node analysis of co-occurrence network in FS2 stage.

Table S12. Node analysis of co-occurrence network in FS3 stage.

Table S13. Mediation analysis of ruminal different species, ruminal different pathways, and metabolic phenotype.

Table S14. Mediation analysis of fecal different species, fecal different pathways, and metabolic phenotype.

Table S15. Feed ingredients and nutrient composition of diets.

Table S16. Summary of reasons for elimination.

Table S17. Quality control results of 16S rRNA gene sequencing.

Table S18. Annotation details of the amplicon sequence variants in Sliva database in this study.

Table S19. Annotation details of the amplicon sequence variants in Greengenes2 database in this study.

Table S20. Quality control results of metagenomic sequencing.

 Click here to access/download;Figure;FIG.1.docx

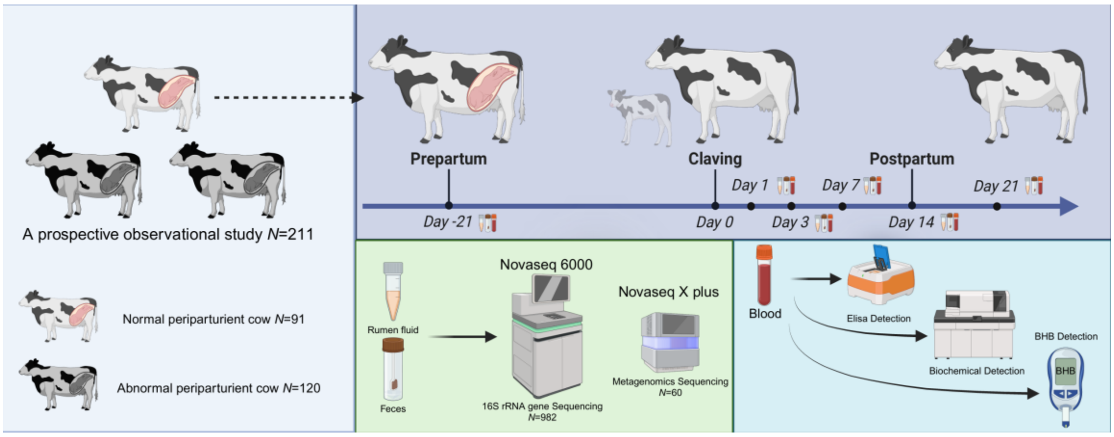

Figure2

Click here to access/download;Figure;Figuer 2.tif

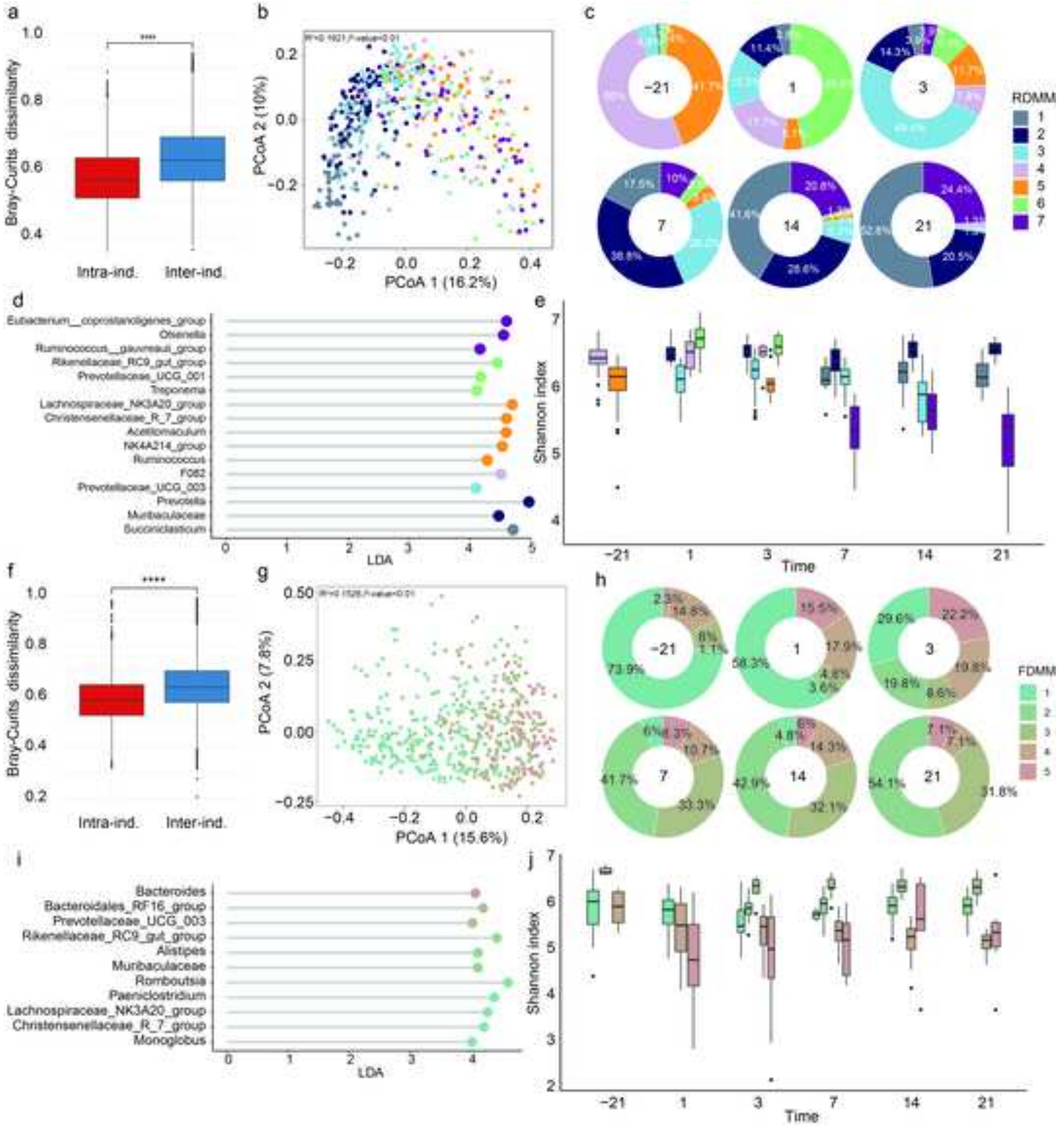

Figure3 Click here to access/download;Figure;Figure 3 .tif

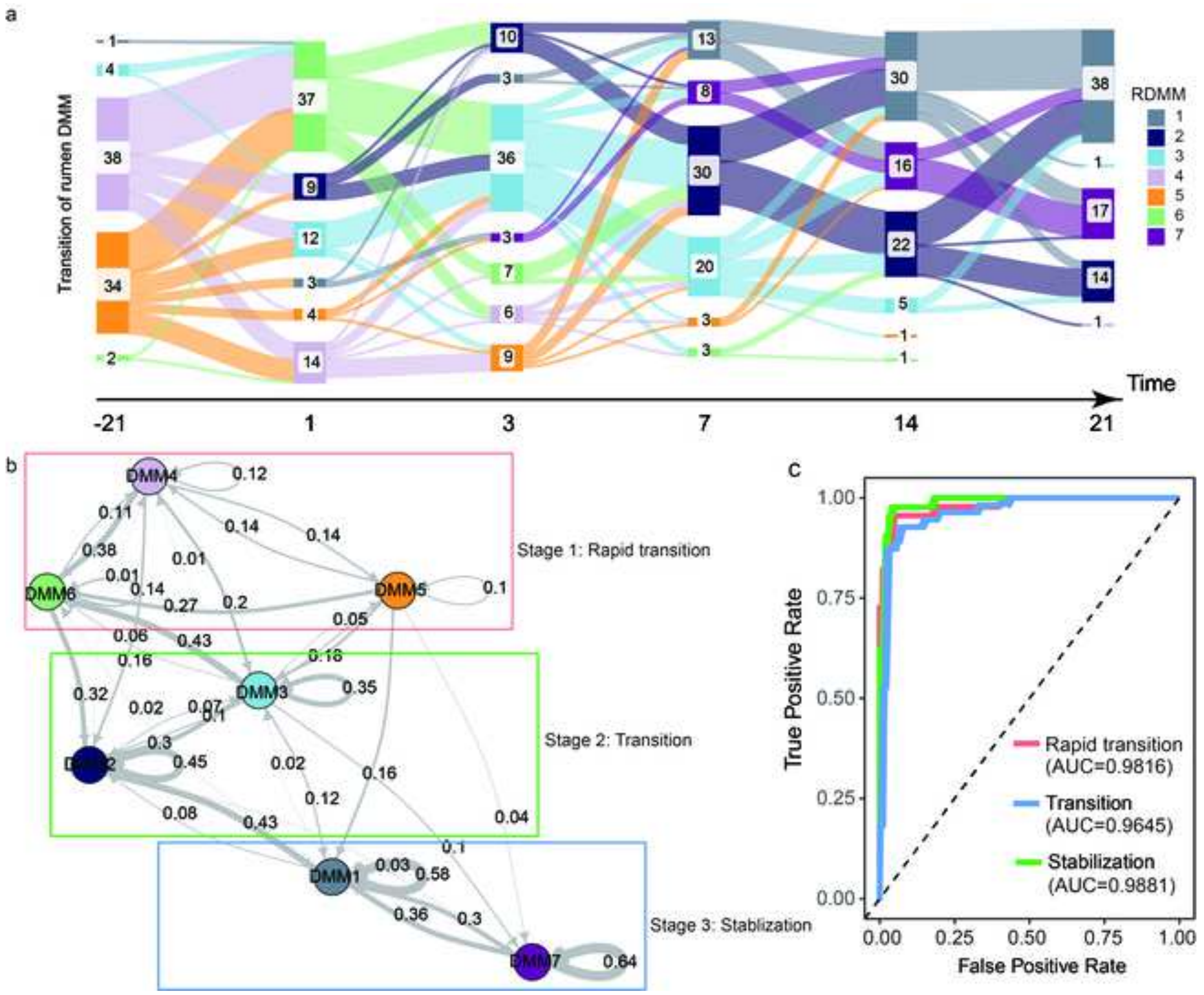

Figure4

Click here to access/download;Figure;Figure 4.tif

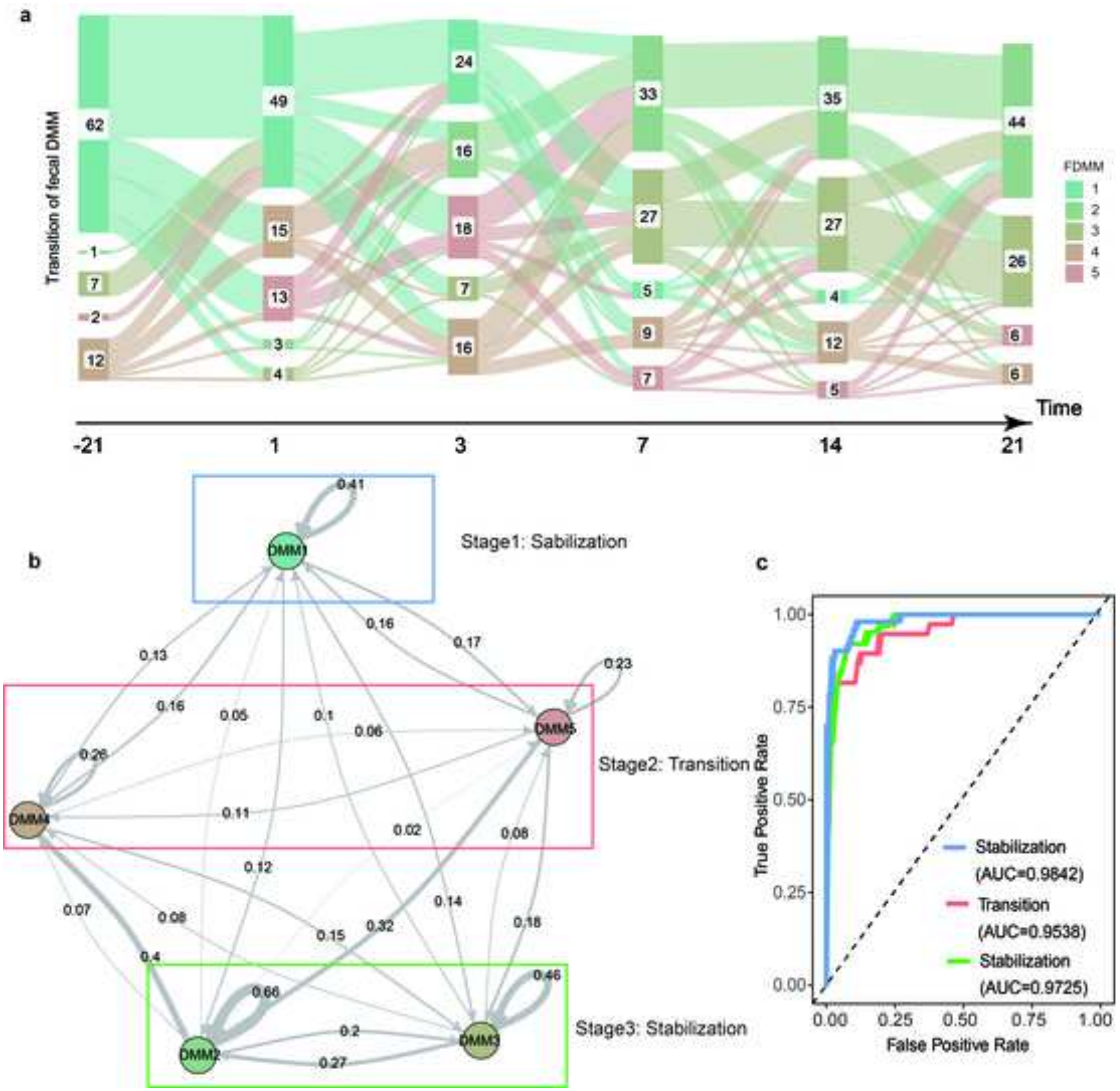

Figure5

Click here to access/download;Figure;Figure 5.tif

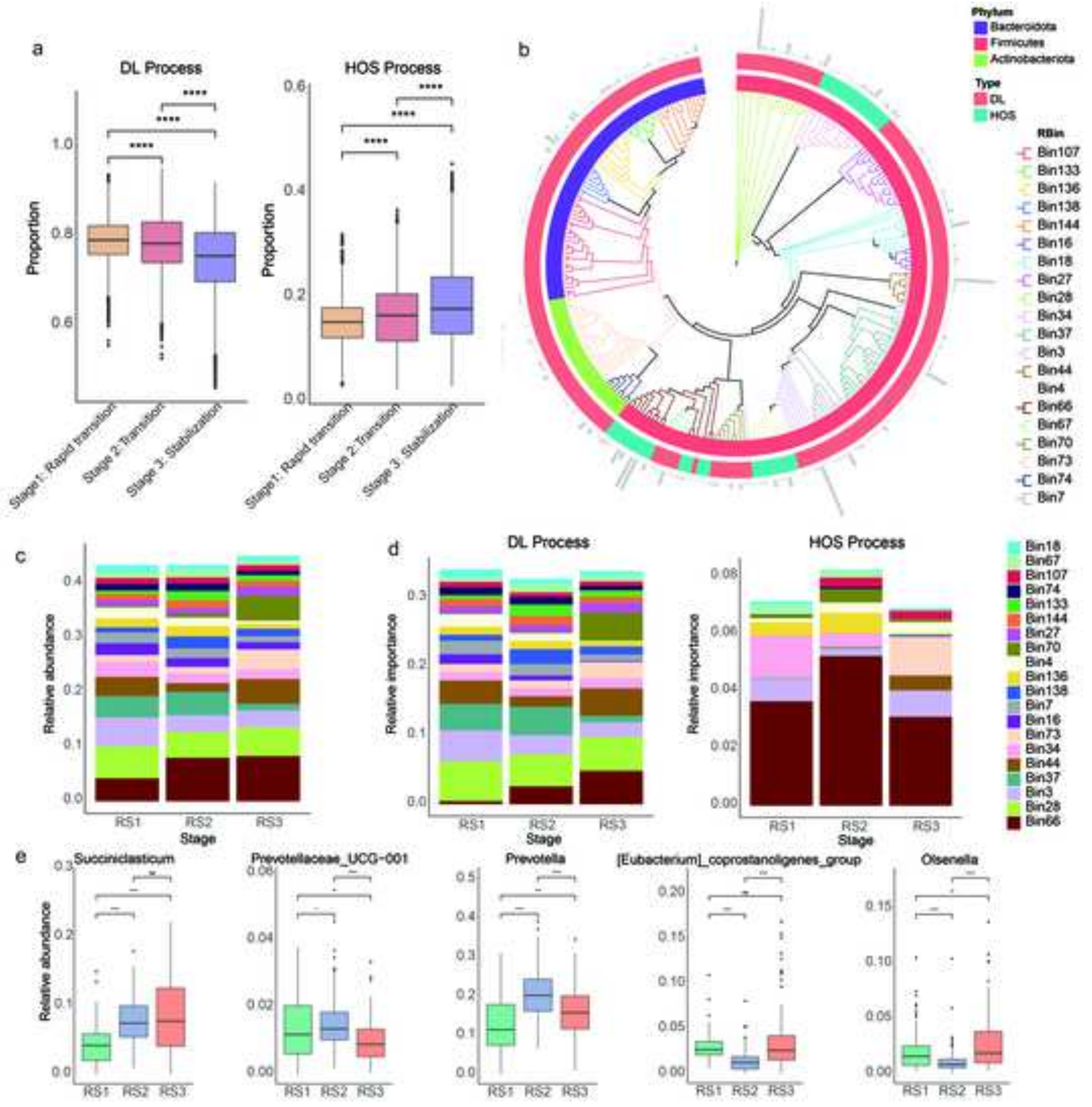

Figure6 Click here to access/download;Figure;Figure 6.tif

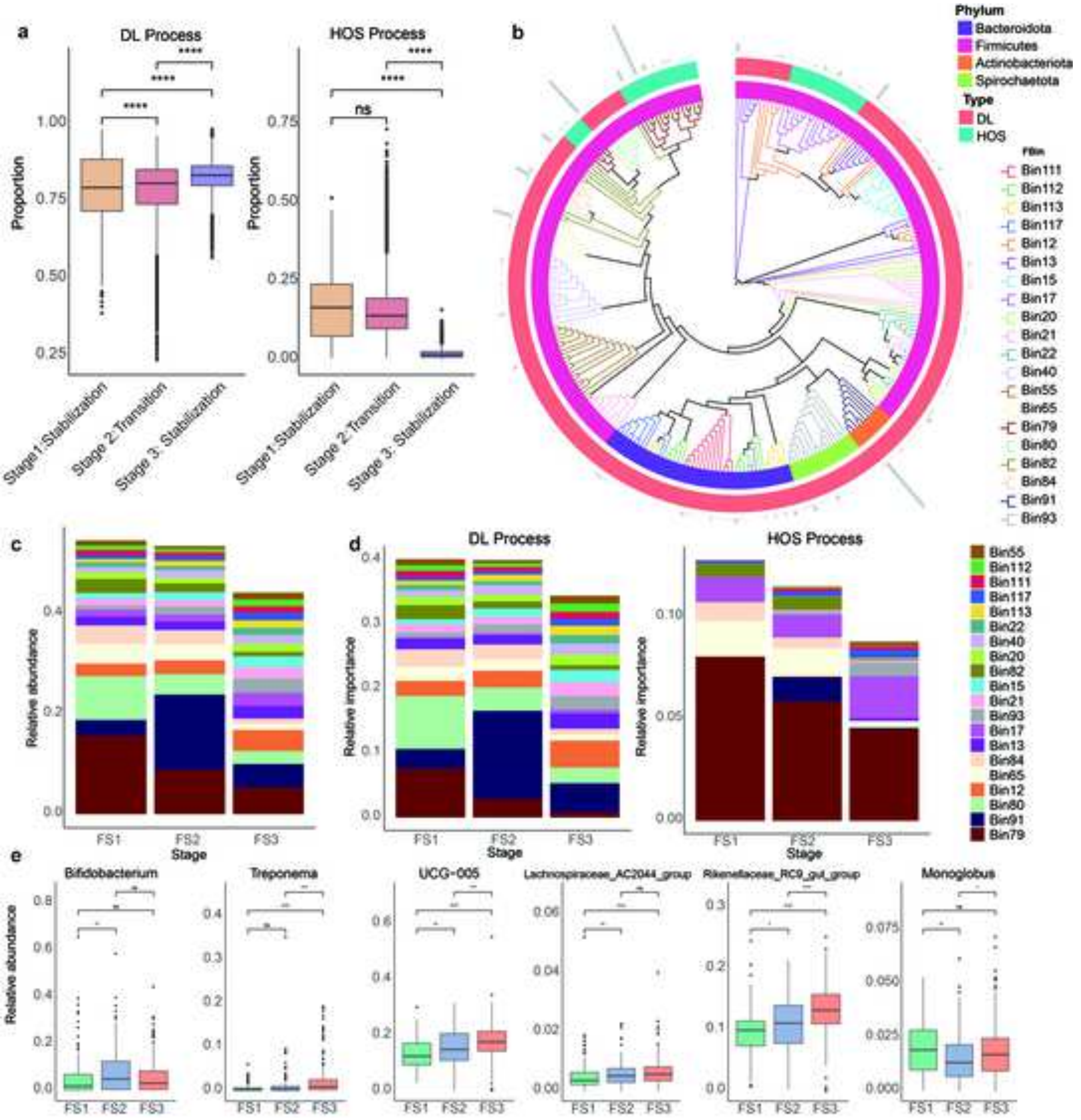

Figure7

Click here to access/download;Figure;Figure 7 .tif

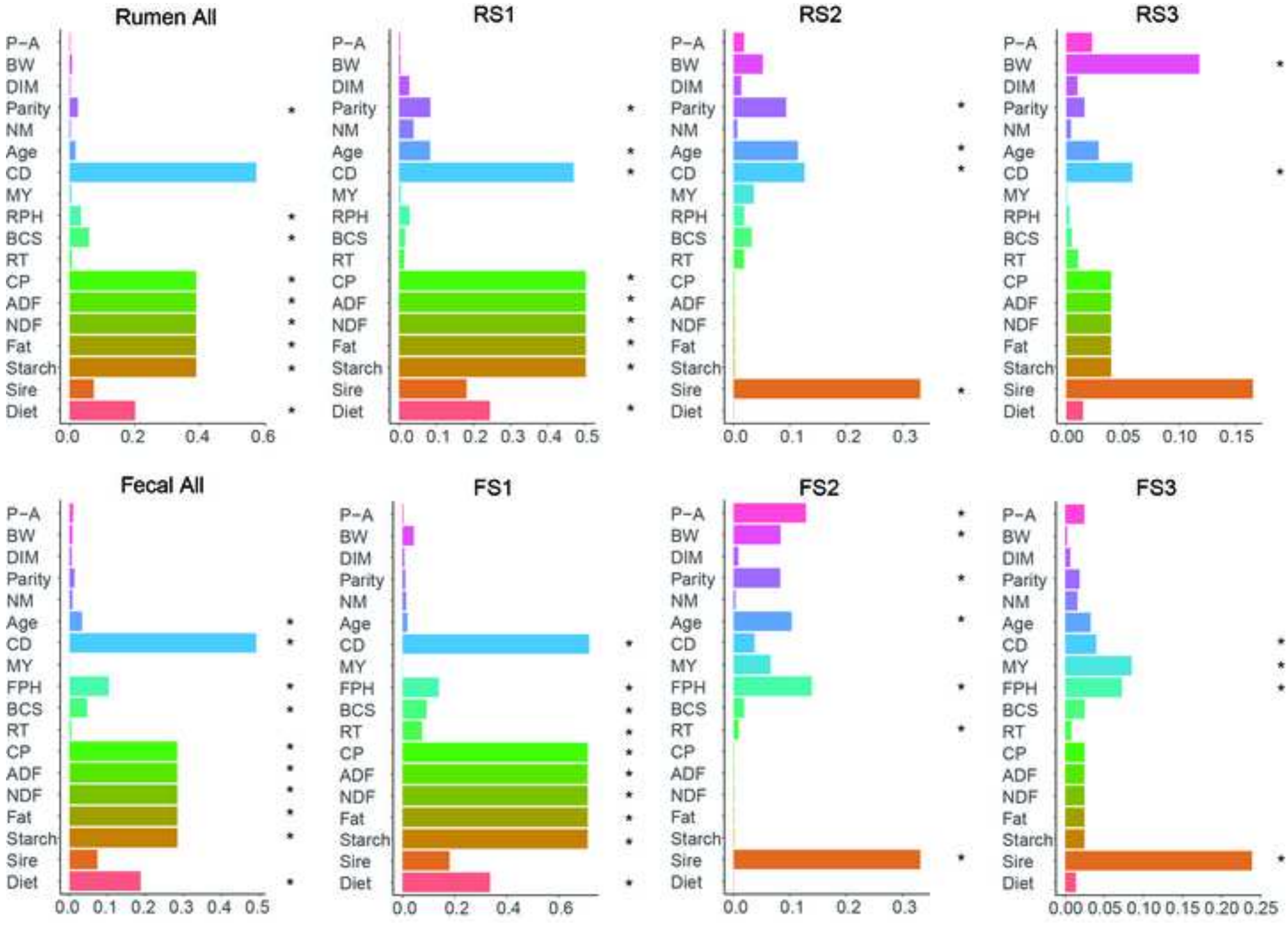

Figure8

Click here to access/download;Figure;Figure 8.tif

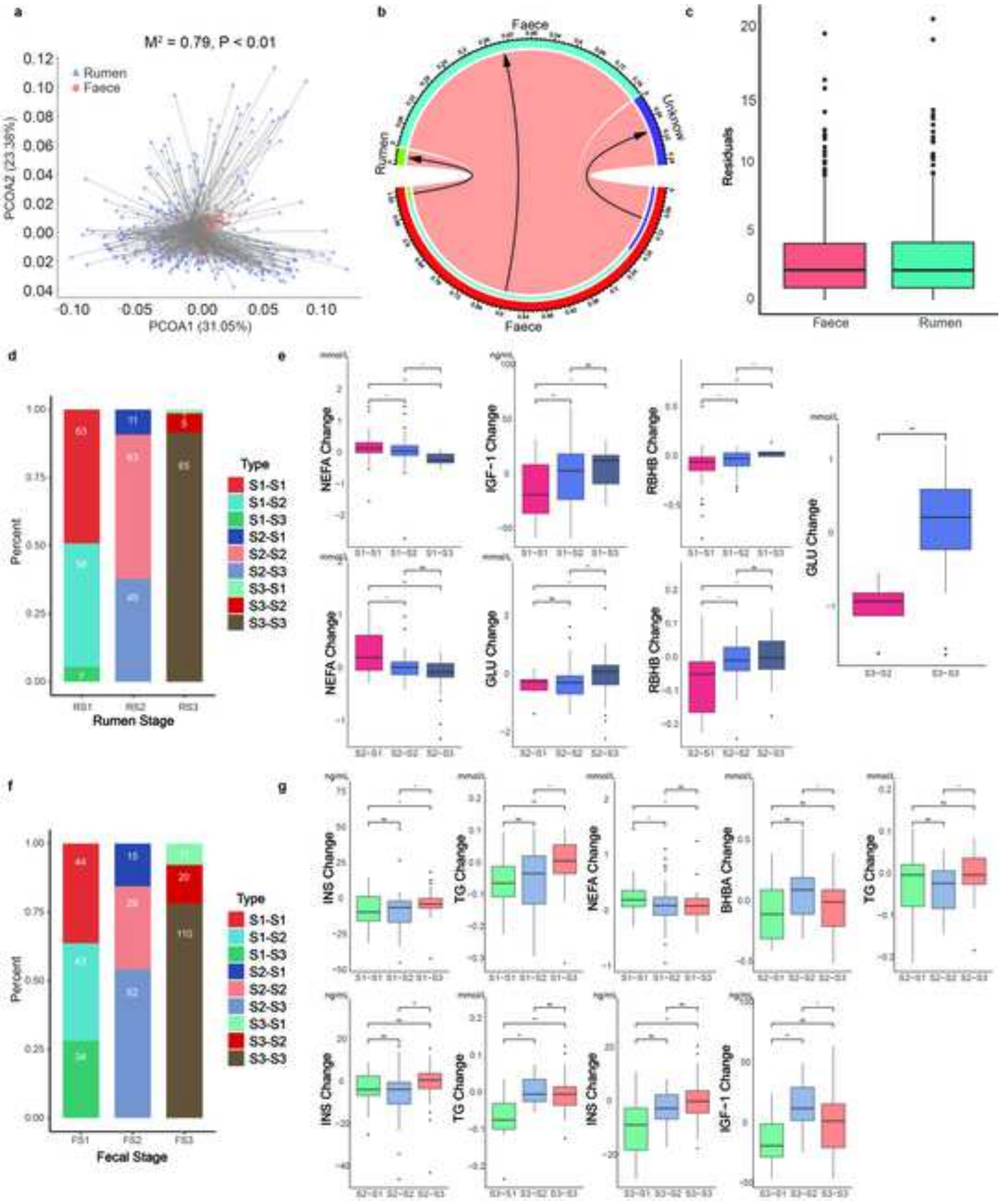

Figure9 Click here to access/download;Figure;Figure 9.tif

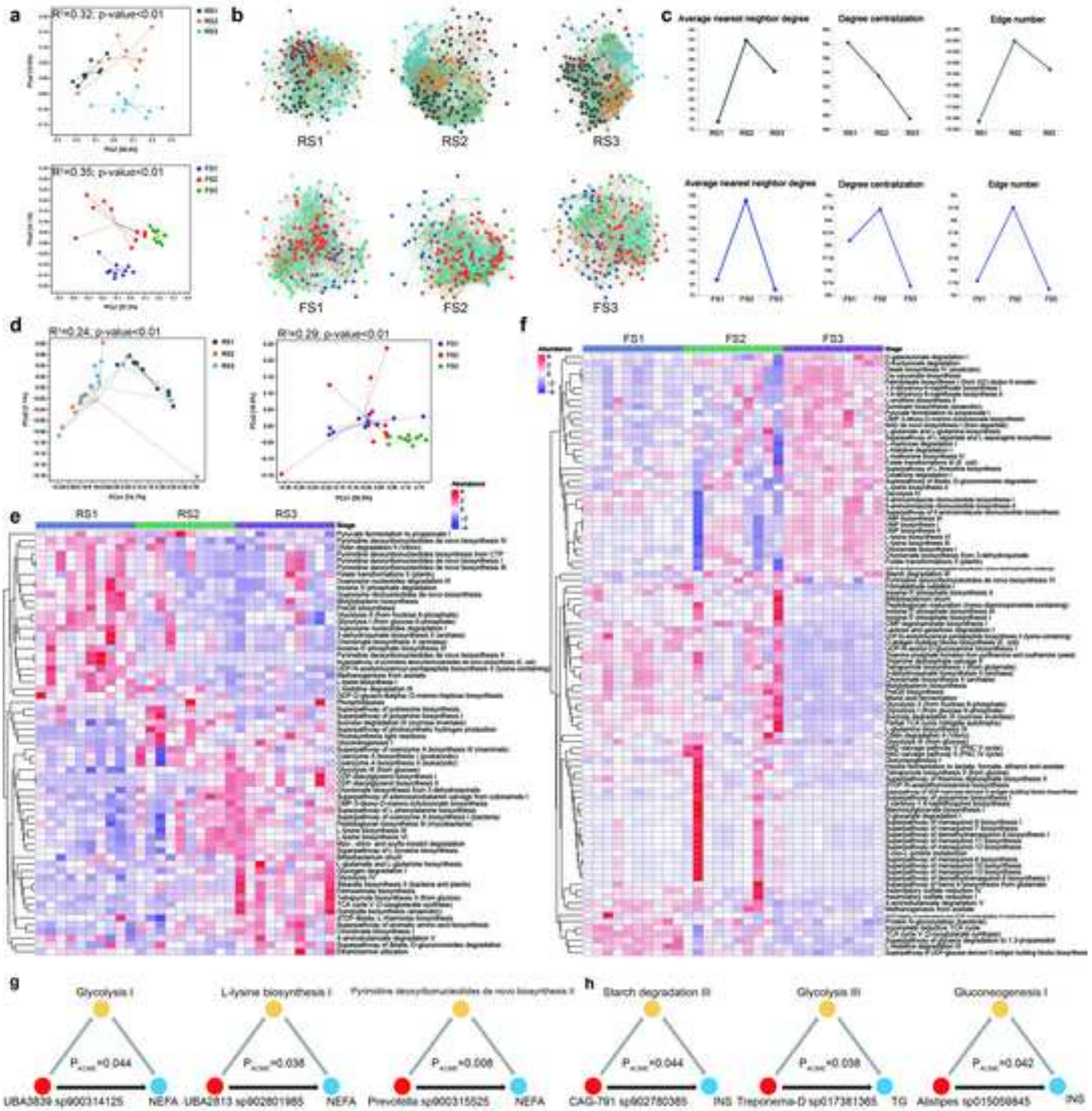

Supplementary Figures

Click here to access/download
Supplementary Material
Supplementary Figures.docx

Supplementary Tables

Click here to access/download
Supplementary Material
Supplementary Tables.xlsx
