# Supplementary material for: Deterministic succession patterns in the rumen and fecal microbiome associate with host metabolic shifts in peripartum dairy cattle
Source: Gigascience. 2025 May 19;14:giaf042. doi: 10.1093/gigascience/giaf042 (PMC12087452; doi:10.1093/gigascience/giaf042)
Supplement: giaf042_GIGA-D-24-00404_Revision_1 [file giaf042_giga-d-24-00404_revision_1.pdf]

GigaScience

Deterministic Succession Patterns in the Rumen and Fecal Microbiome Associate with host Metabolic Shifts in Periparturient Dairy Cattle

--Manuscript Draft--

**Manuscript Number:**	GIGA-D-24-00404R1
**Full Title:**	Deterministic Succession Patterns in the Rumen and Fecal Microbiome Associate with host Metabolic Shifts in Periparturient Dairy Cattle
**Article Type:**	Research
**Funding Information:**	National Natural Science Foundation of China (32130100) — Prof.Dr. Shengli Li
**Abstract:**	Background: Metabolic disorders in periparturient ruminants affect health and productivity, with gut microbiota playing a key role in host metabolism. Therefore, our study aimed to characterize the gut microbiota of periparturient dairy cows to better understand the relationship between metabolic phenotypes and the rumen and fecal microbiomes during the periparturient period.<br>Results: In a longitudinal study of 91 periparturient cows, we analyzed rumen and fecal microbiomes via 16S rRNA and metagenomic sequencing (NovaSeq™X Plus platform) across six time points. By employing enterotype classification, ecological model, and random forest analysis, we identified distinct deterministic succession patterns in the rumen and fecal (rumen: rapid transition-transition-stable; hindgut: stable-transition-stable). Key microbes, such as Succiniclasticum and Bifidobacterium, were found to drive microbial succession by balancing stochastic and deterministic processes. Notably, we observed that changes in gut microbiota succession patterns significantly influenced metabolic phenotypes (e.g., serum non-esterified fatty acid, glucose, and insulin level). Mediation analysis suggested that specific gut microbes (e.g., Prevotella sp900315525 in the rumen and Alistipes sp015059845 in the hindgut) and metabolic pathways (e.g., glucose-related pathway) were associated with host metabolic phenotypes.<br>Conclusions: Overall, utilizing a large gut microbiome dataset and enterotype- and ecological model-based microbiome analyses, we comprehensively elucidated the succession and assembly of the gut microbiota in periparturient dairy cows. We further confirmed that changes in gut microbiota succession patterns were significantly related to the metabolic phenotypes of periparturient dairy cows. These findings provide valuable insights for developing health management strategies for periparturient ruminants.<br>Keywords: microbiome, dynamics, longitudinal study, cow, metabolic phenotypes, periparturient period
**Corresponding Author:**	Shengli Li<br>China Agricultural University<br>Beijing, --- Select One --- CHINA
**Corresponding Author Secondary Information:**	
**Corresponding Author's Institution:**	China Agricultural University
**Corresponding Author's Secondary Institution:**	
**First Author:**	Shuo Wang
**First Author Secondary Information:**	
**Order of Authors:**	Shuo Wang
	Fanlin Kong
	Dongwen Dai
	Chen Li

Powered by Editorial Manager® and ProduXion Manager® from Aries Systems Corporation

	Yangyi Hao
	Erdan Wang
	Zhijun Cao
	Yajing Wang
	Wei Wang
	Shengli Li
**Order of Authors Secondary Information:**	
**Response to Reviewers:**	Response to the editor and reviewers<br><br>February 27th, 2025<br><br>Manuscript ID: GIGA-D-24-00404<br>Title: Deterministic Succession Patterns of Rumen and Hindgut Microbiome Driving Host Metabolism in Periparturient Ruminants: Insights from a Large-Scale Longitudinal Study of Cows<br><br>Dear editor and reviewers:<br><br>Thank you for your constructive comments. We have made the following corrections in accordance with your valuable suggestions.<br><br>Comments from Reviewers:<br>Reviewer #1: Thanks to all author for the excellent study<br>Response: Thank you very much for your positive evaluation of our work. We will address your comments one by one.<br><br>Comment 1: It was good to see if the authors collected sample 7 day of pre-partum because 21 days of prepartum is a long time which is hard to estimate the changes over 21-day prepartum to 0 day.<br>Response: Thank you for your professional insights. During the study design phase, we did consider including the 7-day prepartum period (-7 d) as a key sampling point. However, practical implementation revealed significant limitations in this sampling window due to biological variations in individual calving times (with some cows even entering the periparturient period up to 15 days earlier than anticipated):<br>(1) Although population averages exist, the intense metabolic fluctuations occurring during this narrow -7 d window (proximal to calving) could compromise data accuracy if these samples were included.<br>(2) Prepartum cows represent a vulnerable population, and sampling at -7 d might impose unnecessary stress on both dams and offspring.<br>(3) The postpartum period constitutes the high-risk phase for bovine diseases, thus omitting the -7d sampling point does not diminish the clinical relevance of this investigation.<br>This protocol modification aligns with the 3R principles (Replacement, Reduction, Refinement) of animal research ethics while enabling precise monitoring of dynamic metabolic adaptations during the critical postpartum period, thereby ensuring high-value research outcomes.<br><br>Comment 2: It is not clearly mentioned in the methodology which sequencing data used for which analysis.<br>Response: As suggested, we added the relevant content in the Bioinformatics and statistical analysis section has been described in detail. (Lines 564-592 and Lines 602-614)<br><br>Comment 3: Did author observe any correlation between provided food and changes in rumen and hindgut microbiome<br>Response: In fact, we used the EnvFit and MassLine2 methods to analyze the correlations between diet type, components, and the microbiome. Details can be found in the section "Contribution of multiple individual factors to gut microbial succession " and Fig.7.

Powered by Editorial Manager® and ProduXion Manager® from Aries Systems Corporation

Comment 4: Title “Deterministic Succession Patterns of Rumen and Hindgut Microbiome Driving Host Metabolism in Periparturient Ruminants” is not clearly presenting the study. I would suggest modifying.
Response: As suggested, we modified to ‘Deterministic Succession Patterns in the Rumen and Fecal Microbiome Associate with host Metabolic Shifts in Periparturient Dairy Cattle’. The title not only highlights the main findings but also aligns with Reviewer 2's emphasis on the correlation and the need to avoid redundancy in the title.

Comment 5: Line 23: which platform of metagenome sequencing
Response: As suggested, we added the information of platform. (Line 22)

Comment 6: Line 36: Significantly (which test)
Response: Our differential analysis was performed using the Kruskal-Wallis (KW) test, as mentioned in the section on statistical methods. (Line 625)

Comment 7: Line 51: please provide exact definition of periparturient period with reference
Response: As suggested, we added the reference. (Line 49)
Grummer R. R. (1995). Impact of changes in organic nutrient metabolism on feeding the transition dairy cow. Journal of animal science, 73(9), 2820–2833. https://doi.org/10.2527/1995.7392820x

Comment 8: Line 63: Sentence is not complete
Response: As suggested, we revised the relative sentence. (Lines 62-66)

Comment 9: Line 64: over “the” time
Response: The relevant statement has been removed in the latest revision.

Comment 10: Line 69: to understand
Response: As suggested, we modified the problem. (Line 68)

Comment 11: Line 71-75: Provide more example of cattle and cow. Study on pregnant women is not relating the earlier sentence.
Response: As suggested, we have removed the references about pregnant women and added references related to dairy cows. To avoid redundancy in citations, we have included only the representative articles published in Nature Communications. (Line 73-75)

Comment 12: Line: 97-107: these are the part of methodology. I will suggest avoiding in introduction section.
Response: As suggested, we rewrote the relevant content. (Lines 99-102)

Comment 13: Line: 115: Which timepoint or days
Response: As suggested, we revised the relevant content. (Lines 109-112)

Comment 14: Line: 117: Which type of drastic change, decrease or increase
Response: In accordance with the suggestions from Reviewer 2, we have revised it to "significant change." The primary purpose of this revision is to highlight the emergence of significant changes, as the significant changes in the rumen and feces are opposite to each other. This can be observed in the supplementary Figures. (Line 112)

Comment 15: Line: 124: which results?
Response: In accordance with the suggestions from Reviewer 2, we have incorporated this result into Fig. S1A and Fig. S2A.

Comment 16: Line133: Please describe clearly the pattern of genera depending on the pre and postpartum stages. RDMM1 prominently appeared until 7 d postpartum and what in 14 and 21 days of postpartum? Similarly, RDMM2 and RDMM7 were increasing with the timepoints of post-partum.
Response: As suggested, we revised the relevant content. (Lines 128-142)

Comment 17: Line 140: use past tense and over “the” time and full stop at the end.

Powered by Editorial Manager® and ProduXion Manager® from Aries Systems Corporation

	Response: As suggested, we revised the relevant problem. (Line 143)<br><br>Comment 18: Line 141-142: Remove third bracket<br>Response: As suggested, we revised the relevant problem. (Line 141)<br><br>Comment 19: Line 141: RDMM7 started to appear on 3 d postpartum and gradually increase over the timepoints.<br>Response: As suggested, we revised the relevant problem. (Line 141-142)<br><br>Comment 20: Line 143-145: It is not clear to me. What does timeline of dominance shift mean?<br>Response: As suggested, we revised the relevant problem. (Line 143)<br><br>Comment 21: Line 146: What type of instability observed?<br>Response: As suggested, we revised the relevant problem. (Line 146)<br><br>Comment 22: Line 147: periparturient age points?<br>Response: As suggested, we revised the relevant problem. (Line 147)<br><br>Comment 23: Line 151: "Similarly, FDMMs exhibited distinct occurrence windows and dominant genera (Figs. 2h and i)" sentence is not complete, occurrence based on?<br>Response: As suggested, we revised the relevant problem. (Lines 150-151)<br><br>Comment 24: Line 154: disappeared "at" postpartum<br>Response: As suggested, we revised the relevant problem. (Line 154)<br><br>Comment 25: Line 155: after postpartum or with postpartum.<br>Response: As suggested, we added the 'with' (Line 155)<br><br>Comment 26: Line 155: "with FDMM2 being characterized by Rikenellaceae_RC9_gut_group, Muribaculaceae, and Alistipes, and FDMM3 by Bacteroidales_RF16_group and Prevotellaceae_UCG_003" use separate sentence<br>Response: As suggested, we separated the sentence. (Lines 155-157)<br><br>Comment 27: Line 159: with a peak at 3 d postpartum<br>Response: As suggested, we revised the relevant problem. (Lines 159)<br><br>Comment 28: Line 151-160: clearly mention which figures are related. Are those lines not related with figure 2h if yes then which figure is related with community structure. Please make sure figures are mentioned clearly after each description or before description<br>Response: To avoid redundancy in labeling, we have added the annotation on Line 151.<br><br>Comment 29: Line 163-164: RDMM and FDMM are not dominated with similar type of genus in each cluster, so comparison between them has no meaning<br>Response: Thank you for the suggestion. We have removed the phrase "Unlike in the rumen." (Line 162)<br><br>Comment 30: Line 167: Did you include all 91 animals?<br>Response: Yes, we have added the '91'. (Line 168)<br><br>Comment 31: Line 175-176: Please mention the percentage of transformation over the time.<br>Response: As suggested, we added the relative content. (Lines 173-177)<br><br>Comment 32: Line 180: How is it suggesting a return to stability?<br>Response: We have removed this inferential statement from the results section to ensure an objective description of the findings.<br><br>Comment 33: Line 182: What does "microbial community types" mean?<br>Response: We have revised "microbial community types" to "microbial clusters." (Line 182)

Comment 34: Line 185: What are self-transition rates?
Response: The self-transition rates refer to the probability that the DMM remains unchanged at the next time point. For example, the probability that DMM1 on day 1 postpartum is still DMM1 on day 3 postpartum.

Comment 35: Line 195-196: “previously mentioned genera, including Rikenellaceae_RC9_gut_group, Prevotella, Acetitomaculum, and F082” this line is not clear.
Response: As suggested, we revised the relevant problem. (Lines 195-197)

Comment 36: Line 201-203: this could be the part of discussion.
Response: Thank you for the suggestion. We have removed this part from the results section.

Comment 37: Line 208: In RDMM analysis, author used top100 and in FDMM top 120 ASVs-is there any reason of this change?
Response: This is the result after model optimization based on five 10-fold cross-validations, and the relevant details have been described in the Materials and Methods section. （Lines 576-578）

Comment 38: Line 220: both rumen and faeces.
Response: As suggested, deleted ‘the’ (Line 219)

Comment 39: Line 222-223: Sentence is not clear.
Response: We have split this sentence into two to improve readability. (Line 218-222)

Comment 40: Line 268: What does last time point, please specify.
Response: We revised it to "the previous sampling timepoint" to enhance readability. (Line 267)

Comment 41: Line 273: What does “residuals” mean, please define.
Response: Thank you for your question. The “residuals” refer to the differences between the observed values of the dependent variable the values predicted by our statistical model. These residuals represent the portion of the data that is not explained by the model.

Comment 42: Line 320: “Systematically investigate” is that correct terminology to describe the trial?
Response: Thank you for the suggestion. We have removed the word "Systematically" to increase the accuracy of the statement.

Comment 43: Line 336-338: Avoid repetition of the methodology.
Response: Thank you very much for the suggestion. In the latest version, we have removed this sentence.

Comment 44: Line 379: Affect
Response: Thank you very much for the suggestion. In the latest version, we have removed this sentence.

Comment 45: Line 381: Stage means post-partum stage?
Response: One of the main findings of this study is the identification of three deterministic successional stages in both the rumen (rapid transition, transition, stabilization) and feces (stabilization, transition, stabilization) of periparturient dairy cows. In the rumen, Stage 1 transitions directly to Stage 3 (stabilization) without passing through the intermediate transition stage. For more details, please refer to Fig. 3, Fig. 4, and Fig. 8.

Comment 46: 427: typo, bran or barn?
Response: Thank you very much, the problem has been made. (Line 462)

Comment 47: 443: Specification of the tube.
Response: As suggested, we added the information. (Lines 185-187)

Comment 48: 449: Did author used similar method and kit of DNA extraction from

rumen and faeces samples.
Response: Yes, it is the same. We have added "from all samples." (Line 510)

Comment 49: 511: RDMM and FDMM clusters were defined based on 16S sequencing or metagenomics data? And what is the lowest Laplace approximation score?
Response: In the DMM model analysis using 16S data, the Laplace approximation score is used to evaluate the goodness of fit for models with different numbers of components. It is a key indicator for determining the optimal number of microbial communities. Consistent with common information criteria (such as AIC/BIC), a lower Laplace score indicates a better model fit.

Comment 50: 536: What does R threshold mean?
Response: As suggested, the wording has been revised. (Lines 606-607)

Comment 51: 539-540: the sentence is not clear
Response: As suggested, the wording has been revised. (Lines 610-612)

Comment 52: 550: specification of each kit
Response: As suggested, we added the information. (Lines 499-502)

Comment 53: 551: Briefly describe the procedure of ELISA briefly.
Response: As suggested, we added the information. (Lines 502-505)

Comment 54: 571: Which individual factors?
Response: As suggested, we added the information. (Lines 519-512)

Comment 55: 572: How the false discovery rate adjusted?
Response: Thank you for your question regarding the adjustment of the false discovery rate (FDR). In our study, we used the Benjamini-Hochberg method to adjust the FDR. (Line 594)

Comment 56: Figure 7: In x axis did not use the same value which affecting the Stack bars. I will suggest y to make similar values in x-axis.
Response: As suggested, we have revised Figure 7.

Comment 57: Figure 3b and 4b: What does three stage means and how they defined “stabilization, transition and stabilization”?
Response: We defined it based on the results of the Markov chain analysis. For details, see the Results section (Lines 182-197).

Reviewer #2: This study provides a comprehensive longitudinal analysis of rumen and hindgut microbiome dynamics in periparturient dairy cows, linking microbial succession patterns to host metabolic phenotypes. The work is well-structured, methodologically rigorous, and addresses a critical gap in understanding how gut microbiota transitions during the periparturient period influence metabolic health. The integration of high-resolution temporal sampling, ecological modeling, and mediation analysis strengthens the novelty and impact of the findings. However, some aspects of experimental design, statistical reporting, and interpretation require clarification or expansion before publication.
Response: Thank you very much for your positive evaluation of our work. We will address your comments one by one.

Comment 1: Why were rumen and fecal samples studied separately when analyzing the dynamic succession of the gut microbiota and its relationship with host metabolism in periparturient cows? Not as a whole. Please clarify.
Response: In the study of ruminants, rumen and fecal samples were analyzed separately, primarily because they represent distinct microbial niches and functions. The rumen, as the core site of foregut fermentation, hosts microbial communities (such as fiber-degrading bacteria and volatile fatty acid–producing microbes) that directly dominate energy supply, which is especially crucial during the periparturient period when energy demands surge. In contrast, fecal microbiota reflect the residual microbial communities after the entire digestive process, including digestion, absorption, and metabolism, and are more associated with hindgut immune regulation and metabolic

waste processing. Additionally, rumen microbiota responds more rapidly to changes in diet and hormones, while fecal microbiota, due to the longer gut transit time, may exhibit lagged dynamics. Separate analyses can precisely distinguish their independent contributions to host metabolism (such as energy balance and inflammation regulation), avoid the mixing of functional signals, and provide a scientific basis for differentiated intervention strategies targeting the rumen or hindgut (such as probiotic addition and dietary adjustment).

Comment 2: The causal inference that "microbial communities drive host metabolic changes" requires cautious phrasing. Although mediation analysis was used to infer microbial-driven metabolic alterations, the observational nature of the study inherently limits the ability to exclude confounding factors, rendering causal claims speculative. The authors must revise such statements to avoid overstating conclusions.
Response: Thank you for the reviewer's suggestions. We have avoided such expressions in the title, abstract, and discussion sections to enhance the rigor of the study. (Title, line 30 and34, Discussion)

Comment 3: For host indicators, please explain why only energy metabolism, liver function, and antioxidant indicators were analyzed.
Response: In the study of periparturient dairy cows, energy metabolism, liver function, and antioxidant indicators are typically analyzed with priority, as they are directly related to the core physiological challenges of this period. Energy metabolism indicators (such as NEFA and BHBA) reflect the negative energy balance caused by the onset of lactation and reduced feed intake, which can warn of metabolic risks such as ketosis. Liver function indicators (AST and ALT) assess the liver's overload in carbohydrate and lipid metabolism and detoxification, revealing potential damage such as fatty liver. Antioxidant and stress indicators (T-AOC and HP) monitor the level of oxidative stress, and their imbalance can exacerbate tissue damage and immune suppression. These three categories of indicators have recently been considered interrelated and together form the key targets for monitoring periparturient metabolic health, providing a basis for early intervention.

Comment 4: Key methodological details are missing, hindering reproducibility. For example, descriptions of ICAMP and mediation analysis are insufficient
Response: In accordance with your suggestions and those of Reviewer 1, we have revised the Materials and Methods section to enhance the reproducibility and readability of this study. (Lines 559-614)

Comment 5: The Discussion lacks depth. Expand the interpretation of microbial succession patterns and host-microbe interactions observed in the results.
Response: As suggested, the discussion section has been rewritten to provide in-depth explanations of the key findings. (Discussion section)

Comment 6: Title: Overly verbose. Shorten to emphasize the core focus and novelty. Replace "Periparturient Ruminants" with "Periparturient Dairy Cows" for precision
Response: As suggested, we revised the title

Comment 7: Line 24: Revise "ecological modeling" to "ecological model" for grammatical consistency.
Response: As suggested, we have revised the problem. (Line 23)

Comment 8: Abbreviations: Define "RQUICKI-BHB" in full upon first mention: Revised Quantitative Insulin Sensitivity Check Index-BHB. Provide the full name for "MaAsLin2": Multivariate Analysis by Linear Models. Ensure all abbreviations (e.g., TMR, DMM, ICAMP) are defined at their first occurrence.
Response: Thank you for the suggestion. We have revised the entire manuscript to address the problems.

Comment 9: Line 117: Replace the colloquial phrase "drastic changes" with "significant changes" for scientific rigor.
Response: As suggested, we have revised the problem. (Line 112)

Comment 10: Line 123: Explicitly annotate the PCoA plot to display the variance percentages (26.14% and 24.96%).

Response: As suggested, we have incorporated this result into Fig. S1A and Fig. S2A.

Comment 11: Line 124: The sentence "These results highlight their individualized states..." is interpretive and belongs in the Discussion, not Results.
Response: The relevant statement has been removed from the results section.

Comment 12: Line 126 Justify the use of the Dirichlet Multinomial Mixture (DMM) model instead of the more common Partitioning Around Medoids (PAM) method for clustering.
Response: In microbiome data analysis, the Dirichlet Multinomial Mixture (DMM) model has significant advantages over Partitioning Around Medoids (PAM), especially when dealing with high-dimensional, sparse discrete data (such as OTU/ASV count data). The DMM, based on the Dirichlet Multinomial distribution, can directly model the compositional nature of microbiome data, capturing its heterogeneity and complex community structure. In contrast, PAM, as a distance-based clustering method, typically assumes continuous data and struggles to handle the characteristics of discrete data directly. Moreover, DMM does not require pre-specifying the number of clusters and can automatically determine the optimal number through model selection methods (such as BIC or AIC), making it suitable for exploratory analyses. In contrast, PAM requires manually specifying the number of clusters, which may introduce subjective bias. DMM is also less sensitive to outliers and provides probabilistic interpretability that can be directly linked to the biological significance of microbiome data, making it particularly suitable for analyzing dynamic changes in time-series data. In comparison, PAM lacks probabilistic interpretability and has weaker capabilities for capturing dynamic changes. Therefore, in the dynamic analysis of the periparturient dairy cow microbiome, the DMM model is a more appropriate choice, especially when capturing complex community structures and dynamic changes is required.

Comment 13: Terminology clarity: Replace "DMM" with "cluster" throughout the text to improve readability, unless the method itself is a key focus.
Response: Thank you very much for your suggestions. During the manuscript writing process, we also paid attention to the consistency of terminology and readability. As you pointed out, DMM not only represents the results of clustering but also reflects the uniqueness of the methodology. To ensure that readers can understand clearly, we provided a detailed definition of DMM when it was first mentioned in the results section, and subsequently used “DMM” or “cluster” appropriately according to the context to balance professionalism and readability.

Comment 14: Line 420-428 Periparturient cows usually receive postpartum care. What is the care plan in this experiment?
Response: Thank you for the reminder We have added the relevant content. (Lines 163-167)

Comment 15: Line 434 Clarify whether all 91 cows completed sampling at all six time points. Provide details on sample loss rates or exclusion criteria
Response: Thank you very much for your suggestion. In this experiment, to ensure the accuracy of the results, we ensured that each cow had at least four samples for rumen or feces. The detailed information on sample missingness has been included in the supplementary tables, where blanks indicate missing samples for a particular cow. (Table S17 and S18)

Comment 16: Line 435, If cows were sampled on precise days, it would be essential to provide detailed information regarding the sampling timeline. For example, if Day -21 was the target sampling day, the actual average, standard deviation, and range of days relative to this target should be specified. Similarly, for samples collected on Day 1, it would be important to report the range, average, and standard deviation of the number of hours elapsed since calving. I suggest including these details in the Methods and Materials section where they would fit best.
Response: Thank you for your suggestion. In fact, the detailed sampling times for Day -21 are already shown in Columns P-A of the supplementary dataset "CowInformation.csv." Consistently, Day 1 samples were collected on the second day after calving, regardless of the exact time of parturition. We have added the necessary details to the Materials and Methods section. (Lines 477-480)

Powered by Editorial Manager® and ProduXion Manager® from Aries Systems Corporation

	Comment 17: Line 445: Standardize notation to "3,000 × g" (comma separator for thousands).<br>Response: As suggested, we have revised the problem. (Line 489)<br><br>Comment 18: Lines 504-505: Use Greek symbols: α-diversity and β-diversity (revise throughout the manuscript).<br>Response: Thank you for the suggestion. We have revised the entire manuscript to address the problems.<br><br>Comment 19: Line 527: Cite references for the residual analysis methodology.<br>Response: As suggested, we added the reference. (Line 601)<br><br>Comment 20: Lines 122 - 165: Use either "fecal microbiota" or "hindgut microbiota" consistently for intestinal - related descriptions.<br>Response: As suggested, we have replaced "hindgut microbiota" with "fecal microbiota" throughout the manuscript.<br>Comment 21: Lines 831 - 834: Increase the font size in Sanky and Markov chain diagrams of Figures 3 and 4 for better readability.<br>Response: As suggested, we have revised the relevant content to enhance readability.
Additional Information:	
Question	Response
Are you submitting this manuscript to a special series or article collection?	No
**Experimental design and statistics**<br><br>Full details of the experimental design and statistical methods used should be given in the Methods section, as detailed in our Minimum Standards Reporting Checklist. Information essential to interpreting the data presented should be made available in the figure legends.<br><br>Have you included all the information requested in your manuscript?	Yes
**Resources**<br><br>A description of all resources used, including antibodies, cell lines, animals and software tools, with enough information to allow them to be uniquely identified, should be included in the Methods section. Authors are strongly encouraged to cite Research Resource Identifiers (RRIDs) for antibodies, model organisms and tools, where possible.<br><br>Have you included the information requested as detailed in our Minimum	Yes

Powered by Editorial Manager® and ProduXion Manager® from Aries Systems Corporation

Standards Reporting Checklist?	
**Availability of data and materials**<br><br>All datasets and code on which the conclusions of the paper rely must be either included in your submission or deposited in publicly available repositories (where available and ethically appropriate), referencing such data using a unique identifier in the references and in the "Availability of Data and Materials" section of your manuscript.<br><br>Have you have met the above requirement as detailed in our Minimum Standards Reporting Checklist?	Yes

Manuscript with DOI

Click here to access/download;Manuscript;Manuscript R1 NN edits.docx

Deterministic Succession Patterns in the Rumen and Fecal Microbiome Associate with host Metabolic Shifts in Periparturient Dairy Cattle

Shuo Wang†, Fanlin Kong†, Dongwen Dai, Chen Li, Yangyi Hao, Erdan Wang, Zhijun Cao, Yajing Wang, Wei Wang*, and Shengli Li*

State Key Laboratory of Animal Nutrition and Feeding, Department of Animal Nutrition and Feed Science, College of Animal Science and Technology, China Agricultural University, Beijing 100193, China

***Correspondence:**

Shengli Li: lishengli@cau.edu.cn; Tel and fax: +86-010-62731254;

Wei Wang: wei.wang@cau.edu.cn; Tel and fax: +86-010-62733789

†These authors contributed equally: Shuo Wang and Fanlin Kong

Abstract

Background: Metabolic disorders in periparturient ruminants affect health and productivity, with gut microbiota playing a key role in host metabolism. Therefore, our study aimed to characterize the gut microbiota of periparturient dairy cows to better understand the relationship between metabolic phenotypes and the rumen and fecal microbiomes during the periparturient period.

Results: In a longitudinal study of 91 periparturient cows, we analyzed rumen and fecal microbiomes via 16S rRNA and metagenomic sequencing (NovaSeq™X Plus platform) across six time points. By employing enterotype classification, ecological model, and random forest analysis, we identified distinct deterministic succession patterns in the rumen and fecal (rumen: rapid transition-transition-stable; hindgut: stable-transition-stable). Key microbes, such as *Succiniclasticum* and *Bifidobacterium*, were found to drive microbial succession by balancing stochastic and deterministic processes. Notably, we observed that changes in gut microbiota succession patterns significantly influenced metabolic phenotypes (e.g., serum non-esterified fatty acid, glucose, and insulin level). Mediation analysis suggested that specific gut microbes (e.g., *Prevotella sp900315525* in the rumen and *Alistipes sp015059845* in the hindgut) and metabolic pathways (e.g., glucose-related pathway) were associated with host metabolic phenotypes.

Conclusions: Overall, utilizing a large gut microbiome dataset and enterotype- and ecological model-based microbiome analyses, we comprehensively elucidated the succession and assembly of the gut microbiota in periparturient dairy cows. We further confirmed that changes in gut microbiota succession patterns were significantly related to the metabolic phenotypes of periparturient dairy cows. These findings provide valuable insights for developing health management strategies for periparturient ruminants.

Keywords: microbiome, dynamics, longitudinal study, cow, metabolic phenotypes, periparturient period

Data Description

We conducted a 42-day dynamic follow-up study on 91 healthy periparturient dairy cows, tracking changes in the rumen microbiome (476 16S rRNA sequencing samples and 30 metagenomic sequencing samples), fecal microbiome (506 16S rRNA sequencing samples and 30 metagenomic sequencing samples), and metabolic phenotypes (505 samples). We established relationships between changes in the gut microbiome and metabolic phenotypes. The metagenomic and 16S rRNA sequencing data used in this study have been archived in the NCBI database under accession numbers PRJNA1161368 and PRJNA1126601, respectively.

Introduction

Ruminants play a crucial role in global food supply and sustainable agriculture [1]. The periparturient period, defined as 3 wk before until 3 wk after calving, is one of the most vulnerable times in a ruminant life [2]. During this phase, due to calving, dietary changes, and onset of lactation, ruminants undergo substantial physiological and metabolic adjustments [3-5]. Approximately 30–50% of dairy cows experience postpartum metabolic diseases, including ketosis, hypocalcemia, and retained placenta [5, 6]. These conditions affect the health and productivity of ruminants and lead to substantial economic losses [7].

Ruminants have evolved a unique rumen structure that enables multiple host-microbiome interactions: host-rumen microbiome and host-fecal microbiome interactions. The rumen microbiome is essential for cellulose breakdown, short-chain fatty acid production, nitrogen cycling, and vitamin synthesis [8]. The fecal microbiome contributes to energy and nutrient absorption and modulates the host immune system [9]. Additionally, extensive research in adult dairy cows showed correlations between the rumen and fecal microbiomes with milk quality and feed efficiency [10, 11], further highlighting their critical role in ruminant production and health.

Recent advances in microbial ecology have brought heightened concerns to the temporal succession and ecological assembly of gut microbiomes. Specifically, microbial succession delineates systematic compositional shifts across developmental stages, while microbiome assembly encompasses deterministic processes that establish functionally coherent communities through multilevel interactions spanning host

physiology, microbial cross-feeding, and environmental modulation [12, 13]. This process includes colonization of various microbial species and their interactions with each other and the host environment [13]. To understand microbial succession and assembly is crucial for comprehending how microbial communities are established stably and functionally. This helps understand how these microbial communities impact health. Teddy and Xiao *et al*. identified that the infant gut microbiome can be roughly divided into three succession stages: development, transition, and stable stages [14, 15]. Additionally, they used neutral ecological models to elucidate transformation patterns and driving forces of the infant gut microbiome [15]. Furman *et al.* also found that under the deterministic conditions of diet and age in dairy cows, stochastic effects drive the succession and assembly of rumen microbiome throughout the cow life [16]. Similarly, studies on chicks [17], piglets [18], lambs [19], and calves [20, 21] have highlighted how gut microbiome assembly affects the growth and development of young animals. These studies used large-scale longitudinal methods to explore the succession and assembly patterns of the gut microbiome and their dynamic interactions with the host, providing targeted evidence for promoting healthy development and pregnancy via gut microbiome regulation.

Recently, some studies have attempted to reveal the succession patterns of the rumen and fecal microbiome in periparturient ruminants (primarily focusing on cows) [22-26]. However, due to limitations in sample size and temporal resolution, different studies have found varying patterns of change in the gut microbiome of dairy cows. Zhu *et al*. observed a decrease in the richness of rumen microbiota from the prenatal to the postnatal stages [23]. In contrast, Bach *et al.* found an increase in the richness of rumen microbiota [24]. Moreover, although Zhu *et al.* noted that prepartum fecal microbiota exhibits higher diversity, primarily composed of Firmicutes and Bacteroidetes [25], Luo *et al.* reported no marked differences in diversity before and after calving, with slight differences in phylum composition [26]. Although these studies indicate varying results, they consistently suggest the potential for remodeling the gut microbiome in periparturient cows. Considering the substantial metabolic changes during the periparturient period, there remains a gap in our understanding of the succession patterns of rumen and fecal microbiomes and their impact on host metabolism. Additionally, exploring key factors driving these microbiome dynamics is crucial. Understanding these factors could potentially allow us to predict and determine when and how to intervene in the microbiome assembly process to modulate its structure and

function. Besides normal dynamic changes, individual factors (e.g., parity and body condition) have previously been reported to correlate with ruminant gut microbiomes [27, 28]. A comprehensive analysis of these factors will aid in better understanding the dynamic changes of the microbiome in periparturient ruminants.

In this study, we used dairy cows with highly controlled feeding systems, diets, and housing as our subjects. By conducting a longitudinal observation of the gut microbiome and host metabolic indicators of dairy cows 21 days before until 21 days after calving, we aim to enhance our detailed understanding of the gut microbiome and host metabolic characteristics in periparturient ruminants, potentially facilitating the development of new strategies to improve postpartum health in ruminants.

Results

Dynamic changes in gut microbial composition in periparturient dairy cows

Through a rigorous prospective cohort study design (Fig. 1), we observed a clear separation of gut microbiota in periparturient dairy cows among the sampling time points, underscoring the remodeling of the cow gut microbiota during periparturient periods (Figs. S1a and S2a). The α-diversity (Chao1 and Shannon indexes) of ruminal microbiota increased 21 days before until 1 days after calving, decreased from 1 to 7 days after calving, and stabilized from 7 to 21 days after calving (Fig. S1b). Conversely, the α-diversity of fecal microbiota decreased 21 days before until 3 days after calving, increased from 3 to 7 days after calving, and stabilized from 7 to 21 days after calving (Fig. S2b). We also observed significant changes in the α-diversity of rumen microbiota at just 1 day postpartum. Conversely, fecal microbiota showed a similar response but at 3 days postpartum. Additionally, in both rumen and fecal samples, the dominant microbial phyla were Firmicutes, Bacteroidetes, Actinobacteria, Spirochaetes, and Proteobacteria (Figs. S1c and S2c).

Importantly, we observed significantly greater inter-individual than intra-individual variability across both rumen and fecal microbiota (Figs. 2a and f), and the inter-individual variation in the rumen and fecal microbiota constituted 26.14% and 24.96% of the total compositional variation, respectively (PERMANOVA: Permutational multivariate analysis of variance, $p<0.01$, Figs. S1a and S2a). To more accurately demonstrate microbial succession in the rumen fluid and feces of periparturient cows, we

applied the dirichlet multinomial mixture (DMM) method. At the genus level, based on the lowest Laplace approximation scores (Figs. S1d and S2d), the analysis yielded seven and five DMM clusters for rumen and fecal samples, respectively (Figs. 2b and g). Regarding ruminal microbiota, *Prevotella*, *NK4A214_group*, *Lachnospiraceae_NK3A21_group*, *Acetitomaculum*, and *Succiniclasticum* were the top five genera defining RDMMs (DMM clusters for rumen microbiota; Fig. S1e). The heatmap in Figure S1f displays the distribution of these genera across different RDMMs. Each RDMM exhibited a unique timing of appearance and dominant genera (Fig. 2c and d). RDMM1 was dominated by *Succiniclasticum*, prominently appearing until 7 days postpartum, and its proportion increased from 7 to 21 days postpartum; RDMM2, characterized by *Muribaculum* and *Prevotella*, first appeared on day 1 postpartum, with its proportion increasing from 1 to 7 days postpartum, and then gradually decreasing from 7 to 21 days postpartum; RDMM3, dominated by *Prevotellaceae_UCG_003*, was present before parturition, with its proportion increasing from prepartum to 3 days postpartum, and then gradually decreasing from 3 to 21 days postpartum; On the other hand, RDMM4 and RDMM5 were primarily active on day 21 prepartum and gradually diminished postpartum. Specifically, *FO82* was identified as the dominant genus for RDMM4, while *Lachnospiraceae_NK3A21_group*, *Christensenellaceae_R_7_group*, *Acetitomaculum*, *NK4A214_group*, and *Ruminococcus* were the dominant genera for RDMM5; Finally, RDMM6 emerged on day 1 postpartum with dominant genera including *Rikenellaceae_RC9_gut_group*, *Prevotella_UCG-001*, and *Treponema*, gradually diminishing over the time. By day 3 postpartum, RDMM7, characterized by the *Eubacterium_coprostanoligenes_group*, *Olsenella*, and *Ruminococcus_gauvreauii_group*, began to appear, and gradually increased over the timepoints. In addition to the occurrence windows, we noted the changing community characteristics of the rumen clusters over the time. The dominant clusters shifted from RDMM4 and RDMM5 prepartum to RDMM6 on 1 d postpartum, then to RDMM3 by 3 d, RDMM2 by 7 d, and finally to RDMM1 by 14 and 21 d (Fig. 2c). Further analysis indicated that except for appearing significant decline in RDMM3 at 14 days postpartum, the Shannon diversity of the other RDMM clusters remained relatively stable across the different timepoints (Fig. 2e).

In the fecal microbiota, the top five genera defining FDMMs (DMM clusters for fecal microbiota) were *UCG-005*, *Rikenellaceae_RC9_gut_group*, *Romboutsia*, *Bifidobacterium*, and *UCG-010* (Fig. S2e).

The heatmap in Figure S2f illustrates the distribution of these genera across different FDMMs. Similarly, Each FDMM also exhibited a unique timing of appearance and dominant genera (Figs. 2h and i). FDMM1, characterized by dominant genera, including *Romboutsia*, *Paeniclostridium*, *Lachnospiraceae_NK3A20_group*, and *Christensenellaceae R-7 group*, was primarily observed at 21 d prepartum; the proportion of FDMM1 gradually decreased, and finally, it disappeared at postpartum. FDMM2 and FDMM3 appeared prepartum, and their proportion increased with postpartum. FDMM2 was predominantly defined by *Rikenellaceae_RC9_gut_group*, *Muribaculaceae*, and *Alistipes*. And FDMM3 dominated by *Bacteroidales_RF16_group* and *Prevotellaceae_UCG_003*. FDMM4 did not exhibit any dominant genera, indicating its instability, whereas FDMM5 was dominated by Bacteroides. The two clusters were present throughout the periparturient period, with a peak at 3 d postpartum. Importantly, the community characteristics of fecal clusters varied over the timepoints (Fig. 2h). FDMM1 was dominant on 21 d prepartum and 1 d postpartum, experiencing a shift on 3 d postpartum with no dominant cluster, and stabilized by 7–21 d postpartum, with FDMM2 and 3 becoming dominant. The Shannon of FDMM1, 2, and 3 initially decreased and then stabilized, whereas that of FDMM4 continued to decrease, and that of FDMM5 fluctuated (Fig. 2j).

Dynamics of the individual gut microbiota in periparturient cows

To delve deeper into the transformation process of microbial community clusters in the rumen and feces in 91 periparturient cows, we analyzed their transitions across different sampling days at the individual level. We observed a distinct trend of transitions between the community clusters throughout the study period (Figs. 3a and 4a; Tables S1 and S2). Specifically, from 21 d prepartum to 1 d postpartum, the RDMM4 to 6 and RDMM5 to 6 transitions represented 24.1% and 16.5% of the total transitions in the rumen, respectively, highlighting them as the dominant transformations (Table S1). Similarly, the RDMM6 to 3 transition was the dominant transformation (23.6%) from 1 d to 3 d postpartum; RDMM3 to 2 (19.2%) and RDMM3 to 3 (17.8%) were the dominant transformations from 3 d to 7 d postpartum; RDMM2 to 1 (16.4%) and RDMM2 to 2 (19.2%) were the dominant transformations from 7 d to 14 d postpartum; and RDMM1 to 1 (28.2%) was the dominant transformation from 14 d to 21 d postpartum (Table S1). Regarding fecal microbiota, the FDMM1 to 1 transition (41.7%) was the dominant

transformation from 21 d prepartum to 3 d postpartum. Interestingly, we did not observe any dominant transformations from 3 d to 7 d postpartum, highlighting the instability of the fecal microbial community structure during this period; however, the FDMM2 to 2 and FDMM3 to 3 transitions dominated from 7 d to 21 d postpartum (Table S2).

To quantitatively integrate the transitions of microbial clusters in the rumen and feces during the peripartum period, we developed a Markov chain model (Figs. 3b and 4b). For rumen, we found that RDMM2, 3, 4, 5, and 6 had high frequencies of transitioning to other clusters, with RDMM4, 5, and 6 exhibiting lower self-transition rates (below 20%), likely contributing to their rapid disappearance during the succession process. Conversely, RDMM2 and 3 exhibited self-transition rates of 45% and 35%, respectively. These two RDMMs were more stable and may thus play a role in bridging microbial succession. In addition, RDMM1 and 7, with self-transition rates of 58% and 64%, respectively, and mutual transition rates above 30%, exhibited stability and maturity during the later stages of the peripartum period. Thus, peripartum microbial succession could be divided into three phases: rapid transition (RDMM4, 5, and 6), transition (RDMM3 and 2), and stabilization (RDMM1 and 7). Based on the above microbial succession patterns, we used a random forest algorithm to construct a classification model. Following five rounds of ten-fold cross-validation, we determined that the model constructed using the top 100 amplicon sequence variants (ASVs) with the highest accuracy exhibited the highest prediction rate (AUC>0.95) (Figs. S3a and Fig. 3c). In addition, we found that the genera, including *Rikenellaceae_RC9_gut_group*, *Prevotella*, *Acetitomaculum*, and *F082*, played key roles in constructing the classification model (Fig. S3a).

In the study of fecal microbiota during the peripartum period, we also observed distinct self-transfer rates among FDMMs. Specifically, FDMM 4 and 5 exhibited lower self-transfer rates of 26% and 23%, respectively. In contrast, FDMM 1, 3, and 2 displayed higher rates of 41%, 46%, and 66%, respectively (Fig. 4b). In addition, the conversion rate of FDMM 4 and 5 to FDMM 1 reached 16% (Fig. 4b). The mutual transfer rates between FDMM 2 and 3 exceeded 20%, reflecting their relative stability and maturity during the microbial succession process. Compared with rumen microbiota, fecal microbiota transitioned into a stable phase within 7 d postpartum, illustrating a shorter transition period. Based on these findings, we categorized fecal microbiota succession into three distinct stages: stabilization

(FDMM1), transition (FDMM4 and 5), and stabilization (FDMM2 and 3). Using the top 120 ASVs with a random forest algorithm, we developed a classification model, which after rigorous cross-validation showed high predictive accuracy (AUC > 0.95) (Fig. S3b and Fig. 4c). We observed that key genera, including *Romboutsia*, *UCG-005*, *Paeniclostridium*, and *Bifidobacterium* played pivotal roles in this model, further underscoring their importance in predicting periparturient fecal microbiota succession patterns (Fig. S3b).

Assembly mechanism of rumen and fecal microbes in periparturient dairy cows

We demonstrated that microbial succession in the rumen and feces of periparturient dairy cows progresses through three stages. Understanding the underlying reasons for these transformations is of great interest. Therefore, we explored the internal driving forces of rumen and fecal microbiota using phylogenetic bin-based null model analysis (ICAMP) of ecological models to elucidate potential factors influencing microbial community dynamics. Our analysis revealed that stochastic processes dominated the microbial assembly in both rumen (81%) and fecal (82%) communities of periparturient dairy cows (Fig. S4a and b). Among these processes, dispersal limitation (DL) emerged as the primary driver in stochastic processes, while homogeneous selection (HOS) constituted the most significant deterministic processes (Fig. S4a and b). We further compared the ecological processes of different microbial clusters, focusing primarily on HOS and DL because of the minor relative contributions of other processes (heterogeneous selection, homogenizing dispersal, and drift). We found significant differences in the HOS and DL processes among the different ruminal and fecal succession patterns (Fig. 5a and Fig. 6a), indicating that ecological processes drive the succession of microbial communities in the rumen and feces of periparturient cows. In the rumen, we observed a significant decline in the proportion of DL processes during the succession period, whereas the proportion of HOS processes increased. Conversely, the fecal microbiota exhibited higher DL and lower HOS during succession, highlighting the key role of deterministic and stochastic processes in the succession of ruminal and fecal microbiota during the periparturient period, respectively.

Furthermore, we divided ruminal and fecal ASVs into 144 and 137 Bins, respectively (Tables S3 and S4). In the rumen, deterministic HOS dominated three of the top 20 relative abundances of RBins (Bins

for rumen microbiota), whereas DL dominated the remaining 17 RBins (Fig. 5b). Conversely, in feces, HOS dominated two of the top 20 relative abundances of FBins (Bins for fecal microbiota), whereas DL dominated the remaining 18 FBins (Fig. 6b). We also presented the abundance and ecological process contributions of the top 20 Bins in the rumen and feces at different successional stages (Figs. 5c and Fig. 6c). In the rumen, *Succiniclasticum*, *Prevotellaceae_UCG-001*, *Prevotella*, *Eubacterium_coprostanoligenes_group*, and *Olsenella* were identified as key drivers of microbial succession, with their changes in relative abundance across different stages aligning with their contributions to the HOS process (Figs. 5d and e). Similarly, in feces, *Bifidobacterium*, *Treponema*, *UCG-005*, *Lachnospiraceae_AC2044_group*, *Rikenellaceae_RC9_gut_group*, and *Monoglobus* were identified as key drivers of microbial succession, with their relative abundance changes across stages consistent with their contributions to the DL process (Figs. 6d and e).

Contribution of multiple individual factors to gut microbial succession

To assess the influence of individual factors on gut microbial succession, the samples were stratified into all and three successional stages for covariate analysis (Fig. 7). We found that factors such as diet, parity, age, calving to days (CD), and pH were associated with rumen microbial succession. Specifically, CD and dietary nutrient levels explained most of the variance in rumen stage 1(RS1), CD and birth weight explained most of the variance in rumen stage 3 (RS3), while rumen stage 2 (RS2) was most strongly related to sire. For fecal microbiota, CD, diet, and age were significant factors associated with microbial succession, with CD and diet explaining most of the variance in fecal stage 1 (FS1). Fecal stage 2 (FS2) was associated with factors such as sire, pH, and predelivery – actual data, and sire also explained the largest variance in fecal stage 3 (FS3).

Furthermore, we further examined the effects of individual factors on key taxa in community assembly using the Massline2 method (Table S5). In the rumen, the relative abundance of *Succiniclasticum*, *Prevotella*, *Prevotellaceae_UCG-001,* *Olsenella*, and *Eubacterium_coprostanoligenes_group* was most strongly associated with CD, diet, and pH. In the hindgut, the results show that the relative abundance of *Lachnospiraceae_AC2044_group* was associated with sire and milk yield in last parity, and the relative abundance of *Monoglobus* was related to diet.

However, other key taxa were not associated with these individual factors.

Gut microbial succession types influence host metabolic phenotypes in periparturient dairy cows

First, we assessed the dissimilarity at the ASV level between the rumen and fecal microbiota, yielding an M^2 value of 0.79 (Fig. 8a). Further source tracking analysis showed that approximately 80% of the fecal microbiota originated from fecal microbiota at the previous sampling timepoint, whereas only 4% could be traced back to rumen microbiota at the same sampling timepoint (Fig. 8b). These results suggest a weak link between the rumen and fecal microbiomes, supporting their consideration as distinct units when studying their effects on the metabolism of peripartum dairy cows. Additionally, the rumen and fecal microbiomes contributed to changes in blood metabolic indicators by 20.64% and 19%, respectively (Table S6). Concomitantly, we did not observe any significant difference in the residuals between these microbiome and blood indicators, further supporting their similar contributions to the host metabolism (Fig. 8c).

Based on these findings, we separately counted the transformation types of gut microbiota and analyzed effects of the different types on host metabolic phenotypes at the individual level (Figs. 8d and f). Significant differences in blood metabolic indicators were observed across different transformation types (Figs. 8e and g). Transformation types in the rumen microbiota significantly influenced host change levels of serum non-esterified fatty acids (NEFA), insulin-like growth factor-1 (IGF-1), revised quantitative insulin sensitivity check index-β-Hydroxybutyrate (RBHB), and glucose (GLU) ($p < 0.05$), while transformation types in the fecal microbiota notably affected serum Insulin (INS), triglycerides (TG), NEFA, β-hydroxybutyrate (BHBA), and IGF-1 change levels ($p < 0.05$).

Transition in the metabolic capacity of the gut microbiome and microbe–host interactions in periparturient dairy cows

To validate our bacterial findings and evaluate functional succession transitions in rumen and fecal microbiome, we analyzed microbial and functional changes across succession stages using metagenomic data. Rumen and fecal microbiome clustered distinctly at the species level (Fig. 9a), and network analysis also revealed significant stage-specific differences in microbial interactions (Figs. 9b and c). For example,

in stable stages of feces (FS1 and FS3), species showed simpler interactions and lower centrality (Fig. 9c). Meanwhile, the node analysis results also revealed that key taxa driving microbial succession, such as ruminal *Prevotella* and *Succiniclasticum*, as well as fecal *Bifidobacterium* and *Treponema*, played pivotal roles in the network composition (Tables S7-12).

Similarly, rumen and fecal microbiota clustered separately at the pathway level (Fig. 9d). We found that the RS2 and RS3 stages were more similar, while FS2 and FS1 stages were closer. Despite having different dominant species at various successional stages, these results further suggest that the successional patterns of rumen and fecal microbiota during the periparturient period are fundamentally different. In addition, metabolic pathways also displayed stage-specific patterns (Figs.9e and f). In the rumen, the RS1 stage was characterized by upregulated pathways involved in pyruvate fermentation, glycolysis, nucleotide biosynthesis, and amino acid metabolism. In the RS3 stage, there was an upregulation of pathways related to the Bifidobacterium shunt, glycogen degradation, and aromatic amino acid biosynthesis. Notably, polyamine biosynthesis, gluconeogenesis, and coenzyme A synthesis were consistently upregulated in both RS2 and RS3 stages. In the hindgut, metabolic pathways such as sulfate reduction and sulfur metabolism, methanogenesis V, and the incomplete reductive TCA cycle were upregulated in the FS1 stage. The FS3 stage exhibited upregulation of pathways related to D-galacturonate degradation I, D-fructuronate degradation, glycolysis IV, L-lysine biosynthesis, and UMP biosynthesis. Common pathways such as inosine-5'-phosphate biosynthesis, glycolysis I and II, mixed acid fermentation, and thiamine metabolism were upregulated in both FS1 and FS2 stages.

Further, we used mediation models to explore the relationships between significantly different species, metabolic pathways, and metabolic phenotypes. In the rumen, we identified 280 relationships with mediating effects, 19 of which showed mediation, direct, and total effects (Table S13). For example, rumen *Prevotella sp900315525* and Pyrimidine deoxyribonucleotides de novo biosynthesis II promotes the elevation of serum NEFA levels. In contrast, the elevated abundance of *UBA3839 sp900314125* and glycolysis I as well as *UBA2813 sp902801985* and L-lysine biosynthesis I reduce serum NEFA levels (Fig. 9g). In the hindgut, we identified 1,360 relationships with mediating effects, 41 of which showed mediation, direct, and total effects (Table S14). For example, the abundance of *CAG-791 sp902780385* and starch degradation III was associated with increased serum INS levels. Conversely, the increased

abundance of *Treponema-D sp017381365* and glycolysis III as well as *Alistipes sp015059845* and gluconeogenesis I reduce serum TG and INS levels (Fig. 9h).

Discussion

To our knowledge, this study is the first to investigate the gut microbiomes of periparturient dairy cows using a large-scale, high-frequency sampling approach. We also uniquely employed a combination of microbial clustering, ecological model, and random forest analysis to reveal that the rumen and fecal microbiome succession in periparturient dairy cows can be divided into three distinct stages. The results showed significant differences in the composition and function of rumen and fecal microbiota across different successional stages. Moreover, the transitions between these stages were driven not only by different taxa but also significantly influenced metabolic phenotype changes in periparturient dairy cows.

As expected, we observed significant changes in the microbial structure of the rumen and feces in periparturient dairy cows. However, compared to previous studies, the trends in Chao1 and Shannon indices of the rumen and fecal microbiomes in our study showed unique characteristics [23-26]. Notably, these previous studies were conducted on the same breed of cows with similar periparturient diets—high-fiber diets before calving and high-starch diets postpartum. This underscores the importance of large sample sizes and high temporal resolution in revealing changes in gut microbiome. Furthermore, we found that individual differences had an even greater impact on gut microbiome structure throughout the periparturient period than changes observed within individuals over time, indicating periparturient dairy cow trials should increase sample size to mitigate the impact of inter-individual variability on experimental outcomes, thereby effectively controlling the occurrence of false positive errors. Importantly, we identified three deterministic successional stages in both the rumen (rapid transition, transition, stabilization) and feces (stabilization, transition, stabilization), revealing the compartmentalized adaptive strategies of gut microbial communities to periparturient stress. Rumen microbiota were more sensitive to stress, while fecal microbiota exhibited a delayed response, a characteristic also observed in the study by Bach *et al.* [24]. Typically, the most severe negative energy balance in dairy cows occurs within the first two weeks after calving and gradually alleviates as the days in milk increasing. This aligns with the stabilization of the ruminal microbiota beginning at 14 days postpartum, indicating that the microbial community restructuring is an active participant in metabolic recovery, not just a bystander. During the

ruminal stabilization stage, the dominant *Succiniclasticum* is associated with the conversion of succinate to propionate, a critical pathway for gluconeogenesis during negative energy balance. In contrast, the early stabilization of the feces (at 10 days postpartum) coincides with the peak incidence of metabolic diseases [5, 29], suggesting that the fecal microbiota may prioritize immune homeostasis over energy acquisition [30].

Additionally, we innovatively used ecological models to estimate the assembly processes of rumen and fecal microbiota in dairy cows. The divergent assembly mechanisms of the rumen microbiota (dominated by HOS) and the fecal microbiota (dominated by DL) during succession highlight compartment-specific selection pressures. HOS suggests that under environmental pressure, microbial community compositions tend to become homogeneous, emphasizing the dominant role of environmental factors in shaping microbial community structures [31, 32]. In contrast, DL refers to changes in the relative abundance of species in the microbial community due to random events (including random deaths or births), implying that ecological niches in the community are not completely occupied or utilized [33, 34]. *Succiniclasticum* is not only the dominant genus during the rumen stabilization stage but also actively responds to the HOS process within the rumen microbial community. Previous studies have already confirmed the impact of diet on community succession in dairy cows [16]. Combined with the linear increase in feed intake of postpartum cows [35], this may reflect the selective pressure imposed by the host through pH shifts and the influx of dietary starch, which also favors the growth of acid-tolerant taxa such as *Prevotella*. In contrast, the DL-driven pattern in the feces indicates that neutral processes dominate, likely due to physical niche partitioning (e.g., mucus layer gradients) that limit microbial dispersal [36]. Notably, *Bifidobacterium*, as a key DL-associated taxon, may occupy unutilized niches by producing substances such as acetate, which helps maintain gut homeostasis and inhibit pathogens [37-40]. These findings align with metacommunity theory, where rumen microbiota resemble a "species-sorting" model, while fecal communities follow "neutral assembly". These findings further support the results of Shen *et al*. [41], which suggested that the fecal microbiome in ruminants more accurately reflects the host health status compared to rumen microbiome.

Our covariate analysis revealed that gut microbial succession is shaped by a complex interplay of temporal, nutritional, and host-specific factors, with distinct drivers dominating different successional

stages. Notably, sire effect dominates in RS2, which may imply that the assembly process of the periparturient rumen microbiota is influenced by genetic factors. Parallel patterns were observed in fecal microbiota, where CD and diet drove early succession (FS1), while sire effect predominated in later stages (FS3), reinforcing the temporal hierarchy of environmental versus host-intrinsic influences across gut niches. The MaAsLin2 analysis further highlighted functional linkages between key taxa (e.g., *Succiniclasticum*, *Prevotella*) and critical parameters like CD and pH, suggesting these microbes may serve as biological integrators of postpartum physiological changes and dietary adaptation. Importantly, the decoupled nature of rumen and fecal microbial ecosystems underscores their compartmentalized contributions to host metabolism. Despite minimal direct microbial exchange, both ecosystems exerted comparable influence on blood metabolic profiles (~20% variance) through distinct pathways. Rumen microbial transformation types preferentially modulated energy mobilization markers (NEFA, GLU) and growth factors (IGF-1), aligning with its role as the primary nutrient-processing organ. Conversely, fecal microbiota dynamics showed stronger associations with lipid metabolism regulators (TG, BHBA) and insulin signaling, potentially reflecting downstream metabolic byproduct processing or gut barrier function interactions. These compartment-specific metabolic fingerprints emphasize the need for ecosystem-targeted interventions during the transition period. The stage-specific dominance of sire-related effect across both gut sites raises intriguing questions about heritable microbial transmission mechanisms and their potential for selective breeding strategies. previous research on humans, pigs, sheep, and beef cattle has demonstrated significant correlations between host SNPs and their gut microbiome [42-45], underscoring the potential of genetic breeding for altering the gut microbiota composition of periparturient dairy cows to reduce the incidence of metabolic diseases. However, the limited explanatory power of measured factors for certain key taxa suggests unaccounted variables, possibly including microbial cross-talk or host epigenetic regulation, warranting further investigation.

We further revealed fundamental differences in the functional trajectories and metabolic specialization of the periparturient rumen and fecal microbiomes through metagenomic and network analyses, emphasizing their compartmentalized yet coordinated roles in shaping host metabolic adaptation. The rumen microbial communities prioritize nutrient extraction and energy flux (e.g., pyruvate fermentation and glycolysis in RS1; the Bifidobacterium shunt in RS3), while the fecal microbiota

specialize in substrate salvage and metabolic byproduct processing (e.g., sulfate reduction in FS1; D-galacturonate degradation in FS3). These observations are consistent with the anatomical and physiological roles of each gut compartment: the rumen rapidly adapts to postpartum dietary shifts and lactation-driven energy demands, while the feces fine-tunes downstream metabolic pathways to manage residual substrates and systemic metabolic stress. The stage-specific complexity of microbial networks further supports the dichotomy: simpler interactions in the stable phases of the fecal microbiota suggest a homeostatic "maintenance" state, while the dynamic succession stages of the rumen exhibit enriched pathways for polyamine and coenzyme A synthesis, likely reflecting their roles in sustaining microbial turnover and redox balance during metabolic upheaval. Notably, key taxa such as *Prevotella* and *Succiniclasticum* in the rumen, and *Bifidobacterium* and *Treponema* in the feces, emerge as central network nodes, acting as linchpins that connect microbial community structure with metabolic output. Their prominence in energy-yielding processes (glycolysis, mixed-acid fermentation) and biosynthesis (nucleotides, amino acids) positions these taxa as critical regulators of host–microbe metabolic crosstalk. The mediation models further demonstrate the potential mechanisms by which key microbes regulate host metabolic phenotypes. For instance, the glucose-related pathway in both the rumen and feces mediates the impact of key microbes on host metabolism, highlighting the crucial role of glucose supplementation in postpartum diets for maintaining dairy cow health. Previous studies have linked *Alistipes* to short-chain fatty acid (SCFA) production, including acetate and propionate, and its reduced abundance is associated with disease progression, including non-alcoholic fatty liver disease and non-alcoholic steatohepatitis, owing to decreased SCFA levels [46, 47]. As we all know, postpartum dairy cows are susceptible to fatty liver. Thus, *Alistipes sp015059845* in the feces may play a vital role in maintaining liver and intestinal health in postpartum dairy cows. Additionally, the elevated abundance of *UBA3839 sp900314125* associated with Glycolysis I and *UBA2813 sp902801985* associated with L-lysine biosynthesis I in the rumen mediated the reduction of serum NEFA levels, indicating complex interplay between microbial metabolism and host energy regulation in periparturient dairy cows. *UBA3839 sp900314125*, through its association with Glycolysis I, may promote the production of propionate, a key precursor for gluconeogenesis [48]. Increased availability of propionate enhances hepatic glucose synthesis, thereby raising blood glucose levels and subsequently boosting insulin signaling. This cascade inhibits lipolysis

in adipose tissue by suppressing hormone-sensitive lipase, thereby reducing the release of NEFA into the bloodstream [49]. Meanwhile, *UBA2813 sp902801985* associated with L-lysine biosynthesis I may optimize nitrogen utilization and energy efficiency within the gut microbiota. As an essential amino acid, lysine not only supports protein synthesis but may also modulate host metabolic pathways such as AMPK or mTOR signaling to enhance glucose uptake and utilization, further reducing reliance on fat mobilization [50]. Overall, we propose that modulating the fecal microbiota may be more beneficial in preventing postpartum metabolic diseases in dairy cows, as its transition period precedes the typical onset of such conditions. These results enhance our understanding of the spatiotemporal coordination of gut microbial ecosystems during metabolic stress in periparturient dairy cows and provide a framework for developing targeted management strategies to optimize cow health and productivity during critical transition periods.

This study is the first to comprehensively provide a dynamic perspective on the gut microbiome of periparturient dairy cows, enhancing our understanding of the dynamic interactions between the gut microbiome and the host during the periparturient period. Since this study only examined healthy cows, our next research objective is to compare the microbiomes of healthy and diseased cows to explore mechanisms related to postpartum metabolic disorders. Additionally, this study lacks information on feed intake, which is a critical factor influencing gut microbiome composition. In future research, we plan to investigate the impact of feed intake on the dynamic changes in the gut microbiome of periparturient dairy cows. Moreover, based on the patterns observed in this study, further research is needed to verify whether modifying the succession process of the gut microbiome by targeting the identified key microbes and pathways can effectively regulate host metabolism in periparturient cows, thereby preventing postpartum metabolic disorders.

Materials and Methods

Animals, experimental design, and sample collection

This study was conducted at a Commercial Dairy Farm in Shanxi Province, China. The dairy cows involved in the experiment were managed using a traditional feeding model. Two months before the expected calving date, the cows were moved to a dry cow barn and started on a high-fiber total mixed

ration (TMR) (dry period) diet. After calving, the cows were immediately separated from their calves to prevent further exposure of the dams to microbes from the calves. The cows were then moved to a transition barn where professional technicians immediately milked the colostrum and fed them a fresh cow diet (high-starch TMR). Three days after calving, the cows were transferred to a fresh barn until the end of the experiment. In the fresh barn, the cows were allowed access to the same high-starch TMR and water *ad libitum* during the experiment. Details on TMR are provided in Table S15. The postpartum care was given in the transition barn within 1–2 days, which included feeding an oral bolus (Bovikalc bolus, Boehringer Ingelheim, MO, USA) containing $CaCl_2$ and $CaSO_4$ (43 g of Ca), measuring rectal temperature (M900 Thermometer, GLA Agricultural Electronics, Inc., CA, USA), and drenching 300 mL liquid propylene glycol orally using a drench gun.

The study included 211 healthy, multiparous, pregnant dairy cows. During the experiment, an experienced veterinarian conducted health assessments, including evaluations of body condition, rectal temperature, blood BHB concentration, and mental state. Table S16 presents the health assessment criteria. After excluding cows with abnormal conditions and those treated with medication during the experimental period to avoid interference with the generalizability of our results, 91 normal peripartum cows were included (Fig. 1). The strict control of normal cows allowed us to minimize interference from other macro factors, providing a clearer view of the natural dynamic changes in the gut microbiome and key factors driving these changes.

Rumen fluid, fecal, and blood samples were collected from these cows 21 days before calving (expected calving date) and on days 1, 3, 7, 14, and 21 after calving (Fig. 1). The means and standard deviations (SDs) of the actual sampling day on day -21 were day -19.29 ± 4.70 (min ~ max, -31 ~ -6). Regardless of the exact hour of the calving day, samples of day 1 were collected on the day following parturition. All samples were collected before the morning feeding on the designated sampling day. Rumen fluid samples were collected using a special rumen tube (Metal Systems, Kiryat Gat, Israel) designed based on the physiological structure of adult cows to ensure that the tube reached the ventral aspect of the rumen. The exterior metal of the rumen tube was polished to minimize damage to the esophagus and rumen. Fecal samples were obtained from the rectum of cows by research personnel wearing sterile long-arm gloves. Blood samples were collected from the caudal veins of cows in 10 ml

vacuum blood collection tubes containing EDTA (0102-1032, Kangjian Medical Supplies Co., Ltd, Jiangsu, China). Rumen fluid and fecal samples were immediately transferred to 2 ml cryogenic tubes and stored in liquid nitrogen until subsequent bacterial diversity analysis. Blood samples were centrifuged at 3,000 × *g* and 4°C for 15 min to obtain the plasma, which was then transferred to 2-ml cryogenic tubes and stored in liquid nitrogen for subsequent analysis of energy metabolism, liver function, and antioxidant indicators.

Plasma parameters measurement

GLU, TG, aspartate aminotransferase (AST), and alanine aminotransferase (ALT) concentrations in dairy plasma were determined using an automatic biochemistry analyzer (CLS880; Ze Cheng Biotechnology Co., Ltd., Jiangsu, China). INS levels were quantified via radioimmunoassays using a multitube counter (BFM-96; Zhongcheng Technology, Hefei, China). Direct field assessment of BHB levels was performed in freshly collected blood samples using specific portable test strips (Nova Vet; Nova Biomedical Corporation, Waltham, MA, USA). Serum concentrations of NEFA (Cat# A042-1-1), IGF-1 (Cat# H041-1-2), total antioxidant capacity (T-AOC; Cat# A015-1-2), and haptoglobin (HP; Cat# H136) were quantified using commercial enzyme-linked immunosorbent assay (ELISA) kits (Nanjing Jiancheng Bioengineering Institute, Nanjing, China). Briefly, following the manufacturer's protocol, serum samples were thoroughly mixed with the reagents provided in the kit, incubated at 37°C, and then analyzed using a microplate reader to measure absorbance values, with target concentrations calculated based on the standard curve. The RBHB index, calculated as 1/[log glucose (mg/dl)+log insulin (μU/ml)+log NEFA (mmol/l)+log BHB (mmol/l)], was used to evaluate insulin resistance in dairy cows based on a previously described method [51].

Microbial DNA extraction

Total genomic DNA was extracted from all samples using the DNeasy PowerSoil Pro Kit 47014 (Qiagen, Hilden, Germany), according to the manufacturer's instructions, and stored at -20°C for subsequent analysis. DNA quantity and quality were assessed using a NanoDrop NC2000 spectrophotometer (RRID:SCR_018042, Thermo Fisher Scientific, Waltham, MA, USA) and agarose gel

electrophoresis, respectively.

16S rRNA gene sequencing

The 16S rRNA gene was amplified using universal primers (341F: 5′-CCTACGGGNGGCWGCAG-3′; 805R: 5′-GACTACHVGGGTATCTAATCC-3′) targeting the V3-V4 region, with 7-bp barcodes added for multiplex sequencing. Each PCR mixture contained 5 µl buffer (5×), 0.25 µl Fast pfu DNA Polymerase (5 U/µl), 2 µl (2.5 mM) dNTPs, 1 µl (10 µM) of each forward and reverse primer, 1 µl DNA template, and 14.75 µl ddH2O. Thermal cycling steps involved an initial denaturation step at 98°C for 5 min, followed by 25 cycles of denaturation at 98°C for 30 s, annealing at 53°C for 30 s, and extension at 72°C for 45 s, with a final extension at 72°C for 5 min. The PCR amplicons were purified using Vazyme VAHTSTM DNA Clean Beads (Vazyme, Nanjing, China) and quantified using the Quant-iT PicoGreen dsDNA Assay Kit (Invitrogen, Carlsbad, CA, USA). After each quantification step, the amplicons were pooled in equal amounts, and paired-end 2×250-bp sequencing was performed using the Illumina NovaSeq platform with the NovaSeq 6000 SP Reagent Kit (500 cycles) at Shanghai Personal Biotechnology Co., Ltd (Shanghai, China).

Data quality control and analyses were performed using the QIIME2 pipeline (RRID:SCR_021258) with slight modifications according to official tutorials [52]. Briefly, raw sequence data were demultiplexed using the demux plugin, followed by primer cutting using the cutadapt plugin [53]. The sequences were then quality filtered, denoized, merged, and chimeras were removed using the DADA2 plugin (RRID:SCR_023519) [54]. Non-singleton ASVs were aligned using mafft, and a phylogeny tree was constructed using fasttree2 [55, 56]. ASVs were taxonomically classified using the classify-sklearn naive Bayes taxonomy classifier in the feature-classifier plugin against the SILVA Release 138.1 database [57].

Metagenomic sequencing

We conducted metagenomic analyses on samples randomly selected from 10 cows in the rumen and feces at three successional stages (N=60). The extracted total DNA was processed using the Illumina TruSeq Nano DNA LT Library Preparation Kit (Illumina, USA) to construct metagenomic shotgun

sequencing libraries with an insert length of approximately 400 bp. Each library was sequenced on the Illumina NovaSeq™X Plus platform (RRID:SCR_024568, Illumina, USA) and Personal Biotechnology Co., Ltd (Shanghai, China) using the PE150 strategy. For metagenomic data processing, Cutadapt (v1.2.1; RRID:SCR_011841)was used to remove sequencing adapters from the raw reads [58]. Low-quality reads were trimmed using a sliding window algorithm in fastp (v0.23.2) [59]. Reads were aligned to the bovine genome using Minimap2 (v2.24-f1122, RRID:SCR_018550) to remove host contamination [60]. Subsequently, Kaiju (v1.9.0) was used to classify metagenomic reads against the GTDB-derived database (v207) for each sample [61]. Reads assigned to Metazoa or Viridiplantae were excluded from downstream analysis. Megahit (v1.1.2, RRID:SCR_018551) was used with the "-k-list 33,55,77,99,127 -min-contig-len 300" to assemble reads in each sample [62]. Contigs generated were clustered using the "easy-linclust" mode of MMseqs2 (v15, RRID:SCR_022962) with a sequence identity threshold of 0.95 and a 90% coverage of the shorter contigs [63]. Genes were predicted using Prodigal (v2.6.3, RRID:SCR_011936) [64]. The CDS sequences from all samples were clustered using the "easy-cluster" mode of Mmseqs2, with a protein sequence identity threshold of 0.95 and 90% coverage of shorter sequences. Reads were then mapped to the predicted gene sequences using Minimap2, and featureCounts was used to calculate the number of reads aligned to each gene [65]. Abundance was expressed in TPM (Transcripts Per Million). The functional annotation of non-redundant genes was performed using the "search" mode of Mmseqs2 against the Metacyc database (RRID:SCR_007778) [66].

Bioinformatics and statistical analysis

After obtaining the ASV datasets of the rumen and fecal microbiome, they were processed using the "phyloseq" package in the R software (4.2.2), with a rarefaction depth set to the minimum sample sequence quantity. The ASV datasets were independently subsetted for individual analysis of the rumen and fecal microbiome. Conversely, for integrated analysis of the rumen and fecal microbiome, combined ASV datasets were aggregated. The downstream analysis of 16S sequencing data mainly includes: individual variation, DMM, α-diversity, β-diversity, LEfSe, Markov chain, random forest, ICAMP, EnvFit, MaAsLin2, Procrustes, and source tracking analysis. For examining the α-diversity, ASV-level indices, including Chao1 richness and Shannon diversity index, were calculated using the "vegan" package in R

[67, 68]. β-diversity was explored using Bray–Curtis dissimilarity metrics to understand the structural variations in microbial communities across samples using the “vegan” package in R [69]. A DMM model was applied at the genus level to cluster samples based on the microbial community structure [14, 70], with clusters determined according to the lowest Laplace approximation score [70]. This analysis was performed independently for rumen (RDMM) and fecal samples (FDMM). Linear discriminant analysis effect size (LEfSe, LDA>4, $p<0.05$) was used to identify the dominant taxa across the groups [71]. Transition dynamics between these states were analyzed via Markov chain models using the Markov chain packages in R [72], following the methodology by Xiao *et al*. [15]. Models predicting microbial stages of the rumen and fecal microbiome were constructed using random forest algorithms with ASVs that showed more than 0.1% relative abundance, employing the “randomForest” package in R [73]. To reduce model overfitting, five 10-fold cross-validations were conducted using 70% of samples for model building and 30% as the test set. The area under the receiver operating characteristic curve (AUC) was calculated using R. ICAMP was performed using a galaxy-based pipeline to assess the relative importance of deterministic and stochastic processes in bacterial community assembly [74]. The observed taxa were first grouped into bins (“boxes”) based on their phylogenetic relationships, with the minimum number of taxa per bin as the default setting (bin.size.limit=24). Although the main function within icamp.big was used to calculate the within-bin β-nearest taxon index (βNTI), the modified Raup-Crick metric (RC) was used to evaluate the relative importance of different ecological processes within each bin. For each bin, pairwise comparisons with βNRI < -1.96 are considered to be controlled by homogeneous selection, while those with βNRI $> +1.96$ are controlled by heterogeneous selection. Next, other processes are categorized using the phylogenetic diversity metric RC with |βNRI| ≤ 1.96. When RC < -0.95, it is considered as a process of homogeneous dispersal, while RC $> +0.95$ indicates a process of dispersal limitation. Cases with |βNRI| ≤ 1.96 and |RC| ≤ 0.95 represent the influence of processes such as drift. EnvFit analysis was conducted to examine the relationship between individual factors (eg., diet, sire, birth weight, predelivery - actual days) and microbial succession stages, using the “vegan” package in R. Correlational analyses of individual factors and genera, with a cow as a random effect and false discovery rate (FDR) adjustment using the Benjamini-Hochberg method, were performed using microbiome multivariate association with linear models (MaAsLin2) [75]. Procrustes analysis between the rumen and fecal ASVs was conducted

using the "protest" function in the R package vegan [76]. To further determine the relationship between rumen and fecal microbiomes, fecal microbiome source tracking was conducted using the sourcetracker2 plugin (https://github.com/caporaso-lab/sourcetracker2), which can assess the proportion of fecal microbiome originating from rumen microbiome at the same time point and fecal microbiome at previous time points. Residuals between rumen or fecal samples and blood indicators were generated using the "Procrustes" function in the R package vegan [17].

Species analysis of metagenomic sequencing data is based on read count data, while functional analysis is based on TPM datasets. The downstream analyses mainly include: β-diversity of species and microbial functions, co-occurrence network analysis of species, network analysis, differential analysis, and mediation analysis. β-diversity at the species level, including bacteria, eukaryotes, and archaea, was assessed using Bray–Curtis dissimilarity. The co-occurrence networks were constructed based on SparCC correlation coefficients (|R| > 0.6, $p < 0.05$) using top 500 most abundant genera [77]. Co-occurrence networks and node topology were evaluated to examine interspecies interactions and network centrality [78]. Functional β-diversity was assessed at the pathway level of the Metacyc database using Bray–Curtis dissimilarity. For differential analysis of species, taxa with a prevalence greater than 50% and relative abundance greater than 0.1% were selected. For differential analysis of pathways, taxa with a prevalence greater than 50% and relative abundance greater than 0.01% were selected. Mediation analysis was used to determine whether the effects of species on host metabolic indicators are mediated by microbial functions, using the "mediation" package in R.

β-diversity was evaluated using PERMANOVA with 999 permutations and visualized using principal coordinate analysis (PCoA) [79]. Genus diversity under different RDMM or FDMM conditions is represented via heatmaps using the ComplexHeatmap package in R [80]. Sample distributions at each peripartum time point and succession patterns for RDMM or FDMM are depicted using pie charts and Sankey diagrams (https://sankeymatic.com/build/), respectively. Markov chain model was visualized using the igraph packages in R [72]. Intraindividual and interindividual compositional variabilities were calculated according to the method by Olsson *et al.* [81]. Intraindividual compositional variability was defined as the median Bray–Curtis dissimilarity calculated between samples from a cow (i.e., 20 dissimilarity values were calculated for the six samples obtained from each cow). Interindividual

compositional variability was defined as the median Bray–Curtis dissimilarity calculated for six samples from a cow against all other samples. Additional visualizations were created using ggplot2 in R. All differential analyses were performed using Kruskal–Wallis followed by Dunn's *post hoc* tests using the "Kruskal.test" and "dunn.test" functions in R (dunn.test package). Statistical significance was set at $p < 0.05$.

Availability of source code and requirements

Not applicable.

Data Availability

The metagenomic and 16S rRNA sequencing supporting this work have been archived in the NCBI database under accession numbers PRJNA1161368 and PRJNA1126601, respectively. Other data and files further supporting this work are openly available in the *GigaScience* repository, GigaDB [82].

Abbreviations

ASV, amplicon sequence variant; DMM, Dirichlet multinomial mixture; RDMM, DMM clusters for rumen microbiota; FDMM, DMM clusters for fecal microbiota; FDR, false discovery rate; HOS, homogeneous selection; DL, dispersal limitation; LEfSe, Linear discriminant analysis effect size; PERMANOVA, permutational multivariate analysis of variance; GLU, glucose; TG, triglycerides, AST, aspartate aminotransferase; ALT, alanine aminotransferase; BHB, β-hydroxybutyrate; RBHB, revised quantitative insulin sensitivity check index-β-Hydroxybutyrate; INS, Insulin; NEFA, non-esterified fatty acids; IGF-1; insulin-like growth factor 1; T-AOC; total antioxidant capacity; and HP, haptoglobin; ELISA, enzyme-linked immunosorbent assay; βNTI, β-nearest taxon index; modified RC, Raup-Crick metric; ICAMP, Phylogenetic bin-based null model analysis; TMR, total mixed ration

Declarations

Ethics Approval

The study design was approved by the Institutional Experimental Animal Care and Use Committee of the

Ministry of Agriculture and Rural Affairs of China and the Animal Care and Use Committee at China Agricultural University (approval number: AW01103202-1-31).

Consent for publication

Not applicable

Competing Interests

The authors declare that they have no conflict of interest.

Funding

This study was supported by the National Natural Science Foundation of China (grant number 32130100).

Author Contributions

Conceptualization: S.W., F.K., S.L., W.W.

Methodology: S.W., F.K., S.L.

Investigation: S.W., F.K., D.D., C.L.

Visualization: S.W., F.K.

Supervision: S.L., W.W.

Writing—original draft: S.W., F.K.

Writing—review & editing: S.W., F.K., Y.H., E.W., Z.C., Y.W., S.L., W.W

References

[1]Joachim von Braun KA, Louise O. Fresco, and Mohamed Hassan. Food systems: seven priorities to end hunger and protect the planet. Nature 2021; 597:28-30.

[2]Grummer RR. Impact of changes in organic nutrient metabolism on feeding the transition dairy cow. Journal of animal science 1995; 73:2820-2833.

[3]Goff JP, Horst RL. Physiological changes at parturition and their relationship to metabolic disorders. J Dairy Sci 1997; 80:1260-1268.

[4]Bizelis J, Charismiadou M, Rodkais E. Metabolic changes during the perinatal period in dairy sheep in relation to level of nutrition and breed. II. Early lactation. Journal of Animal Physiology and Animal Nutrition 2000; 84:73-84.

[5]Bruckmaier R, Gross JJ. Lactational challenges in transition dairy cows. Animal Production Science 2017; 57:1471-1481.

[6]Esposito G, Irons PC, Webb EC, Chapwanya A. Interactions between negative energy balance, metabolic diseases, uterine health and immune response in transition dairy cows. Anim Reprod Sci 2014; 144:60-71.

[7]Cainzos JM, Andreu-Vazquez C, Guadagnini M, Rijpert-Duvivier A, Duffield T. A systematic review of the cost of ketosis in dairy cattle. J Dairy Sci 2022; 105:6175-6195.

[8]Cammack KM, Austin KJ, Lamberson WR, Conant GC, Cunningham HC. RUMINANT NUTRITION SYMPOSIUM: Tiny but mighty: the role of the rumen microbes in livestock production. J Anim Sci 2018; 96:752-770.

[9]O'Hara E, Neves AL, Song Y, Guan LL. The role of the gut microbiome in cattle production and health: driver or passenger? Annual review of animal biosciences 2020; 8:199-220.

[10]Xue M, Sun H, Wu X, Guan LL, Liu J. Assessment of Rumen Microbiota from a Large Dairy Cattle Cohort Reveals the Pan and Core Bacteriomes Contributing to Varied Phenotypes. Appl Environ Microbiol 2018; 84.

[11]Shabat SK, Sasson G, Doron-Faigenboim A, Durman T, Yaacoby S, Berg Miller ME et al. Specific microbiome-dependent mechanisms underlie the energy harvest efficiency of ruminants. Isme j 2016; 10:2958-2972.

[12]Ley RE, Peterson DA, Gordon JI. Ecological and evolutionary forces shaping microbial diversity in the human intestine. Cell 2006; 124:837-848.

[13]Oliphant K, Parreira VR, Cochrane K, Allen-Vercoe E. Drivers of human gut microbial community assembly: coadaptation, determinism and stochasticity. The ISME journal 2019; 13:3080-3092.

[14]Stewart CJ, Ajami NJ, O'Brien JL, Hutchinson DS, Smith DP, Wong MC et al. Temporal development of the gut microbiome in early childhood from the TEDDY study. Nature 2018; 562:583-588.

[15]Xiao L, Wang J, Zheng J, Li X, Zhao F. Deterministic transition of enterotypes shapes the infant gut

microbiome at an early age. Genome Biol 2021; 22:243.

[16]Furman O, Shenhav L, Sasson G, Kokou F, Honig H, Jacoby S et al. Stochasticity constrained by deterministic effects of diet and age drive rumen microbiome assembly dynamics. Nature Communications 2020; 11.

[17]Feng Y, Zhang M, Liu Y, Yang X, Wei F, Jin X et al. Quantitative microbiome profiling reveals the developmental trajectory of the chicken gut microbiota and its connection to host metabolism. iMeta 2023; 2:e105.

[18]Wang X, Tsai T, Deng F, Wei X, Chai J, Knapp J et al. Longitudinal investigation of the swine gut microbiome from birth to market reveals stage and growth performance associated bacteria. Microbiome 2019; 7:1-18.

[19]Chai J, Zhuang Y, Cui K, Bi Y, Zhang N. Metagenomics reveals the temporal dynamics of the rumen resistome and microbiome in goat kids. Microbiome 2024; 12:14.

[20]Yan X, Si H, Zhu Y, Li S, Han Y, Liu H et al. Integrated multi-omics of the gastrointestinal microbiome and ruminant host reveals metabolic adaptation underlying early life development. Microbiome 2022; 10:222.

[21]Malmuthuge N, Liang G, Guan LL. Regulation of rumen development in neonatal ruminants through microbial metagenomes and host transcriptomes. Genome Biol 2019; 20:172.

[22]Tröscher-Mußotter J, Saenz JS, Grindler S, Meyer J, Kononov SU, Mezger B et al. Microbiome clusters disclose physiologic variances in dairy cows challenged by calving and lipopolysaccharides. Msystems 2021; 6:10.1128/msystems. 00856-00821.

[23]Zhu Z, Noel SJ, Difford GF, Al-Soud WA, Brejnrod A, Sorensen SJ et al. Community structure of the metabolically active rumen bacterial and archaeal communities of dairy cows over the transition period. PLoS One 2017; 12:e0187858.

[24]Bach A, López-García A, González-Recio O, Elcoso G, Fàbregas F, Chaucheyras-Durand F et al. Changes in the rumen and colon microbiota and effects of live yeast dietary supplementation during the transition from the dry period to lactation of dairy cows. Journal of Dairy Science 2019; 102:6180-6198.

[25]Zhu SL, Gu FF, Tang YF, Liu XH, Jia MH, Valencak TG et al. Dynamic fecal microenvironment

properties enable predictions and understanding of peripartum blood oxidative status and nonesterified fatty acids in dairy cows. J Dairy Sci 2024; 107:573-592.

[26]Luo Z, Du Z, Huang Y, Zhou T, Wu D, Yao X et al. Alterations in the gut microbiota and its metabolites contribute to metabolic maladaptation in dairy cows during the development of hyperketonemia. mSystems 2024; 9:e0002324.

[27]Arshad MA, Hassan F-u, Rehman MS, Huws SA, Cheng Y, Din AU. Gut microbiome colonization and development in neonatal ruminants: Strategies, prospects, and opportunities. Animal Nutrition 2021; 7:883-895.

[28]Xu Q, Qiao Q, Gao Y, Hou J, Hu M, Du Y et al. Gut microbiota and their role in health and metabolic disease of dairy cow. Frontiers in nutrition 2021; 8:701511.

[29]LeBlanc S. Monitoring metabolic health of dairy cattle in the transition period. Journal of reproduction and Development 2010; 56:S29-S35.

[30]Cani PD, Delzenne NM. The role of the gut microbiota in energy metabolism and metabolic disease. Current pharmaceutical design 2009; 15:1546-1558.

[31]Hernandez DJ, David AS, Menges ES, Searcy CA, Afkhami ME. Environmental stress destabilizes microbial networks. The ISME Journal 2021; 15:1722-1734.

[32]Sharma A, Richardson M, Cralle L, Stamper CE, Maestre JP, Stearns-Yoder KA et al. Longitudinal homogenization of the microbiome between both occupants and the built environment in a cohort of United States Air Force Cadets. Microbiome 2019; 7:1-17.

[33]Wright RJ, Gibson MI, Christie-Oleza JA. Understanding microbial community dynamics to improve optimal microbiome selection. Microbiome 2019; 7:1-14.

[34]George AB, Korolev KS. Ecological landscapes guide the assembly of optimal microbial communities. PLOS Computational Biology 2023; 19:e1010570.

[35]Roche JR, Friggens NC, Kay JK, Fisher MW, Stafford KJ, Berry DP. Invited review: Body condition score and its association with dairy cow productivity, health, and welfare. Journal of dairy science 2009; 92:5769-5801.

[36]Zhou J, Ning D. Stochastic community assembly: does it matter in microbial ecology? Microbiology and Molecular Biology Reviews 2017; 81:10.1128/mmbr. 00002-00017.

[37]Fan Q, Wanapat M, Yan T, Hou F. Altitude influences microbial diversity and herbage fermentation in the rumen of yaks. BMC Microbiol 2020; 20:370.

[38]Xiong Y, Wang X, Li X, Guo L, Yang F, Ni K. Exploring the rumen microbiota of Hu lambs in response to diet with paper mulberry. Appl Microbiol Biotechnol 2023; 107:4961-4971.

[39]Sasajima N, Ogasawara T, Takemura N, Fujiwara R, Watanabe J, Sonoyama K. Role of intestinal Bifidobacterium pseudolongum in dietary fructo-oligosaccharide inhibition of 2, 4-dinitrofluorobenzene-induced contact hypersensitivity in mice. British journal of nutrition 2010; 103:539-548.

[40]Sun D, Bian G, Zhang K, Liu N, Yin Y, Hou Y et al. Early-life ruminal microbiome-derived indole-3-carboxaldehyde and prostaglandin D2 are effective promoters of rumen development. Genome Biology 2024; 25:64.

[41]Shen H, Lu Z, Xu Z, Chen Z, Shen Z. Associations among dietary non-fiber carbohydrate, ruminal microbiota and epithelium G-protein-coupled receptor, and histone deacetylase regulations in goats. Microbiome 2017; 5:1-12.

[42]Li F, Li C, Chen Y, Liu J, Zhang C, Irving B et al. Host genetics influence the rumen microbiota and heritable rumen microbial features associate with feed efficiency in cattle. Microbiome 2019; 7:1-17.

[43]Wang W, Zhang Y, Zhang X, Li C, Yuan L, Zhang D et al. Heritability and recursive influence of host genetics on the rumen microbiota drive body weight variance in male Hu sheep lambs. Microbiome 2023; 11:197.

[44]Chen L, Wang D, Garmaeva S, Kurilshikov A, Vich Vila A, Gacesa R et al. The long-term genetic stability and individual specificity of the human gut microbiome. Cell 2021; 184:2302-2315.e2312.

[45]Yang H, Wu J, Huang X, Zhou Y, Zhang Y, Liu M et al. ABO genotype alters the gut microbiota by regulating GalNAc levels in pigs. Nature 2022; 606:358-367.

[46]Zhu L, Baker SS, Gill C, Liu W, Alkhouri R, Baker RD et al. Characterization of gut microbiomes in nonalcoholic steatohepatitis (NASH) patients: a connection between endogenous alcohol and NASH. Hepatology 2013; 57:601-609.

[47]Wu D, Liu L, Jiao N, Zhang Y, Yang L, Tian C et al. Targeting keystone species helps restore the dysbiosis of butyrate-producing bacteria in nonalcoholic fatty liver disease. iMeta 2022; 1:e61.

[48]Koh A, De Vadder F, Kovatcheva-Datchary P, Bäckhed F. From dietary fiber to host physiology: short-chain fatty acids as key bacterial metabolites. Cell 2016; 165:1332-1345.

[49]Den Besten G, Van Eunen K, Groen AK, Venema K, Reijngoud D-J, Bakker BM. The role of short-chain fatty acids in the interplay between diet, gut microbiota, and host energy metabolism. Journal of lipid research 2013; 54:2325-2340.

[50]Cheng S, Ma X, Geng S, Jiang X, Li Y, Hu L et al. Fecal microbiota transplantation beneficially regulates intestinal mucosal autophagy and alleviates gut barrier injury. Msystems 2018; 3:10.1128/msystems. 00137-00118.

[51]Cai J, Zhao FQ, Liu JX, Wang DM. Local Mammary Glucose Supply Regulates Availability and Intracellular Metabolic Pathways of Glucose in the Mammary Gland of Lactating Dairy Goats Under Malnutrition of Energy. Front Physiol 2018; 9:1467.

[52]Bolyen E, Rideout JR, Dillon MR, Bokulich NA, Abnet CC, Al-Ghalith GA et al. Reproducible, interactive, scalable and extensible microbiome data science using QIIME 2. Nat Biotechnol 2019; 37:852-857.

[53]Kechin A, Boyarskikh U, Kel A, Filipenko M. cutPrimers: A New Tool for Accurate Cutting of Primers from Reads of Targeted Next Generation Sequencing. J Comput Biol 2017; 24:1138-1143.

[54]Callahan BJ, McMurdie PJ, Rosen MJ, Han AW, Johnson AJ, Holmes SP. DADA2: High-resolution sample inference from Illumina amplicon data. Nat Methods 2016; 13:581-583.

[55]Katoh K, Misawa K, Kuma K, Miyata T. MAFFT: a novel method for rapid multiple sequence alignment based on fast Fourier transform. Nucleic Acids Res 2002; 30:3059-3066.

[56]Price MN, Dehal PS, Arkin AP. FastTree 2--approximately maximum-likelihood trees for large alignments. PLoS One 2010; 5:e9490.

[57]Bokulich NA, Kaehler BD, Rideout JR, Dillon M, Bolyen E, Knight R et al. Optimizing taxonomic classification of marker-gene amplicon sequences with QIIME 2's q2-feature-classifier plugin. Microbiome 2018; 6:90.

[58]Martin M. Cutadapt removes adapter sequences from high-throughput sequencing reads. EMBnet journal 2011; 17:10-12.

[59]Chen S, Zhou Y, Chen Y, Gu J. fastp: an ultra-fast all-in-one FASTQ preprocessor. Bioinformatics 2018; 34:i884-i890.

[60]Li H. Minimap2: pairwise alignment for nucleotide sequences. Bioinformatics 2018; 34:3094-3100.

[61]Menzel P, Ng KL, Krogh A. Fast and sensitive taxonomic classification for metagenomics with Kaiju. Nature communications 2016; 7:11257.

[62]Li D, Liu C-M, Luo R, Sadakane K, Lam T-W. MEGAHIT: an ultra-fast single-node solution for large and complex metagenomics assembly via succinct de Bruijn graph. Bioinformatics 2015; 31:1674-1676.

[63]Steinegger M, Söding J. MMseqs2 enables sensitive protein sequence searching for the analysis of massive data sets. Nat Biotechnol 2017; 35:1026-1028.

[64]Hyatt D, Chen GL, Locascio PF, Land ML, Larimer FW, Hauser LJ. Prodigal: prokaryotic gene recognition and translation initiation site identification. BMC Bioinformatics 2010; 11:119.

[65]Liao Y, Smyth GK, Shi W. featureCounts: an efficient general purpose program for assigning sequence reads to genomic features. Bioinformatics 2014; 30:923-930.

[66]Caspi R, Billington R, Keseler IM, Kothari A, Krummenacker M, Midford PE et al. The MetaCyc database of metabolic pathways and enzymes - a 2019 update. Nucleic Acids Res 2020; 48:D445-d453.

[67]Chao A. Nonparametric Estimation of the Number of Classes in a Population. Scandinavian Journal of Statistics 1984; 11:265-270.

[68]Shannon CE. A mathematical theory of communication. The Bell system technical journal 1948; 27:379-423.

[69]Bray JR, Curtis JT. An ordination of the upland forest communities of southern Wisconsin. Ecological monographs 1957; 27:326-349.

[70]Holmes I, Harris K, Quince C. Dirichlet multinomial mixtures: generative models for microbial metagenomics. PloS one 2012; 7:e30126.

[71]Segata N, Izard J, Waldron L, Gevers D, Miropolsky L, Garrett WS et al. Metagenomic biomarker

discovery and explanation. Genome biology 2011; 12:1-18.
[72]Han W-S, Lee J, Pham M-D, Yu JX. iGraph: a framework for comparisons of disk-based graph indexing techniques. Proceedings of the VLDB Endowment 2010; 3:449-459.
[73]Rigatti SJ. Random forest. Journal of Insurance Medicine 2017; 47:31-39.
[74]Ning D, Yuan M, Wu L, Zhang Y, Guo X, Zhou X et al. A quantitative framework reveals ecological drivers of grassland microbial community assembly in response to warming. Nat Commun 2020; 11:4717.
[75]Mallick H, Rahnavard A, McIver LJ, Ma S, Zhang Y, Nguyen LH et al. Multivariable association discovery in population-scale meta-omics studies. PLoS computational biology 2021; 17:e1009442.
[76]Andreella A, Finos L. Procrustes Analysis for High-Dimensional Data. Psychometrika 2022; 87:1422-1438.
[77]Friedman J, Alm EJ. Inferring correlation networks from genomic survey data. PLoS Comput Biol 2012; 8:e1002687.
[78]Csardi G, Nepusz T. The igraph software. Complex syst 2006; 1695:1-9.
[79]McArdle BH, Anderson MJ. Fitting multivariate models to community data: a comment on distance-based redundancy analysis. Ecology 2001; 82:290-297.
[80]Gu Z. Complex heatmap visualization. Imeta 2022; 1:e43.
[81]Olsson LM, Boulund F, Nilsson S, Khan MT, Gummesson A, Fagerberg L et al. Dynamics of the normal gut microbiota: a longitudinal one-year population study in Sweden. Cell host & microbe 2022; 30:726-739. e723.
[82] Wang S; Kong F; Dai D; Li C; Hao Y; Wang E; Cao Z; Wang Y; Wang W; Li S: Supporting data for "Deterministic Succession Patterns in the Rumen and Fecal Microbiome Associated with Metabolic Shifts in Pertiparturient Dairy Cattle" GigaScience Database. 2025. https://doi.org/10.5524/102686

Figures

Fig. 1. Profiling gut microbiome changes in periparturient dairy cows and their connection to host metabolism: Workflow. BHB: β-hydroxybutyrate.

Fig. 2. Classification of the rumen and fecal microbiome in periparturient dairy cows based on microbial community clusters. Boxplots showing intra- and interindividual Bray–Curtis dissimilarity in microbiome profiles: (a) Rumen and (f) feces. The PCoA plot-based Bray–Curtis dissimilarity of microbiome profiles across different DMMs: (b) rumen and (g) feces. DMM represents a microbial community cluster. Ring charts showing the distribution of DMMs, with the numbers in the middle of the ring charts representing the sampling time points: (c) rumen and (h) feces. LEfSe reveals the key genera of different DMMs: (d) rumen and (i) feces. Temporal changes of Shannon index in each DMM: (e) rumen and (j) feces. DMM, Microbial cluster.

Fig. 3. Temporal dynamics of ruminal DMMs in periparturient dairy cows. (a) The Sanky diagram shows the transition of ruminal DMMs across six sampling time points. (b) Markov chain with subject-independent transition probabilities among ruminal DMMs, in which arrow weights are proportional to the maximum likelihood estimate of the transition probabilities among different states. Ruminal DMMs in different colored boxes are in different stages of microbial succession. The numbers represent the conversion rates across different ruminal DMMs. The numbers represent the conversion rates across different ruminal DMMs. (c) Receiver operating characteristic curve demonstrating the accuracy of the classification model for the successional stages in the rumen microbiome of periparturient dairy cows. DMM, Microbial cluster.

Fig. 4. Temporal dynamics of fecal DMMs in periparturient dairy cows. (a) The Sanky diagram shows the transition of fecal DMMs across six sampling time points. (b) Markov chain with subject-independent transition probabilities among fecal DMMs, in which arrow weights are proportional to the maximum likelihood estimate of the transition probabilities among different states. Fecal DMMs in different colored boxes are in different stages of microbial succession. The numbers represent the conversion rates across different fecal DMMs. The numbers represent the conversion rates across different fecal DMMs. (c)

Receiver operating characteristic curve demonstrating the accuracy of the classification model for the successional stages in the fecal microbiome of periparturient dairy cows. DMM, Microbial cluster.

Fig. 5. Ecological assembly mechanism of rumen microbiome in periparturient dairy cows. (a) Relative importance of HOS and DL processes in the rumen microbiome at different successional stages in periparturient cows. (b) Differences in ecological processes among different phylogenetic groups (the relative abundance of the top20 Bins) in the rumen. The different colors of the inner and outer circles represent the phylum affiliations of the Bins and the ecological processes driven by the Bins, respectively. (c) Stacked plot showing the relative abundance of the top20 Bins in different rumen stages. (d) Relative contribution of the top20 Bins to DL and HOS processes in different rumen stages. (e) The relative abundance of representative genera of Bins contributing to succession at different rumen stages. HOS, Homogeneous selection; DL, Dispersal limitation; DMM, Microbial cluster.

Fig. 6. Ecological assembly mechanism of the fecal microbiome in periparturient dairy cows. (a) Relative importance of HOS and DL processes in the fecal microbiome at different successional stages in periparturient cows. (b) Differences in ecological processes among different phylogenetic groups (the relative abundance of the top20 Bins) in the feces. The different colors of the inner and outer circles represent the phylum affiliations of the Bins and the ecological processes driven by the Bins, respectively. (c) Stacked plot showing the relative abundance of the top20 Bins in different fecal DMMs. (d) Relative contribution of the top20 Bins to DL and HOS processes in different fecal DMMs. (e) The relative abundance of representative genera of Bins contributing to succession at different fecal stages. HOS, Homogeneous selection; DL, Dispersal limitation; DMM, Microbial cluster; RS1, 2, 3, rumen succession stages 1, 2, 3; FS1, 2, 3, fecal succession stages 1, 2, 3.

Fig. 7. Significance and explained variance of 18 microbiome covariates modelled by EnvFit across all data types. Horizontal bars show the amount of variance (r^2) explained by each covariate in the model as determined by EnvFit. Significant covariates ($p < 0.05$) are represented in bold font. P-A: Predelivery - Actual; BW: Birth weight; DIM: Days in milk; NM: Number of matings; CD: Calving to days; MY:

Milk yeild; RPH: Ruminal pH; BCS: Body Condition Score; RT: Rectal temperature; FPH: Fecal pH; CP: Crude protein; ADF: Acid detergent fiber; NDF: Neutral detergent fiber

Fig. 8. Analysis of the relationship between the rumen and fecal microbiome as well as effect of succession types on host metabolic phenotype in periparturient cows. (a) Procrustes analysis of the correlation between ruminal and fecal microbiome based on the Bray–Curtis dissimilarity of ASVs (M^2=0.79, p<0.01, 999 permutations). (b) Source tracking of the fecal microbiome in periparturient cows. Fecal samples from the former sampling time point and rumen samples from the same sampling time point were considered potential sources of feces at this time point in the same cow. (c) Residuals showing the difference in the microbe–host association from rumen and feces with relative abundance. Microbial succession types and statistics: (d) rumen and (f) feces. Boxplot showing the significance test of effect of succession types on host metabolic phenotype (only significant combinations are shown): (e) rumen and (g) feces. RS1, 2, 3, rumen succession stages 1, 2, 3; FS1, 2, 3, fecal succession stages 1, 2, 3; S1-S1, the microbial stage of the individual transitions from S1 to S1; RBHB, revised quantitative insulin sensitivity check index-β-Hydroxybutyrate; BHB, β-hydroxybutyrate; HP, haptoglobin; T-AOC, total antioxidant capacity; AST, aspartate aminotransferase; ALT, alanine aminotransferase; IGF-1, insulin-like growth factor 1; NEFA, non-esterified fatty acids; TG, triglycerides; INS, Insulin

Fig. 9. Analysis of microbiome–host interactions in periparturient cows. (a) The PCoA based on species-level Bray–Curtis dissimilarity shows differences in rumen and fecal microbiome profiles across succession stages. (b) The co-occurrence network diagram displays the SparCC interaction relationships of the top 500 abundant genera at different rumen of fecal succession stages (R> 0.6; p< 0.05). (c) Topological analysis of the co-occurrence network. (d) The PCoA analysis based on Bray–Curtis dissimilarity of pathways reveals distinct microbial functional profiles in the rumen and feces across different successional stages. Heatmap showing the significantly different metabolic pathways (normalized) of the microbiome at different succession stages in periparturient cows: (e) rumen and (f) feces. Part of mediation linkages among the species, pathways, and metabolic phenotype: (e) rumen and (f) feces. RS1, 2, 3, rumen succession stages 1, 2, 3; FS1, 2, 3, fecal succession stages 1, 2, 3; NEFA, non-

esterified fatty acids; TG, triglycerides; INS, Insulin

Supplementary Figures

Figs. S1 to S4:

Fig. S1. Dynamics and diversity of rumen microbiota during the periparturient period. (a) PCoA plot-based Bray–Curtis dissimilarity showing rumen microbiota profiles across different sampling time points. (b) Dynamic changes in the α-diversity of rumen microbiota at different periods during the perinatal period. (c) Plots showing a comparison of the five bacterial phyla with the highest relative abundance in the rumens of perinatal cows. (d) Laplace approximation scores for ruminal DMM. (e) The top 20 taxa that contributed the most to the accuracy of the rumen DMM are shown in the order of importance. (f) Heatmap showing the relative abundance of the top 20 taxa (normalised in each taxon) in each rumen sample.

Fig. S2. Dynamics and diversity of fecal microbiota during the periparturient period. (a) PCoA plot-based Bray–Curtis dissimilarity showing fecal microbiota profiles across different sampling time points. (b) Dynamic changes in the α-diversity of fecal microbiota at different time points during the perinatal period. (c) Plots showing a comparison of the five bacterial phyla with the highest relative abundance in the fecal microbiota of perinatal cows. (d) Laplace approximation scores for fecal DMM. (e) The top 20 taxa that contributed the most to the accuracy of the fecal DMM are shown in the order of importance. (f) Heatmap showing the relative abundance of the top 20 taxa (normalised in all taxa) in each fecal sample.

Fig. S3. Cross-validation of ASVs contributing to the random forest model. (a) Rumen. (b) Feces. ASV, Amplicon sequence variant

Fig. S4. Relative importance of different ecological processes in the microbiota composition of periparturient dairy cows: (a) Rumen. (b) Feces.

Supplementary Tables

Tables S1 to S18:

Table S1. Details of transformation of ruminal DMMs at different time points.

Table S2. Details of transformation of fecal DMMs at different time points.

Table S3. Details of ruminal Bins in ICAMP assembly.

Table S4. Details of fecal Bins in ICAMP assembly.

Table S5. MaAsLin2 method was used to investigate the relationship between individual factors and key genera driving succession in dairy cows.

Table S6. Analysis of the contribution of rumen and fecal microbiota to blood metabolism.

Table S7. Node analysis of co-occurrence network in RS1 stage.

Table S8. Node analysis of co-occurrence network in RS2 stage.

Table S9. Node analysis of co-occurrence network in RS3 stage.

Table S10. Node analysis of co-occurrence network in FS1 stage.

Table S11. Node analysis of co-occurrence network in FS2 stage.

Table S12. Node analysis of co-occurrence network in FS3 stage.

Table S13. Mediation analysis of ruminal different species, ruminal different pathways, and metabolic phenotype.

Table S14. Mediation analysis of fecal different species, fecal different pathways, and metabolic phenotype.

Table S15. Feed ingredients and nutrient composition of diets.

Table S16. Summary of reasons for elimination.

Table S17. The rumen samples corresponding to each cow.

Table S18. The fecal samples corresponding to each cow.

Figure3 Click here to access/download;Figure;Figure 3.tif

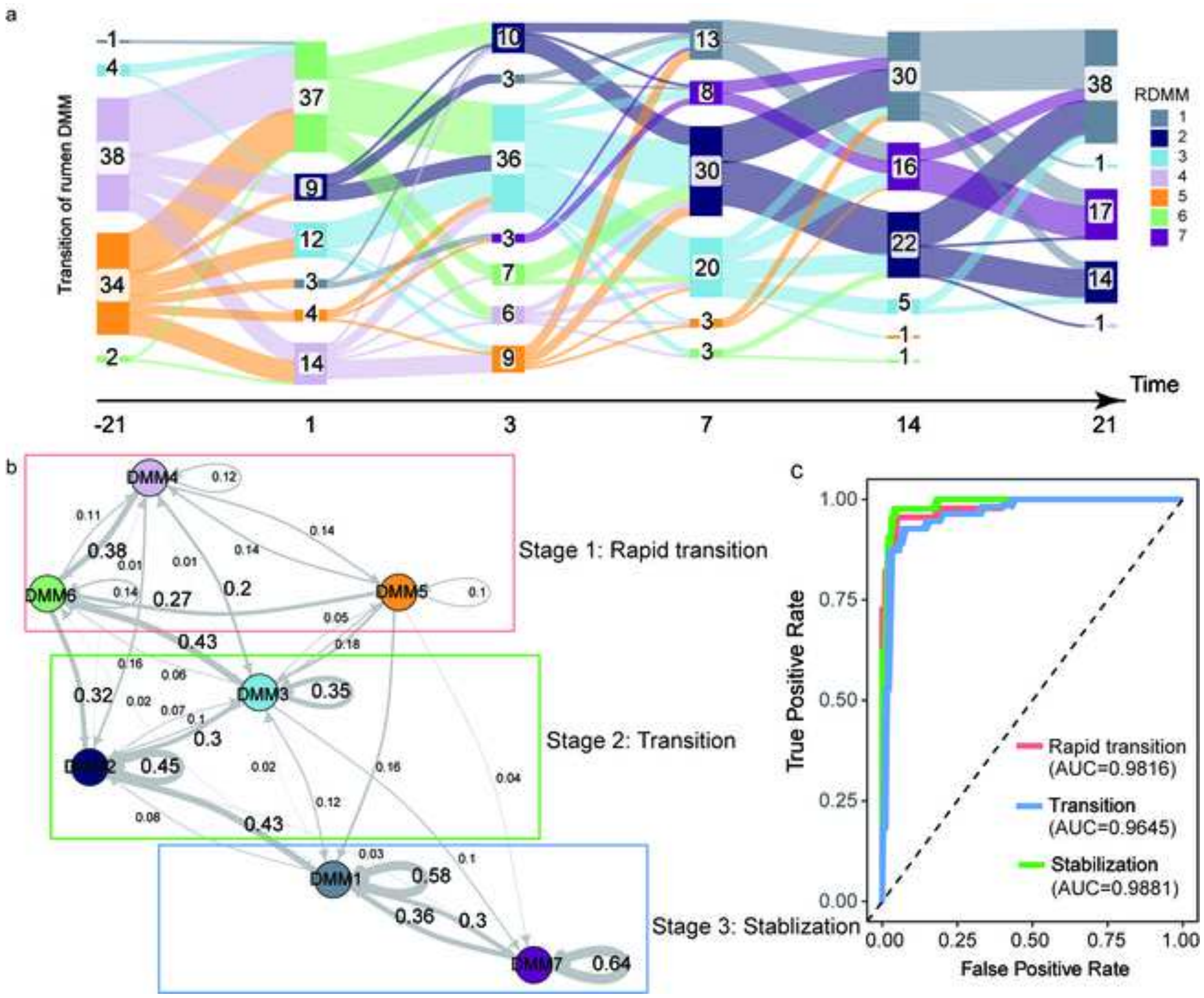

Figure4

Click here to access/download;Figure;Figure 4.tif

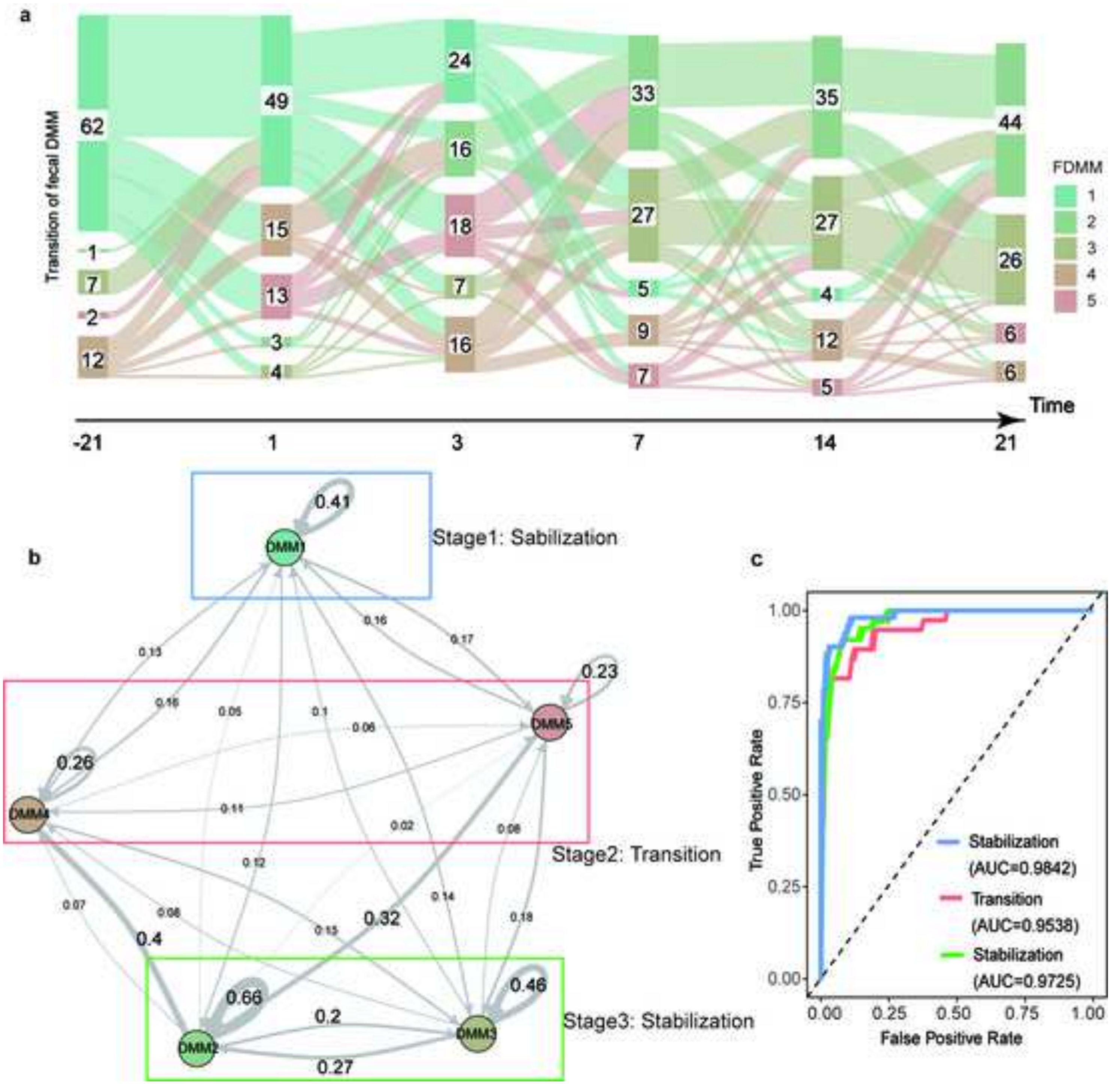

Figure7

Click here to access/download;Figure;Figure 7.tif

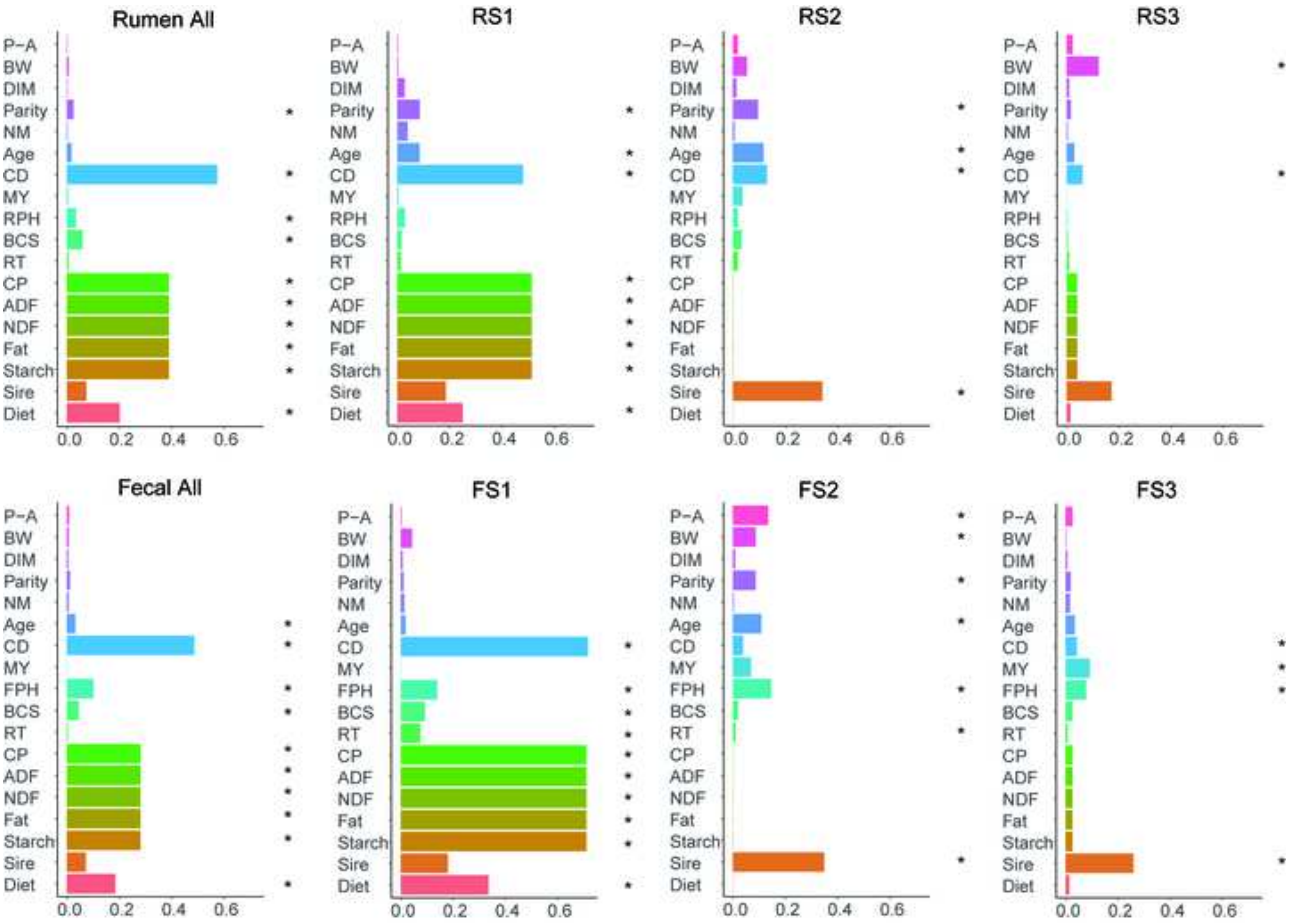

Response

Click here to access/download
Supplementary Material
Respones.docx

Click here to access/download
Supplementary Material
Supplementary Figures.docx

Click here to access/download
Supplementary Material
Supplementary Tables.xlsx
