# Supplementary material for: Deterministic succession patterns in the rumen and fecal microbiome associate with host metabolic shifts in peripartum dairy cattle
Source: Gigascience. 2025 May 19;14:giaf042. doi: 10.1093/gigascience/giaf042 (PMC12087452; doi:10.1093/gigascience/giaf042)
Supplement: giaf042_Authors_Response_To_Reviewer_Comments_original_submission [file giaf042_authors_response_to_reviewer_comments_original_submission.pdf]

Author's Response To Reviewer Comments

Response to the editor and reviewers

February 27th, 2025

Manuscript ID: GIGA-D-24-00404
Title: Deterministic Succession Patterns of Rumen and Hindgut Microbiome Driving Host Metabolism in Periparturient Ruminants: Insights from a Large-Scale Longitudinal Study of Cows

Dear editor and reviewers:

Thank you for your constructive comments. We have made the following corrections in accordance with your valuable suggestions.

Comments from Reviewers:
Reviewer #1: Thanks to all author for the excellent study
Response: Thank you very much for your positive evaluation of our work. We will address your comments one by one.

Comment 1: It was good to see if the authors collected sample 7 day of pre-partum because 21 days of prepartum is a long time which is hard to estimate the changes over 21-day prepartum to 0 day.
Response: Thank you for your professional insights. During the study design phase, we did consider including the 7-day prepartum period (-7 d) as a key sampling point. However, practical implementation revealed significant limitations in this sampling window due to biological variations in individual calving times (with some cows even entering the periparturient period up to 15 days earlier than anticipated):
(1) Although population averages exist, the intense metabolic fluctuations occurring during this narrow -7 d window (proximal to calving) could compromise data accuracy if these samples were included.
(2) Prepartum cows represent a vulnerable population, and sampling at -7 d might impose unnecessary stress on both dams and offspring.
(3) The postpartum period constitutes the high-risk phase for bovine diseases, thus omitting the -7d sampling point does not diminish the clinical relevance of this investigation.
This protocol modification aligns with the 3R principles (Replacement, Reduction, Refinement) of animal research ethics while enabling precise monitoring of dynamic metabolic adaptations during the critical postpartum period, thereby ensuring high-value research outcomes.

Comment 2: It is not clearly mentioned in the methodology which sequencing data used for which analysis.
Response: As suggested, we added the relevant content in the Bioinformatics and statistical analysis section has been described in detail. (Lines 564-592 and Lines 602-614)

Comment 3: Did author observe any correlation between provided food and changes in rumen and hindgut microbiome
Response: In fact, we used the EnvFit and MassLine2 methods to analyze the correlations between diet type, components, and the microbiome. Details can be found in the section "Contribution of multiple individual factors to gut microbial succession " and Fig.7.

Comment 4: Title "Deterministic Succession Patterns of Rumen and Hindgut Microbiome Driving Host Metabolism in Periparturient Ruminants" is not clearly presenting the study. I would suggest modifying.
Response: As suggested, we modified to 'Deterministic Succession Patterns in the Rumen and Fecal Microbiome Associate with host Metabolic Shifts in Periparturient Dairy Cattle'. The title not only highlights the main findings but also aligns with Reviewer 2's emphasis on the correlation and the need to avoid redundancy in the title.

Comment 5: Line 23: which platform of metagenome sequencing
Response: As suggested, we added the information of platform. (Line 22)

Comment 6: Line 36: Significantly (which test)
Response: Our differential analysis was performed using the Kruskal-Wallis (KW) test, as mentioned in the section on statistical methods. (Line 625)

Comment 7: Line 51: please provide exact definition of periparturient period with reference
Response: As suggested, we added the reference. (Line 49)
Grummer R. R. (1995). Impact of changes in organic nutrient metabolism on feeding the transition dairy cow. Journal of animal science, 73(9), 2820–2833. https://doi.org/10.2527/1995.7392820x

Comment 8: Line 63: Sentence is not complete
Response: As suggested, we revised the relative sentence. (Lines 62-66)

Comment 9: Line 64: over “the” time
Response: The relevant statement has been removed in the latest revision.

Comment 10: Line 69: to understand
Response: As suggested, we modified the problem. (Line 68)

Comment 11: Line 71-75: Provide more example of cattle and cow. Study on pregnant women is not relating the earlier sentence.
Response: As suggested, we have removed the references about pregnant women and added references related to dairy cows. To avoid redundancy in citations, we have included only the representative articles published in Nature Communications. (Line 73-75)

Comment 12: Line: 97-107: these are the part of methodology. I will suggest avoiding in introduction section.
Response: As suggested, we rewrote the relevant content. (Lines 99-102)

Comment 13: Line: 115: Which timepoint or days
Response: As suggested, we revised the relevant content. (Lines 109-112)

Comment 14: Line: 117: Which type of drastic change, decrease or increase
Response: In accordance with the suggestions from Reviewer 2, we have revised it to "significant change." The primary purpose of this revision is to highlight the emergence of significant changes, as the significant changes in the rumen and feces are opposite to each other. This can be observed in the supplementary Figures. (Line 112)

Comment 15: Line: 124: which results?
Response: In accordance with the suggestions from Reviewer 2, we have incorporated this result into Fig. S1A and Fig. S2A.

Comment 16: Line133: Please describe clearly the pattern of genera depending on the pre and postpartum stages. RDMM1 prominently appeared until 7 d postpartum and what in 14 and 21 days of postpartum? Similarly, RDMM2 and RDMM7 were increasing with the timepoints of post-partum.
Response: As suggested, we revised the relevant content. (Lines 128-142)

Comment 17: Line 140: use past tense and over “the” time and full stop at the end.
Response: As suggested, we revised the relevant problem. (Line 143)

Comment 18: Line 141-142: Remove third bracket
Response: As suggested, we revised the relevant problem. (Line 141)

Comment 19: Line 141: RDMM7 started to appear on 3 d postpartum and gradually increase over the timepoints.

Response: As suggested, we revised the relevant problem. (Line 141-142)

Comment 20: Line 143-145: It is not clear to me. What does timeline of dominance shift mean?
Response: As suggested, we revised the relevant problem. (Line 143)

Comment 21: Line 146: What type of instability observed?
Response: As suggested, we revised the relevant problem. (Line 146)

Comment 22: Line 147: periparturient age points?
Response: As suggested, we revised the relevant problem. (Line 147)

Comment 23: Line 151: "Similarly, FDMMs exhibited distinct occurrence windows and dominant genera (Figs. 2h and i)" sentence is not complete, occurrence based on?
Response: As suggested, we revised the relevant problem. (Lines 150-151)

Comment 24: Line 154: disappeared "at" postpartum
Response: As suggested, we revised the relevant problem. (Line 154)

Comment 25: Line 155: after postpartum or with postpartum.
Response: As suggested, we added the 'with' (Line 155)

Comment 26: Line 155: "with FDMM2 being characterized by Rikenellaceae_RC9_gut_group, Muribaculaceae, and Alistipes, and FDMM3 by Bacteroidales_RF16_group and Prevotellaceae_UCG_003" use separate sentence
Response: As suggested, we separated the sentence. (Lines 155-157)

Comment 27: Line 159: with a peak at 3 d postpartum
Response: As suggested, we revised the relevant problem. (Lines 159)

Comment 28: Line 151-160: clearly mention which figures are related. Are those lines not related with figure 2h if yes then which figure is related with community structure. Please make sure figures are mentioned clearly after each description or before description
Response: To avoid redundancy in labeling, we have added the annotation on Line 151.

Comment 29: Line 163-164: RDMM and FDMM are not dominated with similar type of genus in each cluster, so comparison between them has no meaning
Response: Thank you for the suggestion. We have removed the phrase "Unlike in the rumen." （Line 162）

Comment 30: Line 167: Did you include all 91 animals?
Response: Yes, we have added the '91'. (Line 168)

Comment 31: Line 175-176: Please mention the percentage of transformation over the time.
Response: As suggested, we added the relative content. (Lines 173-177)

Comment 32: Line 180: How is it suggesting a return to stability?
Response: We have removed this inferential statement from the results section to ensure an objective description of the findings.

Comment 33: Line 182: What does "microbial community types" mean?
Response: We have revised "microbial community types" to "microbial clusters." (Line 182)

Comment 34: Line 185: What are self-transition rates?
Response: The self-transition rates refer to the probability that the DMM remains unchanged at the next time point. For example, the probability that DMM1 on day 1 postpartum is still DMM1 on day 3 postpartum.

Comment 35: Line 195-196: "previously mentioned genera, including Rikenellaceae_RC9_gut_group,

Prevotella, Acetitomaculum, and F082” this line is not clear.
Response: As suggested, we revised the relevant problem. (Lines 195-197)

Comment 36: Line 201-203: this could be the part of discussion.
Response: Thank you for the suggestion. We have removed this part from the results section.

Comment 37: Line 208: In RDMM analysis, author used top100 and in FDMM top 120 ASVs-is there any reason of this change?
Response: This is the result after model optimization based on five 10-fold cross-validations, and the relevant details have been described in the Materials and Methods section. （Lines 576-578）

Comment 38: Line 220: both rumen and faeces.
Response: As suggested, deleted ‘the’ (Line 219)

Comment 39: Line 222-223: Sentence is not clear.
Response: We have split this sentence into two to improve readability. (Line 218-222)

Comment 40: Line 268: What does last time point, please specify.
Response: We revised it to "the previous sampling timepoint" to enhance readability. (Line 267)

Comment 41: Line 273: What does “residuals” mean, please define.
Response: Thank you for your question. The “residuals” refer to the differences between the observed values of the dependent variable the values predicted by our statistical model. These residuals represent the portion of the data that is not explained by the model.

Comment 42: Line 320: “Systematically investigate” is that correct terminology to describe the trial?
Response: Thank you for the suggestion. We have removed the word "Systematically" to increase the accuracy of the statement.

Comment 43: Line 336-338: Avoid repetition of the methodology.
Response: Thank you very much for the suggestion. In the latest version, we have removed this sentence.

Comment 44: Line 379: Affect
Response: Thank you very much for the suggestion. In the latest version, we have removed this sentence.

Comment 45: Line 381: Stage means post-partum stage?
Response: One of the main findings of this study is the identification of three deterministic successional stages in both the rumen (rapid transition, transition, stabilization) and feces (stabilization, transition, stabilization) of periparturient dairy cows. In the rumen, Stage 1 transitions directly to Stage 3 (stabilization) without passing through the intermediate transition stage. For more details, please refer to Fig. 3, Fig. 4, and Fig. 8.

Comment 46: 427: typo, bran or barn?
Response: Thank you very much, the problem has been made. (Line 462)

Comment 47: 443: Specification of the tube.
Response: As suggested, we added the information. (Lines 185-187)

Comment 48: 449: Did author used similar method and kit of DNA extraction from rumen and faeces samples.
Response: Yes, it is the same. We have added "from all samples." (Line 510)

Comment 49: 511: RDMM and FDMM clusters were defined based on 16S sequencing or metagenomics data? And what is the lowest Laplace approximation score?
Response: In the DMM model analysis using 16S data, the Laplace approximation score is used to evaluate the goodness of fit for models with different numbers of components. It is a key indicator for determining the optimal number of microbial communities. Consistent with common information criteria (such as

AIC/BIC), a lower Laplace score indicates a better model fit.

Comment 50: 536: What does R threshold mean?
Response: As suggested, the wording has been revised. (Lines 606-607)

Comment 51: 539-540: the sentence is not clear
Response: As suggested, the wording has been revised. (Lines 610-612)

Comment 52: 550: specification of each kit
Response: As suggested, we added the information. (Lines 499-502)

Comment 53: 551: Briefly describe the procedure of ELISA briefly.
Response: As suggested, we added the information. (Lines 502-505)

Comment 54: 571: Which individual factors?
Response: As suggested, we added the information. (Lines 519-512)

Comment 55: 572: How the false discovery rate adjusted?
Response: Thank you for your question regarding the adjustment of the false discovery rate (FDR). In our study, we used the Benjamini-Hochberg method to adjust the FDR. (Line 594)

Comment 56: Figure 7: In x axis did not use the same value which affecting the Stack bars. I will suggest y to make similar values in x-axis.
Response: As suggested, we have revised Figure 7.

Comment 57: Figure 3b and 4b: What does three stage means and how they defined “stabilization, transition and stabilization”?
Response: We defined it based on the results of the Markov chain analysis. For details, see the Results section (Lines 182-197).

Reviewer #2: This study provides a comprehensive longitudinal analysis of rumen and hindgut microbiome dynamics in periparturient dairy cows, linking microbial succession patterns to host metabolic phenotypes. The work is well-structured, methodologically rigorous, and addresses a critical gap in understanding how gut microbiota transitions during the periparturient period influence metabolic health. The integration of high-resolution temporal sampling, ecological modeling, and mediation analysis strengthens the novelty and impact of the findings. However, some aspects of experimental design, statistical reporting, and interpretation require clarification or expansion before publication.
Response: Thank you very much for your positive evaluation of our work. We will address your comments one by one.

Comment 1: Why were rumen and fecal samples studied separately when analyzing the dynamic succession of the gut microbiota and its relationship with host metabolism in periparturient cows? Not as a whole. Please clarify.
Response: In the study of ruminants, rumen and fecal samples were analyzed separately, primarily because they represent distinct microbial niches and functions. The rumen, as the core site of foregut fermentation, hosts microbial communities (such as fiber-degrading bacteria and volatile fatty acid–producing microbes) that directly dominate energy supply, which is especially crucial during the periparturient period when energy demands surge. In contrast, fecal microbiota reflect the residual microbial communities after the entire digestive process, including digestion, absorption, and metabolism, and are more associated with hindgut immune regulation and metabolic waste processing. Additionally, rumen microbiota responds more rapidly to changes in diet and hormones, while fecal microbiota, due to the longer gut transit time, may exhibit lagged dynamics. Separate analyses can precisely distinguish their independent contributions to host metabolism (such as energy balance and inflammation regulation), avoid the mixing of functional signals, and provide a scientific basis for differentiated intervention strategies targeting the rumen or hindgut (such as probiotic addition and dietary adjustment).

Comment 2: The causal inference that "microbial communities drive host metabolic changes" requires

cautious phrasing. Although mediation analysis was used to infer microbial-driven metabolic alterations, the observational nature of the study inherently limits the ability to exclude confounding factors, rendering causal claims speculative. The authors must revise such statements to avoid overstating conclusions.
Response: Thank you for the reviewer's suggestions. We have avoided such expressions in the title, abstract, and discussion sections to enhance the rigor of the study. (Title, line 30 and34, Discussion)

Comment 3: For host indicators, please explain why only energy metabolism, liver function, and antioxidant indicators were analyzed.
Response: In the study of periparturient dairy cows, energy metabolism, liver function, and antioxidant indicators are typically analyzed with priority, as they are directly related to the core physiological challenges of this period. Energy metabolism indicators (such as NEFA and BHBA) reflect the negative energy balance caused by the onset of lactation and reduced feed intake, which can warn of metabolic risks such as ketosis. Liver function indicators (AST and ALT) assess the liver's overload in carbohydrate and lipid metabolism and detoxification, revealing potential damage such as fatty liver. Antioxidant and stress indicators (T-AOC and HP) monitor the level of oxidative stress, and their imbalance can exacerbate tissue damage and immune suppression. These three categories of indicators have recently been considered interrelated and together form the key targets for monitoring periparturient metabolic health, providing a basis for early intervention.

Comment 4: Key methodological details are missing, hindering reproducibility. For example, descriptions of ICAMP and mediation analysis are insufficient
Response: In accordance with your suggestions and those of Reviewer 1, we have revised the Materials and Methods section to enhance the reproducibility and readability of this study. (Lines 559-614)

Comment 5: The Discussion lacks depth. Expand the interpretation of microbial succession patterns and host-microbe interactions observed in the results.
Response: As suggested, the discussion section has been rewritten to provide in-depth explanations of the key findings. (Discussion section)

Comment 6: Title: Overly verbose. Shorten to emphasize the core focus and novelty. Replace "Periparturient Ruminants" with "Periparturient Dairy Cows" for precision
Response: As suggested, we revised the title

Comment 7: Line 24: Revise "ecological modeling" to "ecological model" for grammatical consistency.
Response: As suggested, we have revised the problem. (Line 23)

Comment 8: Abbreviations: Define "RQUICKI-BHB" in full upon first mention: Revised Quantitative Insulin Sensitivity Check Index-BHB. Provide the full name for "MaAsLin2": Multivariate Analysis by Linear Models. Ensure all abbreviations (e.g., TMR, DMM, ICAMP) are defined at their first occurrence.
Response: Thank you for the suggestion. We have revised the entire manuscript to address the problems.

Comment 9: Line 117: Replace the colloquial phrase "drastic changes" with "significant changes" for scientific rigor.
Response: As suggested, we have revised the problem. (Line 112)

Comment 10: Line 123: Explicitly annotate the PCoA plot to display the variance percentages (26.14% and 24.96%).
Response: As suggested, we have incorporated this result into Fig. S1A and Fig. S2A.

Comment 11: Line 124: The sentence "These results highlight their individualized states..." is interpretive and belongs in the Discussion, not Results.
Response: The relevant statement has been removed from the results section.

Comment 12: Line 126 Justify the use of the Dirichlet Multinomial Mixture (DMM) model instead of the more common Partitioning Around Medoids (PAM) method for clustering.
Response: In microbiome data analysis, the Dirichlet Multinomial Mixture (DMM) model has significant advantages over Partitioning Around Medoids (PAM), especially when dealing with high-dimensional, sparse

discrete data (such as OTU/ASV count data). The DMM, based on the Dirichlet Multinomial distribution, can directly model the compositional nature of microbiome data, capturing its heterogeneity and complex community structure. In contrast, PAM, as a distance-based clustering method, typically assumes continuous data and struggles to handle the characteristics of discrete data directly. Moreover, DMM does not require pre-specifying the number of clusters and can automatically determine the optimal number through model selection methods (such as BIC or AIC), making it suitable for exploratory analyses. In contrast, PAM requires manually specifying the number of clusters, which may introduce subjective bias. DMM is also less sensitive to outliers and provides probabilistic interpretability that can be directly linked to the biological significance of microbiome data, making it particularly suitable for analyzing dynamic changes in time-series data. In comparison, PAM lacks probabilistic interpretability and has weaker capabilities for capturing dynamic changes. Therefore, in the dynamic analysis of the periparturient dairy cow microbiome, the DMM model is a more appropriate choice, especially when capturing complex community structures and dynamic changes is required.

Comment 13: Terminology clarity: Replace "DMM" with "cluster" throughout the text to improve readability, unless the method itself is a key focus.
Response: Thank you very much for your suggestions. During the manuscript writing process, we also paid attention to the consistency of terminology and readability. As you pointed out, DMM not only represents the results of clustering but also reflects the uniqueness of the methodology. To ensure that readers can understand clearly, we provided a detailed definition of DMM when it was first mentioned in the results section, and subsequently used “DMM” or “cluster” appropriately according to the context to balance professionalism and readability.

Comment 14: Line 420-428 Periparturient cows usually receive postpartum care. What is the care plan in this experiment?
Response: Thank you for the reminder We have added the relevant content. (Lines 163-167)

Comment 15: Line 434 Clarify whether all 91 cows completed sampling at all six time points. Provide details on sample loss rates or exclusion criteria
Response: Thank you very much for your suggestion. In this experiment, to ensure the accuracy of the results, we ensured that each cow had at least four samples for rumen or feces. The detailed information on sample missingness has been included in the supplementary tables, where blanks indicate missing samples for a particular cow. (Table S17 and S18)

Comment 16: Line 435, If cows were sampled on precise days, it would be essential to provide detailed information regarding the sampling timeline. For example, if Day -21 was the target sampling day, the actual average, standard deviation, and range of days relative to this target should be specified. Similarly, for samples collected on Day 1, it would be important to report the range, average, and standard deviation of the number of hours elapsed since calving. I suggest including these details in the Methods and Materials section where they would fit best.
Response: Thank you for your suggestion. In fact, the detailed sampling times for Day -21 are already shown in Columns P-A of the supplementary dataset "CowInformation.csv." Consistently, Day 1 samples were collected on the second day after calving, regardless of the exact time of parturition. We have added the necessary details to the Materials and Methods section. (Lines 477-480)

Comment 17: Line 445: Standardize notation to "3,000 × g" (comma separator for thousands).
Response: As suggested, we have revised the problem. (Line 489)

Comment 18: Lines 504-505: Use Greek symbols: α-diversity and β-diversity (revise throughout the manuscript).
Response: Thank you for the suggestion. We have revised the entire manuscript to address the problems.

Comment 19: Line 527: Cite references for the residual analysis methodology.
Response: As suggested, we added the reference. (Line 601)

Comment 20: Lines 122 - 165: Use either "fecal microbiota" or "hindgut microbiota" consistently for intestinal - related descriptions.

Response: As suggested, we have replaced "hindgut microbiota" with "fecal microbiota" throughout the manuscript.
Comment 21: Lines 831 - 834: Increase the font size in Sanky and Markov chain diagrams of Figures 3 and 4 for better readability.
Response: As suggested, we have revised the relevant content to enhance readability.

Editorial Manager®

Cookie Preference Center

We use cookies which are necessary to make our site work. We may also use additional cookies to analyze, improve and personalize our content and your digital experience. For more information, see our Cookie Policy.

You may choose not to allow some types of cookies. However, blocking some types may impact your experience of our site and the services we are able to offer. See the different category headings below to find out more or change your settings.

Allow all

Manage Consent Preferences

Strictly Necessary Cookies

Always active

These cookies are necessary for the website to function and cannot be switched off in our systems. They are usually only set in response to actions made by you which amount to a request for services, such as setting your privacy preferences, logging in or filling in forms. You can set your browser to block or alert you about these cookies, but some parts of the site will not then work. These cookies do not store any personally identifiable information.

Functional Cookies

These cookies enable the website to provide enhanced functionality and personalisation. They may be set by us or by third party providers whose services we

have added to our pages. If you do not allow these cookies then some or all of these services may not function properly.

Performance Cookies

These cookies allow us to count visits and traffic sources so we can measure and improve the performance of our site. They help us to know which pages are the most and least popular and see how visitors move around the site.

Targeting Cookies

These cookies may be set through our site by our advertising partners. They may be used by those companies to build a profile of your interests and show you relevant adverts on other sites. If you do not allow these cookies, you will experience less targeted advertising.

Cookie List

Clear

Apply Cancel

Consent Leg.Interest

Confirm my choices

Powered by onetrust
