# Supplementary material for: Deterministic succession patterns in the rumen and fecal microbiome associate with host metabolic shifts in peripartum dairy cattle
Source: Gigascience. 2025 May 19;14:giaf042. doi: 10.1093/gigascience/giaf042 (PMC12087452; doi:10.1093/gigascience/giaf042)
Supplement: giaf042_Reviewer_1_Report_original_submission — Mst.Sogra Banu Juli -- 11/14/2025 [file giaf042_reviewer_1_report_original_submission.pdf]

Reviewer Report

Title: Deterministic Succession Patterns in the Rumen and Fecal Microbiome Associate with host Metabolic Shifts in Periparturient Dairy Cattle

Version: Original Submission **Date:** 11/14/2024

Reviewer name: Mst.Sogra Banu Juli

Reviewer Comments to Author:

Thanks to all author for the excellent study.
1. It was good to see if the authors collected sample 7 day of prepartum because 21 days of prepartum is a long time which is hard to estimate the changes over 21-day prepartum to 0 day.
2. It is not clearly mentioned in the methodology which sequencing data used for which analysis.
3. Did author observe any correlation between provided food and changes in rumen and hindgut microbiome.
Title "Deterministic Succession Patterns of Rumen and Hindgut Microbiome Driving Host Metabolism in Periparturient Ruminants" is not clearly presenting the study. I would suggest modifying.
Line 23: which platform of metagenome sequencing.
Line 36: Significantly (which test)
Line 51: please provide exact definition of periparturient period with reference.
Line 63: Sentence is not complete.
Line 64: over "the" time
Line 69: to understand
Line 71-75: Provide more example of cattle and cow. Study on pregnant women is not relating the earlier sentence.
Line: 97-107: these are the part of methodology. I will suggest avoiding in introduction section.
Line: 115: Which timepoint or days
Line: 117: Which type of drastic change, decrease or increase
Line: 124: which results?
Line133: Please describe clearly the pattern of genera depending on the pre and postpartum stages. RDMM1 prominently appeared until 7 d postpartum and what in 14 and 21 days of postpartum? Similarly, RDMM2 and RDMM7 were increasing with the timepoints of post-partum.
Line 140: use past tense and over "the" time and full stop at the end.
Line 141-142: Remove third bracket.
Line 141: RDMM7 started to appear on 3 d postpartum and gradually increase over the timepoints.
Line 143-145: It is not clear to me. What does timeline of dominance shift mean?
Line 146: What type of instability observed?
Line 147: periparturient age points?
Line 151: "Similarly, FDMMs 151 exhibited distinct occurrence windows and dominant genera (Figs. 2h and i)" sentence is not complete, occurrence based on?
Line 154: disappeared "at" postpartum

Line 155: after postpartum or with postpartum.
Line 155: "with FDMM2 being characterized by 156 Rikenellaceae_RC9_gut_group, Muribaculaceae, and Alistipes, and FDMM3 by 157 Bacteroidales_RF16_group and Prevotellaceae_UCG_003" use separate sentence.
Line 159: with a peak at 3 d postpartum.
Line 151-160: clearly mention which figures are related. Are those lines not related with figure 2h if yes then which figure is related with community structure. Please make sure figures are mentioned clearly after each description or before description.
Line 163-164: RDMM and FDMM are not dominated with similar type of genus in each cluster, so comparison between them has no meaning.
Line 167: Did you include all 91 animals?
Line 175-176: Please mention the percentage of transformation over the time.
Line 180: How is it suggesting a return to stability?
Line 182: What does "microbial community types" mean?
Line 185: What are self-transition rates?
Line 195-196: "previously mentioned genera, including Rikenellaceae_RC9_gut_group, Prevotella, Acetitomaculum, and F082" this line is not clear.
Line 201-203: this could be the part of discussion.
Line 208: In RDMM analysis, author used top100 and in FDMM top 120 ASVs-is there any reason of this change?
Line 220: both rumen and faeces.
Line 222-223: Sentence is not clear.
Line 268: What does last time point, please specify.
Line 273: What does "residuals" mean, please define.
Line 320: "Systematically investigate" is that correct terminology to describe the trial?
Line 336-338: Avoid repetition of the methodology.
Line 379: Affect
Line 381: Stage means post-partum stage?
427: typo, bran or barn?
443: Specification of the tube.
449: Did author used similar method and kit of DNA extraction from rumen and faeces samples.
511: RDMM and FDMM clusters were defined based on 16S sequencing or metagenomics data? And what is the lowest Laplace approximation score?
536: What does R threshold mean?
539-540: the sentence is not clear.
550: specification of each kit
551: Briefly describe the procedure of ELISA briefly.
571: Which individual factors?
572: How the false discovery rate adjusted?
Figure 7: In x axis did not use the same value which affecting the Stack bars. I will suggest y to make similar values in x-axis.
Figure 3b and 4b: What does three stage means and how they defined "stabilization, transition and

stabilization"?

Methods

Are the methods appropriate to the aims of the study, are they well described, and are necessary controls included? Choose an item.

Conclusions

Are the conclusions adequately supported by the data shown? Choose an item.

Reporting Standards

Does the manuscript adhere to the journal's guidelines on minimum standards of reporting? Choose an item.

Choose an item.

Statistics

Are you able to assess all statistics in the manuscript, including the appropriateness of statistical tests used? Choose an item.

Quality of Written English

Please indicate the quality of language in the manuscript: Choose an item.

Declaration of Competing Interests

Please complete a declaration of competing interests, considering the following questions:

- Have you in the past five years received reimbursements, fees, funding, or salary from an organisation that may in any way gain or lose financially from the publication of this manuscript, either now or in the future?
- Do you hold any stocks or shares in an organisation that may in any way gain or lose financially from the publication of this manuscript, either now or in the future?
- Do you hold or are you currently applying for any patents relating to the content of the manuscript?
- Have you received reimbursements, fees, funding, or salary from an organization that holds or has applied for patents relating to the content of the manuscript?
- Do you have any other financial competing interests?
- Do you have any non-financial competing interests in relation to this paper?

If you can answer no to all of the above, write 'I declare that I have no competing interests' below. If your reply is yes to any, please give details below.

I declare that I have no competing interests.

I agree to the open peer review policy of the journal. I understand that my name will be included on my report to the authors and, if the manuscript is accepted for publication, my named report including any attachments I upload will be posted on the website along with the authors' responses. I agree for my report to be made available under an Open Access Creative Commons CC-BY license (http://creativecommons.org/licenses/by/4.0/). I understand that any comments which I do not wish to be included in my named report can be included as confidential comments to the editors, which will not be published.

Choose an item.

To further support our reviewers, we have joined with Publons, where you can gain additional credit to further highlight your hard work (see: https://publons.com/journal/530/gigascience). On publication of this paper, your review will be automatically added to Publons, you can then choose whether or not to claim your Publons credit. I understand this statement.

Yes Choose an item.
