# Supplementary material for: Deterministic succession patterns in the rumen and fecal microbiome associate with host metabolic shifts in peripartum dairy cattle
Source: Gigascience. 2025 May 19;14:giaf042. doi: 10.1093/gigascience/giaf042 (PMC12087452; doi:10.1093/gigascience/giaf042)
Supplement: giaf042_Reviewer_2_Report_original_submission — Shengru Wu -- 2/23/2025 [file giaf042_reviewer_2_report_original_submission.pdf]

Reviewer Report

Title: Deterministic Succession Patterns in the Rumen and Fecal Microbiome Associate with host Metabolic Shifts in Periparturient Dairy Cattle

Version: Original Submission **Date:** 2/23/2025

Reviewer name: Shengru Wu

Reviewer Comments to Author:

This study provides a comprehensive longitudinal analysis of rumen and hindgut microbiome dynamics in periparturient dairy cows, linking microbial succession patterns to host metabolic phenotypes. The work is well-structured, methodologically rigorous, and addresses a critical gap in understanding how gut microbiota transitions during the periparturient period influence metabolic health. The integration of high-resolution temporal sampling, ecological modeling, and mediation analysis strengthens the novelty and impact of the findings. However, some aspects of experimental design, statistical reporting, and interpretation require clarification or expansion before publication.

Major Comments

1.Why were rumen and fecal samples studied separately when analyzing the dynamic succession of the gut microbiota and its relationship with host metabolism in periparturient cows? Not as a whole. Please clarify.

2.The causal inference that "microbial communities drive host metabolic changes" requires cautious phrasing. Although mediation analysis was used to infer microbial-driven metabolic alterations, the observational nature of the study inherently limits the ability to exclude confounding factors, rendering causal claims speculative. The authors must revise such statements to avoid overstating conclusions.

3.For host indicators, please explain why only energy metabolism, liver function, and antioxidant indicators were analyzed.

4.Key methodological details are missing, hindering reproducibility. For example, descriptions of ICAMP and mediation analysis are insufficient.

5.The Discussion lacks depth. Expand the interpretation of microbial succession patterns and host-microbe interactions observed in the results.

Minor Comments

1.Title: Overly verbose. Shorten to emphasize the core focus and novelty. Replace "Periparturient Ruminants" with "Periparturient Dairy Cows" for precision.

2.Line 24: Revise "ecological modeling" to "ecological model" for grammatical consistency.

3.Abbreviations:

Define "RQUICKI-BHB" in full upon first mention: Revised Quantitative Insulin Sensitivity Check Index-BHB.

Provide the full name for "MaAsLin2": Multivariate Analysis by Linear Models.

Ensure all abbreviations (e.g., TMR, DMM, ICAMP) are defined at their first occurrence.

4.Line 117: Replace the colloquial phrase "drastic changes" with "significant changes" for scientific rigor.

5.Line 123: Explicitly annotate the PCoA plot to display the variance percentages (26.14% and 24.96%).

6.Line 124: The sentence "These results highlight their individualized states..." is interpretive and belongs in the Discussion, not Results.
7.Line 126 Justify the use of the Dirichlet Multinomial Mixture (DMM) model instead of the more common Partitioning Around Medoids (PAM) method for clustering.
8.Terminology clarity: Replace "DMM" with "cluster" throughout the text to improve readability, unless the method itself is a key focus.
9.Line 420-428 Periparturient cows usually receive postpartum care. What is the care plan in this experiment?
10.Line 434 Clarify whether all 91 cows completed sampling at all six time points. Provide details on sample loss rates or exclusion criteria
11.Line 435, If cows were sampled on precise days, it would be essential to provide detailed information regarding the sampling timeline. For example, if Day -21 was the target sampling day, the actual average, standard deviation, and range of days relative to this target should be specified. Similarly, for samples collected on Day 1, it would be important to report the range, average, and standard deviation of the number of hours elapsed since calving. I suggest including these details in the Methods and Materials section where they would fit best.
12.Line 445: Standardize notation to "3,000 Ã— g" (comma separator for thousands).
13.Lines 504-505: Use Greek symbols: Î±-diversity and Î²-diversity (revise throughout the manuscript).
14.Line 527: Cite references for the residual analysis methodology.
15.Lines 122 - 165: Use either "fecal microbiota" or "hindgut microbiota" consistently for intestinal - related descriptions.
16.Lines 831 - 834: Increase the font size in Sanky and Markov chain diagrams of Figures 3 and 4 for better readability.

Methods

Are the methods appropriate to the aims of the study, are they well described, and are necessary controls included? Choose an item.

Conclusions

Are the conclusions adequately supported by the data shown? Choose an item.

Reporting Standards

Does the manuscript adhere to the journal’s guidelines on minimum standards of reporting? Choose an item.

Choose an item.

Statistics

Are you able to assess all statistics in the manuscript, including the appropriateness of statistical tests used? Choose an item.

Quality of Written English

Please indicate the quality of language in the manuscript: Choose an item.

Declaration of Competing Interests

Please complete a declaration of competing interests, considering the following questions:

- Have you in the past five years received reimbursements, fees, funding, or salary from an organisation that may in any way gain or lose financially from the publication of this manuscript, either now or in the future?
- Do you hold any stocks or shares in an organisation that may in any way gain or lose financially from the publication of this manuscript, either now or in the future?
- Do you hold or are you currently applying for any patents relating to the content of the manuscript?
- Have you received reimbursements, fees, funding, or salary from an organization that holds or has applied for patents relating to the content of the manuscript?
- Do you have any other financial competing interests?
- Do you have any non-financial competing interests in relation to this paper?

If you can answer no to all of the above, write 'I declare that I have no competing interests' below. If your reply is yes to any, please give details below.

I declare that I have no competing interests

I agree to the open peer review policy of the journal. I understand that my name will be included on my report to the authors and, if the manuscript is accepted for publication, my named report including any attachments I upload will be posted on the website along with the authors' responses. I agree for my report to be made available under an Open Access Creative Commons CC-BY license (http://creativecommons.org/licenses/by/4.0/). I understand that any comments which I do not wish to be included in my named report can be included as confidential comments to the editors, which will not be published.

Choose an item.

To further support our reviewers, we have joined with Publons, where you can gain additional credit to further highlight your hard work (see: https://publons.com/journal/530/gigascience). On publication of this paper, your review will be automatically added to Publons, you can then choose whether or not to claim your Publons credit. I understand this statement.

Yes Choose an item.
